# Structural Diversity and Bioactivities of Peptaibol Compounds From the Longibrachiatum Clade of the Filamentous Fungal Genus *Trichoderma*

**DOI:** 10.3389/fmicb.2019.01434

**Published:** 2019-06-26

**Authors:** Tamás Marik, Chetna Tyagi, Dóra Balázs, Péter Urbán, Ágnes Szepesi, László Bakacsy, Gábor Endre, Dávid Rakk, András Szekeres, Maria A. Andersson, Heidi Salonen, Irina S. Druzhinina, Csaba Vágvölgyi, László Kredics

**Affiliations:** ^1^Department of Microbiology, Faculty of Science and Informatics, University of Szeged, Szeged, Hungary; ^2^Department of General and Environmental Microbiology, Faculty of Sciences, and Szentágothai Research Center, University of Pécs, Pécs, Hungary; ^3^Department of Plant Biology, Faculty of Science and Informatics, University of Szeged, Szeged, Hungary; ^4^Department of Civil Engineering, Aalto University, Espoo, Finland; ^5^Research Area Biochemical Technology, Institute of Chemical, Environmental and Bioscience Engineering, TU Wien, Vienna, Austria; ^6^Jiangsu Provincial Key Laboratory of Organic Solid Waste Utilization, Nanjing Agricultural University, Nanjing, China

**Keywords:** *Trichoderma*, Longibrachiatum, peptaibol, brevicelsin, mass spectrometry, antifungal activity, *Arabidopsis*, mammalian cells

## Abstract

This study examined the structural diversity and bioactivity of peptaibol compounds produced by species from the phylogenetically separated Longibrachiatum Clade of the filamentous fungal genus *Trichoderma*, which contains several biotechnologically, agriculturally and clinically important species. HPLC-ESI-MS investigations of crude extracts from 17 species of the Longibrachiatum Clade (*T. aethiopicum, T. andinense, T. capillare, T. citrinoviride, T. effusum, T. flagellatum, T. ghanense, T. konilangbra, T. longibrachiatum, T. novae-zelandiae, T. pinnatum, T*. *parareesei, T. pseudokoningii, T. reesei, T. saturnisporum, T. sinensis*, and *T. orientale*) revealed several new and recurrent 20-residue peptaibols related to trichobrachins, paracelsins, suzukacillins, saturnisporins, trichoaureocins, trichocellins, longibrachins, hyporientalins, trichokonins, trilongins, metanicins, trichosporins, gliodeliquescins, alamethicins and hypophellins, as well as eight 19-residue sequences from a new subfamily of peptaibols named brevicelsins. Non-ribosomal peptide synthetase genes were mined from the available genome sequences of the Longibrachiatum Clade. Their annotation and product prediction were performed *in silico* and revealed full agreement in 11 out of 20 positions regarding the amino acids predicted based on the signature sequences and the detected amino acids incorporated. Molecular dynamics simulations were performed for structural characterization of four selected peptaibol sequences: paracelsins B, H and their 19-residue counterparts brevicelsins I and IV. Loss of position R6 in brevicelsins resulted in smaller helical structures with higher atomic fluctuation for every residue than the structures formed by paracelsins. We observed the formation of highly bent, almost hairpin-like, helical structures throughout the trajectory, along with linear conformation. Bioactivity tests were performed on the purified peptaibol extract of *T*. *reesei* on clinically and phytopathologically important filamentous fungi, mammalian cells, and *Arabidopsis thaliana* seedlings. Porcine kidney cells and boar spermatozoa proved to be sensitive to the purified peptaibol extract. Peptaibol concentrations ≥0.3 mg ml^−1^ deterred the growth of *A*. *thaliana*. However, negative effects to plants were not detected at concentrations below 0.1 mg ml^−1^, which could still inhibit plant pathogenic filamentous fungi, suggesting that those peptaibols reported here may have applications for plant protection.

## Introduction

At present, more than 300 species of the genus *Trichoderma* (Ascomycota, Hypocreales, Hypocreaceae) have been described (Bissett et al., 2015; Zhang and Zhuang, 2018). The majority of these species were described after the year 2000, as only a few species were initially included in the genus (Bisby, 1939; Rifai, 1969). Section Longibrachiatum of the genus was one of the five *Trichoderma* sections according to Bissett (1984, 1991a,b,c). It forms a monophyletic group phylogenetically separated from the other four *Trichoderma* sections (Kuhls et al., 1997; Samuels et al., 1998) and is designated recently as the Longibrachiatum Clade (Samuels et al., 2012). It is one of the youngest clades of the genus (Kubicek et al., 2011) and has the largest number of available whole-genome sequence data. This clade is ecologically highly versatile as it contains prominent clinically relevant and ecologically restricted species. *Trichoderma longibrachiatum, T*. *orientale*, and *T*. *citrinoviride* are opportunistic human pathogens causing infections, mainly in immunocompromised patients (Kuhls et al., 1999; Kredics et al., 2003; Hatvani et al., 2013). *T*. *longibrachiatum* or its transformants have also been suggested for use as biocontrol agents against plant pathogens like *Pythium ultimum* or members of the *Fusarium solani* species complex (Migheli et al., 1998; Rojo et al., 2007). *T. longibrachiatum* and *T*. *orientale* are sympatric species but have different reproductive strategies, the former being strictly clonal, whereas the latter recombines sexually (Druzhinina et al., 2008). The cellulase producer *T*. *reesei* is also capable of sexual reproduction (Seidl et al., 2009), whereas its sympatric species *T*. *parareesei* is genetically isolated and has a clonal lifestyle (Atanasova et al., 2010; Druzhinina et al., 2010). While *T*. *longibrachiatum* and *T*. *orientale* are cosmopolitan, the related *T. pinnatum* and *T*. *aethiopicum* are rare and restricted species (Druzhinina et al., 2010). Numerous other species, including *T*. *reesei, T*. *parareesei, T*. *pseudokoningii, T*. *sinense, T*. *effusum, T*. *konilangbra, T*. *andinense*, or *T*. *novae-zelandiae* are also geographically restricted (Druzhinina et al., 2012).

Several secondary metabolites are produced by *Trichoderma* species from the Longibrachiatum Clade. Probably the best known species is *T*. *reesei*, which produces hydrolytic enzymes degrading cellulose or hemicellulose (Harman and Kubicek, 1998; Kubicek et al., 2009). Peptaibols are membrane-active compounds with the ability to aggregate and form ion channels in lipid bilayer membranes. They are usually short peptides of 8–20 residues with non-proteinogenic amino acids and are biosynthesised by non-ribosomal peptide synthetases (NRPSs) (Marahiel, 1997; Marahiel et al., 1997; May et al., 2002; Degenkolb et al., 2003, 2007; Bushley and Turgeon, 2010; Marik et al., 2017b). In the case of NRPSs, a single large protein is responsible for the activation, incorporation and elongation of the peptides. NRPSs can also incorporate non-proteinogenic residues, thus increasing the chemical diversity of the products. The lack of specificity of the recognition sites and the three-dimensional structure of the enzyme lead to the acceptance of closely related residues (such as Vxx vs. Lxx). Consequently, the number of positionally isomeric and homologous peptaibols biosynthesised by a single NRPS can be large. The repair mechanisms, which usually operate during biosynthesis, are also absent in NRPS pathways, thus further increasing the variability of the products. Characteristic residues of peptaibols include α-aminoisobutyric acid (Aib) and isovaline (Iva), as well as 1,2-amino alcohols such as Leuol, Valol, Pheol, Tyrol, Ileol, Alaol, and Prool at the C-terminus (Degenkolb et al., 2008; Stoppacher et al., 2013). Peptaibols usually form short, linear helical structures, several of which aggregate to form ion channels and may damage lipid membranes. Investigation of the structural and dynamic properties of peptaibol molecules is important for the understanding of their biological activities. Computational molecular dynamics-based simulation is a popular technique for investigating a molecule's dynamic behavior and predicting its three-dimensional structure. Peptaibols like trichobrachins (Násztor et al., 2013), harzianins (Putzu et al., 2017), alamethicin (Leitgeb et al., 2007; Kredics et al., 2013), tripleurin (Tyagi et al., 2019), and others have been investigated using such techniques. Knowledge about the structure of peptaibols might also facilitate the design of bioactive peptides for future applications. The characteristic non-proteinogenic amino acid residues of peptaibols (Aib and C-terminal alcohols) can be parameterised quantum-mechanically, and the effects of their presence can be evaluated. In general, long molecular time scales are required to effectively simulate peptide folding processes. An all-atom enhanced sampling technique known as accelerated molecular dynamics (aMD) can be used, which provides a non-negative boost to the potential energy and speeds up the process of peptide folding.

*Trichoderma* species are widely used against various plant pathogenic fungi as biocontrol agents because of their fast growth and reproduction, their mycoparasitism and their production of secondary metabolites (Chaverri et al., 2015; Degenkolb et al., 2015; Waghunde et al., 2016). Species like *T*. *viride, T*. *virens, T*. *atroviride, T*. *asperellum*, and *T*. *harzianum* are frequently studied due to their production of enzymes and antibiotics valuable in agriculture (Schuster and Schmoll, 2010; Contreras-Cornejo et al., 2016) and their antagonistic effects against pathogenic fungi such as *Botrytis cinerea, Alternaria solani* and *Rhizoctonia solani* (Harman et al., 2004). Incubation of a “*T*. *harzianum*” strain later re-identified as *T. atroviride* (Röhrich et al., 2014) with *B*. *cinerea* cell walls resulted in the secretion of cell wall hydrolytic enzymes and antibiotic fractions of peptaibols, which inhibited *B*. *cinerea* spore germination, causing a fungicidal effect. Peptaibols and hydrolytic enzymes were found to work synergistically in this antagonistic interaction (Schirmböck et al., 1994).

*Trichoderma* species also interact with plants through secondary metabolites. Although several studies reported positive effects of *Trichoderma* species on the physiological and biochemical responses of plants (Contreras-Cornejo et al., 2016), inhibition of plant growth and primary root development have also been described (Rippa et al., 2010; Shi et al., 2016). The most thoroughly investigated model plant, *Arabidopsis thaliana*, is frequently used to test the bioactivity of the secondary metabolites of *Trichoderma* species (Kottb et al., 2015). Peptaibols can induce auxin production and disruption of the auxin response gradient in root tips (Shi et al., 2016). The most thoroughly studied peptaibol, alamethicin, was shown to induce resistance in plants (Leitgeb et al., 2007; Kredics et al., 2013) but can also be toxic, causing lesions on *Arabidopsis* leaves (Rippa et al., 2010). However, it should also be considered that the commercially available alamethicin mixture (Sigma-Aldrich A4665) may also contain the trichothecene-type mycotoxin harzianum A produced by the strain *T. brevicompactum* used for alamethicin fermentations (Degenkolb et al., 2006).

This study aimed at revealing the genomic background, structural diversity and bioactivity of peptaibol compounds produced by different species from the ecologically diverse Longibrachiatum Clade of the genus *Trichoderma*.

## Materials and Methods

### Strains and Culture Conditions

Twenty-two strains from 17 *Trichoderma* species belonging to the Longibrachiatum Clade of the genus were selected from the TU Collection of Industrially Important Microorganisms, Vienna, Austria (TUCIM, www.vt.tuwien.ac.at/tucim/) and the Szeged Microbiology Collection, Szeged, Hungary (SzMC; www.szmc.hu) for investigation of their peptaibol production (Table 1). For testing the antifungal activity of peptaibol extracts, filamentous fungal strains of clinical relevance (*Aspergillus fumigatus* SzMC 23245, *Fusarium falciforme* SzMC 11407 and *Fusarium keratoplasticum* SzMC 11414 from human keratomycosis, India) or phytopathological relevance (*Alternaria alternata* SzMC 16085, *F. solani* species complex SzMC 11467 and *Phoma cucurbitacearum* SzMC 16088) were selected. The strains were maintained and cultured as described by Marik et al. (2017a).

**Table 1:** *Trichoderma* strains from the Longibrachiatum Clade involved in the study.

**SzMC identifier**	**Other identifier**	Subclade^*^	**Species**	**Origin**	**References**
1773	CECT 2412	Longibrachiatum/Orientale	*T. longibrachiatum*	Mushroom compost, Wales	Druzhinina et al., 2008
1775	CECT 2937	Longibrachiatum/Orientale	*T. longibrachiatum*	Antarctica	Kuhls et al., 1997
1776	CECT 20105	Longibrachiatum/Orientale	*T. longibrachiatum*	Biocontrol strain, Spain	Antal et al., 2005
12546	UAMH 7956	Longibrachiatum/Orientale	*T. longibrachiatum*	Bone marrow transplant recipient	Richter et al., 1999
12556	UAMH 9573	Longibrachiatum/Orientale	*T. orientale*	Peritoneal catheter tip, Canada	Kredics et al., 2003
22602	TUCIM 1817	Longibrachiatum/Orientale	*T. aethiopicum*	*Coffea arabica* rhizosphere; Jimma, Ethiopia	Druzhinina et al., 2008
22603	TUCIM 3421	Longibrachiatum/Orientale	*T. pinnatum*	Sri Lanka	Samuels et al., 2012
22614	TUCIM 917, QM6a	Parareesei/Reesei	*T. reesei*	canvas of US army; Solomon Islands	Reese et al., 1950
22616	QM9414	Parareesei/Reesei	*T. reesei*	Mutant of QM9123 (which is mutant of QM6a)	Kuhls et al., 1996
22617	QM9414 G2*Δlae*1	Parareesei/Reesei	*T. reesei*	*lae1* null mutant (Δ*lae1*) of *T. reesei* QM9414	Seiboth et al., 2012
22615	TUCIM 661	Parareesei/Reesei	*T. parareesei*	Subtropical rain forest; Iguazu Falls, Argentina	Atanasova et al., 2010
22606	TUCIM 1267	Saturnisporum	*T. saturnisporum*	Italy	Samuels et al., 2012
22607	TUCIM 132	Konilangbra/Sinensis	*T. konilangbra*	Uganda	Samuels et al., 1998
22608	TUCIM 3350	Konilangbra/Sinensis	*T. flagellatum*	*Coffea arabica* rhizosphere; Ethiopia	Belayneh Mulaw et al., 2010
22609	TUCIM 527	Konilangbra/Sinensis	*T. sinensis*	Taiwan	Bissett et al., 2003
22618	SJ40	Citrinoviride/Pseudokoningii	*T. citrinoviride*	Office bookshelf, settled dust, Espoo, Finland	Castagnoli et al., 2018
22613	TUCIM 1277	Citrinoviride/Pseudokoningii	*T. pseudokoningii*	the bark of *Beilschmiedia tawa*	Samuels et al., 1998
22612	TUCIM 4158	Novae-zelandiae/ Saturnisporopsis	*T. novae-zelandiae*	Native *Notophagus* forest, New Zealand	Samuels et al., 1998
22604	TUCIM 2057		T. ghanense^**^	*Agaricus* compost; Hungary	Hatvani et al., 2007
22605	TUCIM 2883		T. capillare^**^	Wall of a mushroom growing cellar; Hungary	Hatvani et al., 2007
22610	TUCIM 1291		T. andinense^**^	Venezuela, high elevation	Samuels et al., 1998
22611	TUCIM 254		T. effusum^**^	Soil isolation; Himalaya, India	Bissett et al., 2003

* *Subclades were defined based on Samuels et al. (2012)*.

** *Considered as lone lineages*.

### Peptaibol Extraction

Peptaibols were extracted according to Marik et al. (2017a). For large quantity peptaibol production and purification, *T*. *reesei* QM9414 (SzMC 22616) was cultured according to Marik et al. (2018). The samples were purified on a Flash chromatograph (CombiFlash EZ Prep UV-VIS Teledyne Isco). The cartridge (CombiFlash EZ Prep) was filled with 60 cm^3^ silica (30–40 μm), and 1.5 g of crude peptaibol extract was applied above the septum. The flow rate was set to 35 ml min^−1^ and the wavelength of the UV detector to 270/320 nm. Solvents A and B were chloroform and methanol, respectively (gradient solvent B: 0%, 0 min; 0%, 5 min; 100%, 15 min; 100%, 18 min). Fractions were automatically collected into collector tubes (18 × 180 mm, 30 ml) based on the slope of the UV signal. Fractions were evaporated, dissolved in methanol (100 mg ml^−1^) and stored at −20°C. The purity of the samples was checked by HPLC-MS as described by Van Bohemen et al. (2016). For this analysis, the appearing y_7_-ion fragments were quantified and compared to alamethicin (Sigma-Aldrich A-4665, Hungary) dissolved in methanol (VWR, Hungary).

### Analytical Procedures and Data Analysis

Crude peptaibol extracts were subjected to HPLC-ESI-MS using a Varian 500 MS equipment with the parameters described previously (Marik et al., 2018). The excitation storage level (*m/z*)/excitation amplitude (V) conditions during the MS^2^ measurements of selected y_7_ fragments were: *m/z* of 774.4 (209.4/3.02), *m/z* of 775.4 (209.7/3.03), *m/z* of 788.4 (212.9/3.08), and *m/z* of 789.4 (213.2/3.08). The method of peptaibol identification followed the protocol described previously by Marik et al. (2013, 2017a). The initial Varian 500 MS data were further confirmed by HPLC-Orbitrap-MS: Dionex UltiMate 3000 system (Thermo Scientific, CA, USA) controlled by the Xcalibur 4.2 software (Thermo Scientific, CA, USA) and equipped with a quaternary pump, a vacuum degasser, an autosampler and a column heater. Gemini NX-C18 HPLC column (50 × 2.0 mm, 3 μm; Phenomenex Inc., Torrance, CA, USA) was used for the separation. Solvent A was H_2_O:MeOH:MeCN 8:1:1 with 10 mM ammonium-acetate and 0.1% (*v/v*) acetic acid, while solvent B was acetonitrile/methanol 1:1 (*v/v*) with 10 mM ammonium-acetate and 0.1% (*v/v*) acetic acid. The flow rate was set to 0.2 ml min^−1^ and the gradient program for Solvent B was 10%−0 min, 10%−2 min, 78%−3 min, 89%−16 min, 95%−16.5 min, 95%−19.5 min, 10%−20 min, 10%−24 min. The column temperature was kept at 30°C and the injection volume was 5 μl. An Orbitrap-MS: Thermo Scientific Q Exactive Plus (Thermo Scientific, CA, USA) with HESI source in positive mode controlled by Xcalibur 4.2 software (Thermo Scientific, CA, USA) was used for the MS measurements. The HESI parameters were: spray voltage−3 kV, sheath gas flow rate−30 arbitrary units, aux gas flow rate−15 arbitrary units, capillary temperature−350°C, aux gas heater−250°C. The acquisition mode was Full-MS-ddMS^2^. Full-MS paramteres were: resolution−70,000 at *m/z* 200, AGC target−3e6, maximum injection time−100 ms, scan range−350-2200 *m/z*. The ddMS^2^ parameters: fixed first scan at *m/z* 80, resolution 17500 at *m/z* 200, AGC target−1e6, maximum injection time−50 ms, isolation window−1 *m/z*, collusion energy−30 NCE. The minimum AGC target for ddMS^2^ triggering was 1e5. As no amino acid analysis was carried out for the determination of the Val/Iva and Leu/Ile isomers, the Vxx/Lxx nomenclature was used in the peptaibol sequences. The newly identified peptaibol compounds were named according to the group to which they belong (A or B) and the elution order of the compounds on the HPLC-Varian MS system (I, II, …, *n*), appended to “Pept.” Compounds with the same retention time but different sequences were considered as variants and named with small latin letters (a, b, …, *n*; in decreasing order of amount the variants were produced). Group C peptaibols were named as brevicelsins and numbered according to their elution order.

Peptaibol profiles of individual strains were analyzed using cluster analysis in the ClustVis web tool (Metsalu and Vilo, 2015), and a heat map was constructed using the complete linkage and Euclidian distance settings applied to the columns (strains).

Degenkolb et al. (2006) reported that the Sigma alamethicin standard (A-4665) may be contaminated by the trichothecene mycotoxin harzianum A. In the case of the batch used in this study as a reference compound, the detection of harzianum A was carried out based on a previous article (Nielsen et al., 2005). The flow rate was set to 0.2 ml min^−1^ on a Phenomenex Gemini 50 × 2 mm, 3 μm HPLC column. The column heater was set to 30°C and the injection volume was 5 μl. An Orbitrap-MS detector was attached to the HPLC system and the parameters were set according to the Orbitrap MS parameters described above. The measurements ran in negative ionization mode, the spray voltage was set to −3 kV.

### Bioinformatic Analysis of Peptaibol Synthetase Genes

Peptaibol synthetases of *Trichoderma* species from the Longibrachiatum Clade with accessible full genome sequences, *T*. *reesei, T*. *parareesei*, and *T*. *citrinoviride* (GenBank Assembly accession numbers GCA_000167675.2, GCA_001050175.1 and GCA_003025115.1, respectively) and two strains of *T*. *longibrachiatum* (GCA_003025155.1, GCA_000332775.1) were identified using the Secondary Metabolites from InterProScan (SMIPS) online software, and 20 as well as 14 module NRPSs were selected (Wolf et al., 2016). In the case of *T*. *longibrachiatum, T*. *citrinoviride, T*. *reesei*, and *T*. *parareesei*, the extracted sequences were analyzed using the Antibiotics and Secondary Metabolites Analysis Shell (antiSMASH), the PKS-NRPS Analysis Web-site, the NRPS/PKS substrate predictor and the NRPSPredictor3 SVM, as described by Marik et al. (2017a).

### Accelerated Molecular Dynamics Simulations of 20- and 19-Residue Peptaibols

Calculation of the partial charges for the non-standard residues Aib and Pheol and the preparation of unfolded conformations of four selected peptaibols in water were carried out as described by Tyagi et al. (2019). The Leu and Val positions in brevicelsin sequences were predicted based on their positionally isomeric 20-residue paracelsin counterparts. For the Paracelsin B system, 3910 water molecules were added with a box size of 55.05 × 46.82 × 62.33 Å and a volume of 160676.0 Å^3^, whereas 3557 TIP3P water molecules were added with a box size of 55.05 × 42.11 × 63.40 Å and a volume of 147021.35 Å^3^ to prepare the Paracelsin H system. Similarly, 4725 water molecules were added to the Brevicelsin I system with a box size of 67.57 × 50.93 × 54.97 Å and a volume of 189190.34 Å^3^, whereas 4536 water molecules were added to the Brevicelsin IV system with a box size of 68.52 × 45.96 × 58.30 Å and a volume of 183623.0 Å^3^.

The four systems were prepared for aMD simulations used to enhance sampling with a boost to the whole potential energy and an extra boost to torsional energy. The values of coefficients a_1_ and a_2_ were set to 4, whereas b_1_ and b_2_ were set to 0.16, based on previous studies (Pierce et al., 2012).

### Peptaibol Bioactivity Assays

For inhibition tests with filamentous fungi, malt extract agar medium completed with yeast extract was used at 25°C, following the method described by Marik et al. (2018). The purified peptaibol extract of *T*. *reesei* QM9414 was tested in an agar plate well-diffusion assay with methanol as a control, as well as alamethicin (Sigma-Aldrich A-4665, Hungary) and nystatin (Nystatin 2-hydrate BioChemica, AppliChem A3811,0025, Germany) as reference compounds. All solutions were prepared in two-step dilution series from 0.4 mg ml^−1^ to 0.0036125 mg ml^−1^. The inhibition zones were measured as the distance between the edge of the fungal colonies and the edge of the holes containing the peptaibol solutions at the time when the edge of the colony reached the edge of the control hole filled with methanol. At the same time, plates were photographed with a Coolpix S2600 digital camera (Nikon). Minimum inhibitory concentration (MIC) values were defined as the lowest concentrations where an inhibition zone could be detected. Experiments were carried out in triplicate.

In order to investigate the biological effects of peptaibols on plants, *A. thaliana* (Col-0 ecotype) seeds were planted on 0.5 × Murashige and Skoog agar (8%) medium (Horváth et al., 2015) with the addition of 0.5% sucrose (w/v) (pH adjusted to 5.5 with NaOH) in plastic Petri dishes (90 × 17 mm) five seeds per Petri dish in one line. Seeds were surface sterilized with 70% ethanol for 1 min, treated with 4% hypochlorite for 15 min and washed with sterile distilled water. After vernalisation at 4°C for 24 h, seeds were sown onto the agar plates. *Arabidopsis* plants were placed in a greenhouse with a photoperiod of 12 h of light and 12 h of darkness, a light intensity of 300 μmol m^−2^ s^−2^ and a temperature of 25 ± 1°C. After the third day post germination, plates were placed at an angle of 50° to allow root growth along the agar surface and to promote aerial growth of the hypocotyls. Four 5 mm holes were bored with a sterile cork borer 0.5 cm from the root tips of 5-day-old *Arabidopsis* seedlings (five seedlings per plate) and filled with 40 μl of peptaibol extract. The growth of primary roots was measured every 24 h for 4 days. Photographs of 15-day-old plants were taken using a Coolpix S2600 digital camera (Nikon). The fresh weights of the plants from each plate were measured, and photosynthetic pigments were quantified as described by Lichtenthaler (1987). Statistical analyses were performed using Bonferroni's multiple comparison tests with the GraphPad Prism software version 6.00 (GraphPad Software, San Diego, CA, USA; www.graphpad.com) using 25 samples.

Bioassays using porcine kidney cells (PK-15) and assays of cell membrane integrity disruption in boar sperm cells were carried out as described previously (Bencsik et al., 2014; Marik et al., 2017b).

## Results

### Identification of Peptaibols Produced by *Trichoderma* Species From the Longibrachiatum Clade

Peptaibols produced by species from the Longibrachiatum Clade of genus *Trichoderma* were identified using the strategy described by Marik et al. (2013, 2017a). Extracted ion chromatograms (EIC) resulting from full scan measurements of crude extracts from the examined *Trichoderma* strains are shown in Supplementary Figures 1–22. Singly-charged pseudomolecular ions, such as [M+Na]^+^ or [M+H]^+^, were scarcely detectable in the spectra, whereas doubly charged ([M+2Na]^2+^) ions were present and could be used for identification. Full scan MS spectra contained the series of the fragment ions from the N-terminal part (b_1_—b_6_ and b_8_-b_13_, Supplementary Figure 23) except for b_7_, where the stable Gln-Aib bond is present in the compounds (Krause et al., 2006a). The C-terminal y_7_ fragment was consistently observed and provided a good reference for the quantification of the peptides in the mixture. The first 13 amino acid residues could be identified from the full scan MS spectra, but MS^2^ experiments were performed for the identification of residues at the C-terminus. The last four residues could be identified directly from the MS^2^ spectra (Supplementary Figure 24). The y_7_-AA(19-15) ions were not shown on these spectra, therefore another MS^2^ fragmentation was performed on an Orbitrap-MS system from the y_7_ ions, which proved Vxx and Aib in positions 15 and 16, respectively (Supplementary Figure 25). All the detected peaks could also be reidentified at high resolution on the HPLC-Orbitrap-MS system, except for y_7_-H_2_O (Supplementary Tables 1–6). Instead of [M+Na]^+^ and [M+2Na]^2+^ ions, [M+H]^+^ could be observed on these spectra.

The peptaibol sequences could be categorized into three groups, designated as A (Table 2; Supplementary Tables 1, 4), B (Table 3; Supplementary Tables 2, 5) and C (Table 4; Supplementary Tables 3, 6). Groups A and B contain 20-residue peptaibols, whereas group C sequences had lost a residue in position R6. The novelty of the sequences was validated according to the “Comprehensive Peptaibiotics Database” (Stoppacher et al., 2013) as well as the last, offline version of the “Peptaibiotics Database.” The former online resource (Neumann et al., 2015) is unavailable since the autumn of 2017, therefore PubMed searches of publications since 2017 were performed with the keyword “peptaibol.” Several sequences proved to be homologous or positionally isomeric to the peptaibol subfamilies of trichobrachins, paracelsins, suzukacillins, saturnisporins, trichoaureocins, trichocellins, longibrachins, hyporientalins, trichokonins, trilongins, metanicins, trichosporins, gliodeliquescins, alamethicins, and hypophellins. Some sequences had amino acid exchanges in comparison with previously described compounds from the peptaibol groups listed above.

**Table 2:** Sequences of the newly identified group A peptaibol compounds from *Trichoderma* species of the Longibrachiatum Clade and their similarities to known peptaibols available in the “Comprehensive Peptaibiotics Database.”

**Peptide**	**M**	**[M+Na]^**+**^**	**[M+2Na]^**2+**^**	**b_**13**_**	**y_**7**_**	**rt-GK (min)**	**R**	**R1**	**R2**	**R3**	**R4**	**R5**	**R6**	**R7**	**R8**	**R9**	**R10**	**R11**	**R12**	**R13**	**R14**	**R15**	**R16**	**R17**	**R18**	**R19**	**R20**	**Compound identical or positionally isomeric with**	**References**
Pept-A-Ia	1922	1945	984	1149	774	35.35	Ac	Aib	Ala	Aib	Ala	Aib	Ala	Gln	Aib	Vxx	Ala	Gly	Lxx	Aib	Pro	Vxx	Aib	Aib	Gln	Gln	Pheol	Trichoaureocin 1d	Brückner et al., 2002
Pept-A-Ib	1922	1945	984	1149	774	36.88	Ac	Aib	Ala	Aib	Ala	Aib	*Aib*	Gln	Aib	Vxx	Ala	Gly	*Vxx*	Aib	Pro	Vxx	Aib	Aib	Gln	Gln	Pheol	**New:** Trichoaureocin 1d: [Lxx]^12^ → [*Vxx*]^12^	Brückner et al., 2002
Pept-A-IIa	1923	1946	984.5	1149	775	38.26	Ac	Aib	Ala	Aib	Ala	Aib	Ala	Gln	Aib	Vxx	Aib	Gly	*Vxx*	Aib	Pro	Vxx	Aib	Aib	Glu	Gln	Pheol	**New**: Longibrachin B II: [Lxx]^12^ → [*Vxx*]^12^	Leclerc et al., 1998
																												**New**: Trilongin CI: [Lxx]^12^ → [*Vxx*]^12^	Mikkola et al., 2012
																												**New:** Hypophellin 2: [Lxx]12 → [*Vxx*]12	Röhrich et al., 2013
																												**New**: Longibrachin B II; Trilongin CI: [Lxx]^12^ → [*Vxx*]^12^	Tamandegani et al., 2016
Pept-A-IIb	1923	1946	984.5	1149	775	37.46	Ac	Aib	Ala	Aib	Ala	Aib	Ala	Gln	Aib	Vxx	Ala	Gly	Lxx	Aib	Pro	Vxx	Aib	Aib	*Glu*	Gln	Pheol	**New:** Trichoaureocin 1d: [Gln]^17^ → [*Glu*]^17^	Brückner et al., 2002
Pept-A-IIIa	1936	1959	991	1149	788	39.82	Ac	Aib	Ala	Aib	Ala	Aib	Ala	Gln	Aib	*Vxx*	Aib	Gly	*Vxx*	Aib	Pro	Vxx	Aib	Vxx	Gln	Gln	Pheol	**New**: Longibrachin A II: [Leu]^9^ → [*Vxx*]^9^	Leclerc et al., 1998
																												**New**: Paracelsin F: [Aib]^12^ → [*Vxx*]^12^	Pócsfalvi et al., 1997
																												**New**: Suzukacillin A 03: [Aib]^12^ → [*Vxx*]^12^	Krause et al., 2006b
																												**New**: Suzukacillin A 10a: [Lxx]^12^ → [*Vxx*]^12^	Krause et al., 2006b
																												**New**: Trichoaureocin 4: [Lxx]^12^ → [*Vxx*]^12^	Brückner et al., 2002
																												**New**: Trichobrachin II 07, 08, 09 IIb B: [Lxx]^12^ → [*Vxx*]^12^	Krause et al., 2007
																												**New**: Trichokonin VII: [Leu]^12^ → [*Vxx*]^12^	Huang et al., 1996
																												**New**: Trilongin BII: [Lxx]^12^ → [*Vxx*]^12^	Mikkola et al., 2012
																												**New**: Metanicin B: [Leu]^12^ → [*Vxx*]^12^	Kimonyo and Brückner, 2013
																												**New**: Hypophellin 3: [Lxx]^12^ → [*Vxx*]^12^	Röhrich et al., 2013
																												**New**: Pept-1951-c: [Lxx]12 → [*Vxx*]12	Tamandegani et al., 2016
																												**New**: Hyporientalin A: [Aib]12 → [*Vxx*]12	Touati et al., 2018
Pept-A-IIIb	1936	1959	991	1149	788	38.17	Ac	Aib	Ala	Aib	Ala	Aib	Ala	Gln	Aib	Vxx	*Ala*	Gly	Lxx	Aib	Pro	Vxx	Aib	Vxx	Gln	Gln	Pheol	**New**: Longibrachin A II: [Aib]^10^ → [Ala]^10^	Leclerc et al., 1998
																												**New**: Suzukacillin A 10a: [Aib]^10^ → [Ala]^10^	Krause et al., 2006b
																												**New**: Trichoaureocin 4: [Aib]^10^ → [Ala]^10^	Brückner et al., 2002
																												**New**: Trichoaureocin 1d: [Aib]^17^ → [Ala]^17^	Brückner et al., 2002
																												**New**: Trichobrachin II 07, 08, 09, IIb B: [Aib]^10^ → [Ala]^10^	Krause et al., 2007
																												**New**: Trichokonin VII: [Aib]^10^ → [Ala]^10^	Huang et al., 1996
																												**New**: Trilongin BII: [Aib]^10^ → [Ala]^10^	Mikkola et al., 2012
																												**New**: Metanicin B: [Aib]^10^ → [Ala]^10^	Kimonyo and Brückner, 2013
																												**New**: Hypophellin 3: [Aib]^10^ → [Ala]^10^	Röhrich et al., 2013
																												**New**: Pept-1951-c: [Aib]^10^ → [Ala]^10^	Tamandegani et al., 2016
Pept-A-IIIc	1936	1959	991	1149	788	39.89	Ac	Aib	Ala	Aib	Ala	Aib	Aib	Gln	Aib	*Vxx*	*Ala*	Gly	*Vxx*	Aib	Pro	Vxx	Aib	Vxx	Gln	Gln	Pheol	**New**: Pept-1965-c-1,−2: [Lxx]^9^ → [*Vxx*]^9^ and [Lxx]^12^ → [*Vxx*]^12^	Tamandegani et al., 2016
																												**New**: Hyporientalin A: [Aib]^10^ → [Ala]^10^	Touati et al., 2018
Pept-A-IVa	1936	1959	991	1163	774	40.21	Ac	Aib	Ala	Aib	Ala	Aib	Ala	Gln	Aib	Vxx	Aib	Gly	Lxx	Aib	Pro	Vxx	Aib	Aib	Gln	Gln	Pheol	Longibrachin A I (Positional isomer of Pept-A-VIIa)	Leclerc et al., 1998
																												Trichoaureocin 3	Brückner et al., 2002
																												Trichobrachin II 05, 06 IIb A	Krause et al., 2007
																												Trichokonin VI	Huang et al., 1994
																												Trilongin BI	Mikkola et al., 2012
																												Metanicin A	Kimonyo and Brückner, 2013
																												Gliodeliquescin A	Brückner and Przybylski, 1984
																												Hypophellin 1	Röhrich et al., 2013
																												Longibrachin A I, Trilongin BI	Tamandegani et al., 2016
Pept-A-IVb	1936	1959	991	1163	774	40.18	Ac	Aib	Ala	Aib	Ala	Aib	Ala	Gln	Aib	Lxx	*Ala*	Gly	Lxx	Aib	Pro	Vxx	Aib	Aib	Gln	Gln	Pheol	**New:** Suzukacillin A 11a, 09: [Aib]^10^ → [Ala]^10^	Krause et al., 2006b
																												**New:** Trichocellin-TC-A-V; -VII: [Aib]^10^ → [Ala]^10^	Wada et al., 1994
Pept-A-Va	1950	1973	998	1177	774	40.73	Ac	Aib	Ala	Aib	Ala	Aib	Aib	Gln	Aib	Vxx	Aib	Gly	Lxx	Aib	Pro	Vxx	Aib	Aib	Gln	Gln	Pheol	Trichosporin TS-B-IVc (Position isomer of Pept-A-XVIa)	Iida et al., 1990
																												Longibrachin A III	Leclerc et al., 1998
																												Trichoaureocin 5	Brückner et al., 2002
																												Trichobrachin IIb C	Krause et al., 2007
																												Trichokonin VIII	Huang et al., 1996
																												Trilongin BIII	Mikkola et al., 2012
																												Metanicin C	Kimonyo and Brückner, 2013
																												Hypophellin 5	Röhrich et al., 2013
																												Longibrachin A III.	Tamandegani et al., 2016
Pept-A-Vb	1950	1973	998	1177	774	41.40	Ac	Aib	Ala	Aib	Ala	Aib	Ala	Gln	Aib	Lxx	Aib	Gly	Lxx	Aib	Pro	Vxx	Aib	Aib	Gln	Gln	Pheol	Suzukacillin A 11a, A 09	Krause et al., 2006b.
																												Trichocellin TC-A-V, TC-A-VII	Wada et al., 1994
Pept-A-VIa	1937	1960	991.5	1163	775	41.46	Ac	Aib	Ala	Aib	Ala	Aib	Ala	Gln	Aib	Vxx	Aib	Gly	Lxx	Aib	Pro	Vxx	Aib	Aib	Glu	Gln	Pheol	Longibrachin B II	Leclerc et al., 1998
																												Trilongin CI	Mikkola et al., 2012
																												Hypophellin 2	Röhrich et al., 2013
																												Longibrachin B II., Trilongin CI.	Tamandegani et al., 2016
Pept-A-VIb	1937	1960	991.5	1163	775	41.50	Ac	Aib	Ala	Aib	Ala	Aib	Ala	Gln	Aib	*Lxx*	*Ala*	Gly	*Lxx*	Aib	Pro	Vxx	Aib	Aib	Glu	Gln	Pheol	**New**: Longibrachin B II: [Val]^9^ → [*Lxx*]^9^ and [Aib]^10^ → [*Ala*]^10^	Leclerc et al., 1998
																												**New**: Trilongin CI: [Vxx]^9^ → [*Lxx*]^9^ and [Aib]^10^ → [*Ala*]^10^	Mikkola et al., 2012
																												**New**: Hypophellin 2: [Vxx]^9^ → [*Lxx*]^9^ and [Aib]^10^ → [*Ala*]^10^	Röhrich et al., 2013
																												**New**: Longibrachin B II., Trilongin CI.: [Vxx]^9^ → [*Lxx*]^9^ and [Aib]^10^ → [*Ala*]^10^	Tamandegani et al., 2016
																												**New**: Trichocellin TC-B-I: [Aib]^10^ → [*Ala*]^10^ and [Aib]^12^ → [*Lxx*]^12^	Wada et al., 1994
Pept-A-VIIa	1936	1959	991	1163	774	41.00	Ac	Aib	Ala	Aib	Ala	Aib	Ala	Gln	Aib	Vxx	Aib	Gly	Lxx	Aib	Pro	Vxx	Aib	Aib	Gln	Gln	Pheol	(Positional isomer of Pept-A-IVa)	→ Pept-A-IVa
Pept-A-VIIb	1936	1959	991	1163	774	42.53	Ac	Aib	Ala	*Vxx*	Ala	Aib	Ala	Gln	Aib	Vxx	Ala	Gly	Lxx	Aib	Pro	Vxx	Aib	Aib	Gln	Gln	Pheol	**New**: Trichoaureocin 1d: [Aib]^3^ → [*Vxx*]^3^	Brückner et al., 2002
Pept-A-VIIIa	1950	1973	998	1177	774	42.29	Ac	Aib	Ala	*Vxx*	Ala	Aib	Ala	Gln	Aib	*Lxx*	*Ala*	Gly	Lxx	Aib	Pro	Vxx	Aib	Aib	Gln	Gln	Pheol	**New**: Suzukacillin A 11a, 09: [Aib]^3^ → [*Vxx*]^3^and [Aib]^10^ → [*Ala*]^10^	Krause et al., 2006b
																												**New**: Trichocellin-TC-A-V; -VII: [Aib]^3^ → [*Vxx*]^3^ and [Aib]^10^ → [*Ala*]^10^	Wada et al., 1994
																												**New**: Trichoaureocin 1d: [Aib]^3^ → [*Vxx*]^3^ and [Val]^9^ → [*Lxx*]^9^	(Brückner et al., 2002)
Pept-A-VIIIb	1950	1973	998	1177	774	42.46	Ac	Aib	Ala	*Vxx*	Ala	Aib	Ala	Gln	Aib	Vxx	Aib	Gly	Lxx	Aib	Pro	Vxx	Aib	Aib	Gln	Gln	Pheol	**New**: Longibrachin A I: [Aib]^3^ → [*Vxx*]^3^	Leclerc et al., 1998
																												**New**: Trichoaureocin 3: [Ala]^3^ → [*Vxx*]^3^	Brückner et al., 2002
																												**New**: Trichobrachin II 03: [Aib]^3^ → [*Vxx*]^3^	Krause et al., 2007
																												**New**: Trichobrachin II 05, 06 IIb A: [Aib]^3^ → [*Vxx*]^3^	Krause et al., 2007
																												**New**: Trichokonin IIc: [Ala]^3^ → [*Vxx*]^3^	Huang et al., 1996
																												**New**: Trichokonin VI: [Aib]^3^ → [*Vxx*]^3^	Huang et al., 1994
																												**New**: Trilongin BI: [Aib]^3^ → [*Vxx*]^3^	Mikkola et al., 2012
																												**New**: Metanicin A: [Aib]^3^ → [*Vxx*]^3^	Kimonyo and Brückner, 2013
																												**New**: Gliodeliquescin A: [Aib]^3^ → [*Vxx*]^3^	Brückner and Przybylski, 1984
																												**New**: Hypophellin 1: [Aib]^3^ → [*Vxx*]^3^	Röhrich et al., 2013
																												**New**: Longibrachin A I, Trilongin BI: [Aib]^3^ → [*Vxx*]^3^	Tamandegani et al., 2016
Pept-A-IXa	1950	1973	998	1163	788	42.76	Ac	Aib	Ala	Aib	Ala	Aib	Ala	Gln	Aib	Vxx	Aib	Gly	Lxx	Aib	Pro	Vxx	Aib	Vxx	Gln	Gln	Pheol	Longibrachin A II (Position isomer of Pept-A-XVa and Pept-A-XVIIb)	Leclerc et al., 1998
																												Suzukacillin A 10a	Krause et al., 2006b
																												Trichoaureocin 4	Brückner et al., 2002
																												Trichobrachin II 07, 08, 09, IIb B	Krause et al., 2007
																												Trichokonin VII	Huang et al., 1996
																												Trilongin BII	Mikkola et al., 2012
																												Metanicin B	Kimonyo and Brückner, 2013
																												Hypophellin 3	Röhrich et al., 2013
																												Pept-1951-c	Tamandegani et al., 2016
																												Hyporientalin A	Touati et al., 2018
Pept-A-IXb	1950	1973	998	1163	788	42.84	Ac	Aib	Ala	Aib	Ala	Aib	Ala	Gln	Aib	Lxx	*Ala*	Gly	Lxx	Aib	Pro	Vxx	Aib	Vxx	Gln	Gln	Pheol	**New**: Suzukacillin A 10b, 11b, 13: [Aib]^10^ → [*Ala*]^10^	Krause et al., 2006b
																												**New**: Trichocellin TC-A-VI, TC-A-VIII: [Aib]^10^ → [*Ala*]^10^	Wada et al., 1994
Pept-A-Xa	1964	1987	1005	1177	788	43.28	Ac	Aib	Ala	Aib	Ala	Aib	Aib	Gln	Aib	Vxx	Aib	Gly	Lxx	Aib	Pro	Vxx	Aib	Vxx	Gln	Gln	Pheol	Longibrachin A IV (Position isomer of Pept-A-XIVb Pept-A-XVIIa, Pept-A-XXIa, and Pept-XXVa)	Leclerc et al., 1998
																												Trichoaureocin 6	Brückner et al., 2002
																												Trichobrachin II 10, IIb D	Krause et al., 2007
																												Trichokonin IX	Huang et al., 1995
																												Trilongin BIV	Mikkola et al., 2012
																												Metanicin D	Kimonyo and Brückner, 2013
																												Hypophellin 7	Röhrich et al., 2013
Pept-A-Xb	1964	1987	1005	1177	788	42.89	Ac	Aib	Ala	Aib	Ala	Aib	Ala	Gln	Aib	Lxx	Aib	Gly	Lxx	Aib	Pro	Vxx	Aib	Vxx	Gln	Gln	Pheol	Suzukacillin A 10b, 11b, 13	Krause et al., 2006b
																												Trichocellin TC-A-VI, TC-A-VIII	Wada et al., 1994
Pept-A-XIa	1951	1974	998.5	1177	775	43.60	Ac	Aib	Ala	*Vxx*	Ala	Aib	Ala	Gln	Aib	Vxx	Aib	Gly	Lxx	Aib	Pro	Vxx	Aib	Aib	Glu	Gln	Pheol	**New**: Longibrachin B II: [Aib]^3^ → [*Vxx*]^3^	Leclerc et al., 1998
																												**New**: Trilongin CI: [Aib]^3^ → [*Vxx*]^3^	Mikkola et al., 2012
																												**New**: Hypophellin 2: [Aib]^3^ → [*Vxx*]^3^	Röhrich et al., 2013
																												**New**: Longibrachin B II., Trilongin CI.: [Aib]^3^ → [*Vxx*]^3^	Tamandegani et al., 2016
Pept-A-XIb	1951	1974	998.5	1177	775	43.60	Ac	Aib	Ala	Aib	Ala	Aib	Aib	Gln	Aib	Vxx	Aib	Gly	Lxx	Aib	Pro	Vxx	Aib	Aib	Glu	Gln	Pheol	Trilongin CIII (Positional isomer of Pept-A-XIXa)	Mikkola et al., 2012
																												Hypophellin 6	Röhrich et al., 2013
																												Longibrachin B III., Trilongin CIII.	Tamandegani et al., 2016
Pept-A-XIc	1951	1974	998.5	1177	775	43.62	Ac	Aib	Ala	*Vxx*	Ala	Aib	Ala	Gln	Aib	*Lxx*	*Ala*	Gly	*Lxx*	Aib	Pro	Vxx	Aib	Aib	Glu	Gln	Pheol	**New**: Longibrachin B II: [Aib]^3^ → [*Vxx*]^3^, [Val]^9^ → [*Lxx*]^9^, and [Aib]^10^ → [*Ala*]^10^	Leclerc et al., 1998
																												**New**: Trilongin CI: [Aib]^3^ → [*Vxx*]^3^, [Vxx]^9^ → [*Lxx*]^9^, and [Aib]^10^ → [*Ala*]^10^	Mikkola et al., 2012
																												**New**: Hypophellin 2: [Aib]^3^ → [*Vxx*]^3^, [Vxx]^9^ → [*Lxx*]^9^, and [Aib]^10^ → [*Ala*]^10^	Röhrich et al., 2013
																												**New**: Longibrachin B II., Trilongin CI.: [Aib]^3^ → [*Vxx*]^3^, [Vxx]^9^ → [*Lxx*]^9^, and [Aib]^10^ → [*Ala*]^10^	Tamandegani et al., 2016
																												**New**: Trichocellin TC-B-I: [Aib]^3^ → [*Vxx*]^3^, [Aib]^10^ → [*Ala*]^10^, and [Aib]^12^ → [*Lxx*]^12^	Wada et al., 1994
Pept-A-XII	1937	1960	991.5	1163	775	42.81	Ac	Aib	Ala	Aib	Ala	Aib	Ala	Gln	Aib	*Lxx*	*Ala*	Gly	Lxx	Aib	Pro	Vxx	Aib	Aib	Glu	Gln	Pheol	(Positional isomer of Pept-A-VIb)	→ Pept-A-VIb
Pept-A-XIIIa	1951	1974	998.5	1163	789	44.14	Ac	Aib	Ala	Aib	Ala	Aib	Ala	Gln	Aib	Vxx	Aib	Gly	Lxx	Aib	Pro	Vxx	Aib	Vxx	Glu	Gln	Pheol	Longibrachin B III	Leclerc et al., 1998
																												Trilongin CII	Mikkola et al., 2012
																												Hypophellin 4	Röhrich et al., 2013
																												Pept-1952-d	Tamandegani et al., 2016
																												Longibrachin A II., Trilongin BII.	Tamandegani et al., 2016
Pept-A-XIIIb	1951	1974	998.5	1163	789	44.16	Ac	Aib	Ala	Aib	Ala	Aib	Ala	Gln	Aib	*Lxx*	*Ala*	Gly	*Lxx*	Aib	Pro	Vxx	Aib	Vxx	Glu	Gln	Pheol	**New**: Longibrachin B III: [Val]^9^ → [*Lxx*]^9^ and [Aib]^10^ → [*Ala*]^10^	Leclerc et al., 1998
																												**New**: Trilongin CII: [Vxx]^9^ → [*Lxx*]^9^ and [Aib]^10^ → [*Ala*]^10^	Mikkola et al., 2012
																												**New**: Hypophellin 4: [Vxx]^9^ → [*Lxx*]^9^ and [Aib]^10^ → [*Ala*]^10^	Röhrich et al., 2013
																												**New**: Pept-1952-d: [Vxx]^9^ → [*Lxx*]^9^ and [Aib]^10^ → [*Ala*]^10^	Tamandegani et al., 2016
																												**New**: Longibrachin B II., Trilongin CI.: [Vxx]^9^ → [*Lxx*]^9^ and [Aib]^10^ → [*Ala*]^10^	Tamandegani et al., 2016
																												**New**: Trichocellin TC-B-II: [Aib]^10^ → [*Ala*]^10^ and [Aib]^12^ → [*Lxx*]^12^	Wada et al., 1994
Pept-A-XIVa	1965	1988	1005.5	1177	789	44.22	Ac	Aib	Ala	Aib	Ala	Aib	Aib	Gln	Aib	Lxx	Ala	Gly	Lxx	Aib	Pro	Vxx	Aib	Vxx	Glu	Gln	Pheol	Pept-1966-d	Tamandegani et al., 2016
Pept-A-XIVb	1964	1987	1005	1177	788	44.13	Ac	Aib	Ala	Aib	Ala	Aib	Aib	Gln	Aib	Vxx	Aib	Gly	Lxx	Aib	Pro	Vxx	Aib	Vxx	Gln	Gln	Pheol	(Position isomer of Pept-A-Xa, Pept-A-XVIIa, Pept-A-XXIa, and Pept-XXVa)	→ Pept-A-Xa
Pept-A-XVa	1950	1973	998	1163	788	45.00	Ac	Aib	Ala	Aib	Ala	Aib	Ala	Gln	Aib	Vxx	Aib	Gly	Lxx	Aib	Pro	Vxx	Aib	Vxx	Gln	Gln	Pheol	(Position isomer of Pept-A-IXa and Pept-A-XVIIb)	→ Pept-A-IXa
Pept-A-XVb	1964	1987	1005	1177	788	44.74	Ac	Aib	Ala	Aib	Ala	Aib	Aib	Gln	Aib	Lxx	*Ala*	Gly	Lxx	Aib	Pro	Vxx	Aib	Vxx	Gln	Gln	Pheol	Pept-1965-c-1, c-2 (Position isomer of Pept-A-XXIb)	Tamandegani et al., 2016
Pept-A-XVIa	1950	1973	998	1177	774	45.21	Ac	Aib	Ala	Aib	Ala	Aib	Aib	Gln	Aib	Vxx	Aib	Gly	Lxx	Aib	Pro	Vxx	Aib	Aib	Gln	Gln	Pheol	(Position isomer of Pept-A-Va)	→ Pept-A-Va
Pept-A-XVIb	1950	1973	998	1177	774	45.33	Ac	Aib	Ala	Aib	Ala	Aib	Aib	Gln	Aib	Lxx	*Ala*	Gly	Lxx	Aib	Pro	Vxx	Aib	*Aib*	Gln	Gln	Pheol	**New**: Trichosporin TS-B-VIa: [Aib]^10^ → [*Ala*]^10^	Iida et al., 1990
																												**New**: *Trichoderma citrinoviride* sequence 7: [Aib]^10^ → [*Ala*]^10^	Maddau et al., 2009
																												**New**: Pept-1965-c-1, c-2: [Vxx]^17^ → [*Aib*]^17^	Tamandegani et al., 2016
Pept-A-XVIIa	1964	1987	1005	1177	788	46.21	Ac	Aib	Ala	Aib	Ala	Aib	Aib	Gln	Aib	Vxx	Aib	Gly	Lxx	Aib	Pro	Vxx	Aib	Vxx	Gln	Gln	Pheol	(Position isomer of Pept-A-Xa, Pept-A-XIVb, Pept-A-XXIa, and Pept-XXVa)	→ Pept-A-Xa
Pept-A-XVIIb	1950	1973	998	1163	788	46.18	Ac	Aib	Ala	Aib	Ala	Aib	Ala	Gln	Aib	Vxx	Aib	Gly	Lxx	Aib	Pro	Vxx	Aib	Vxx	Gln	Gln	Pheol	(Position isomer of Pept-A-XVa and Pept-A-IXa)	→ Pept-A-IXa
Pept-A-XVIII	1978	2001	1012	1191	788	46.36	Ac	Aib	Ala	*Vxx*	Ala	Aib	Aib	Gln	Aib	Vxx	Aib	Gly	Lxx	Aib	Pro	Vxx	Aib	Vxx	Gln	Gln	Pheol	**New**: Trichosporin TS-B-IVd: [Ala]^3^ → [*Vxx*]^3^ (Position isomer of Pept-A-XXIV and Pept-XXVI)	Iida et al., 1990
																												**New**: Longibrachin A IV: [Aib]^3^ → [*Vxx*]^3^	Leclerc et al., 1998
																												**New**: Trichoaureocin 6: [Aib]^3^ → [*Vxx*]^3^	Brückner et al., 2002
																												**New**: Trichobrachin II 10, IIb D: [Aib]^3^ → [*Vxx*]^3^	Krause et al., 2007
																												**New**: Trichokonin IX: [Aib]^3^ → [*Vxx*]^3^	Huang et al., 1995
																												**New**: Trilongin BIV: [Aib]^3^ → [*Vxx*]^3^	Mikkola et al., 2012
																												**New**: Metanicin D: [Aib]^3^ → [*Vxx*]^3^	Kimonyo and Brückner, 2013
																												**New**: Hypophellin 7: [Aib]^3^ → [*Vxx*]^3^	Röhrich et al., 2013
Pept-A-XIXa	1951	1974	998.5	1177	775	46.67	Ac	Aib	Ala	Aib	Ala	Aib	Aib	Gln	Aib	Vxx	Aib	Gly	Lxx	Aib	Pro	Vxx	Aib	Aib	Glu	Gln	Pheol	(Positional isomer of Pept-A-XIb)	→ Pept-A-XIb
Pept-A-XIXb	1951	1974	998.5	1177	775	46.86	Ac	Aib	Ala	Aib	Ala	Aib	Aib	Gln	Aib	Lxx	*Ala*	Gly	Lxx	Aib	Pro	Vxx	Aib	*Aib*	*Glu*	Gln	Pheol	**New:** Trichosporin TS-B-VIa: [Aib]^10^ → [*Ala*]^10^ and [Gln]^18^ → [*Glu*]^18^	Iida et al., 1990
																												**New:** *Trichoderma citrinoviride* sequence 7, [Aib]^10^ → [*Ala*]^10^ and [Gln]^18^ → [*Glu*]^18^	Maddau et al., 2009
																												**New**: Pept-1965-c-1, c-2: [Vxx]^17^ → [*Aib*]^17^ and [Gln]^18^ → [*Glu*]^18^	Tamandegani et al., 2016
Pept-A-XX	1964	1987	1005	1191	774	47.30	Ac	Aib	Ala	*Vxx*	Ala	Aib	Aib	Gln	Aib	Vxx	Aib	Gly	Lxx	Aib	Pro	Vxx	Aib	Aib	Gln	Gln	Pheol	**New**: Trichosporin TS-B-IVc: [Aib]^3^ → [*Vxx*]^3^ (Position isomer of Pept-A-XXIIa)	Iida et al., 1990
																												**New**: Longibrachin A III: [Aib]^3^ → [*Vxx*]^3^	Leclerc et al., 1998
																												**New**: Trichoaureocin 5: [Aib]^3^ → [*Vxx*]^3^	Brückner et al., 2002
																												**New**: Trichobrachin, IIb C: [Aib]^3^ → [*Vxx*]^3^	Krause et al., 2007
																												**New**: Trichokonin VIII: [Aib]^3^ → [*Vxx*]^3^	Huang et al., 1996
																												**New**: Trichosporin TS-B-IIId: [Ala]^3^ → [*Vxx*]^3^	Iida et al., 1990
																												**New**: Trilongin BIII: [Aib]^3^ → [*Vxx*]^3^	Mikkola et al., 2012
																												**New**: Metanicin C: [Aib]^3^ → [*Vxx*]^3^	Kimonyo and Brückner, 2013
																												**New**: Hypophellin 5: [Aib]^3^ → [*Vxx*]^3^	Röhrich et al., 2013
																												**New**: Longibrachin A III.: [Aib]^3^ → [*Vxx*]^3^	Tamandegani et al., 2016
Pept-A-XXIa	1964	1987	1005	1177	788	47.85	Ac	Aib	Ala	Aib	Ala	Aib	Aib	Gln	Aib	Vxx	Aib	Gly	Lxx	Aib	Pro	Vxx	Aib	Vxx	Gln	Gln	Pheol	(Position isomer of Pept-A-Xa, Pept-A-XIVb, Pept-A-XVIIa, and Pept-XXVa)	→ Pept-A-Xa
Pept-A-XXIb	1964	1987	1005	1177	788	47.75	Ac	Aib	Ala	Aib	Ala	Aib	Aib	Gln	Aib	Lxx	Ala	Gly	Lxx	Aib	Pro	Vxx	Aib	Vxx	Gln	Gln	Pheol	(Position isomer of Pept-A-XVb)	→ Pept-A-XVb
Pept-A-XXIIa	1964	1987	1005	1191	774	48.93	Ac	Aib	Ala	*Vxx*	Ala	Aib	Aib	Gln	Aib	Vxx	Aib	Gly	Lxx	Aib	Pro	Vxx	Aib	Aib	Gln	Gln	Pheol	**New**: (Position isomer of Pept-A-XX)	→ Pept-A-XX
Pept-A-XXIIb	1964	1987	1005	1191	774	48.79	Ac	Aib	Ala	Aib	Ala	Aib	Aib	Gln	Aib	Lxx	Aib	Gly	Lxx	Aib	Pro	Vxx	Aib	Aib	Gln	Gln	Pheol	Trichosporin TS-B-VIa	Iida et al., 1990
																												*Trichoderma citrinoviride* sequence 7	Maddau et al., 2009
Pept-A-XXIII	1965	1988	1005.5	1177	789	49.13	Ac	Aib	Ala	Aib	Ala	Aib	Aib	Gln	Aib	Vxx	Aib	Gly	Lxx	Aib	Pro	Vxx	Aib	Vxx	Glu	Gln	Pheol	Trilongin CIV (Positional isomer of Pept-A-XXVIIa)	Mikkola et al., 2012
																												Hypophellin 8	Röhrich et al., 2013
Pept-A-XXIV	1978	2001	1012	1191	788	49.89	Ac	Aib	Ala	*Vxx*	Ala	Aib	Aib	Gln	Aib	Vxx	Aib	Gly	Lxx	Aib	Pro	Vxx	Aib	Vxx	Gln	Gln	Pheol	(Position isomer of Pept-A-XVIII and Pept-XXVI)	→ Pept-A-XVIII
Pept-A-XXVa	1964	1987	1005	1177	788	49.65	Ac	Aib	Ala	Aib	Ala	Aib	Aib	Gln	Aib	Vxx	Aib	Gly	Lxx	Aib	Pro	Vxx	Aib	Vxx	Gln	Gln	Pheol	(Position isomer of Pept-A-Xa, Pept-A-XIVb, Pept-A-XVIIa, and Pept-XXIa)	→ Pept-A-Xa
Pept-A-XXVb	1978	2001	1012	1191	788	49.72	Ac	Aib	Ala	Aib	Ala	Aib	Aib	Gln	Aib	Lxx	Aib	Gly	Lxx	Aib	Pro	Vxx	Aib	Vxx	Gln	Gln	Pheol	Suzukacillin A 12 (Position isomer of Pept-A-XXVIb and Pept-XXVIIb)	Krause et al., 2006b
Pept-A-XXVIa	1978	2001	1012	1191	788	51.29	Ac	Aib	Ala	*Vxx*	Ala	Aib	Aib	Gln	Aib	Vxx	Aib	Gly	Lxx	Aib	Pro	Vxx	Aib	Vxx	Gln	Gln	Pheol	(Position isomer of Pept-A-XVIII and Pept-XXIV)	→ Pept-A-XVIII
Pept-A-XXVIb	1978	2001	1012	1191	788	50.85	Ac	Aib	Ala	Aib	Ala	Aib	Aib	Gln	Aib	Lxx	Aib	Gly	Lxx	Aib	Pro	Vxx	Aib	Vxx	Gln	Gln	Pheol	(Position isomer of Pept-A-XXVb and Pept-XXVIIb)	→ Pept-A-XVb
Pept-A-XXVIIa	1965	1988	1005.5	1177	789	51.44	Ac	Aib	Ala	Aib	Ala	Aib	Aib	Gln	Aib	Vxx	Aib	Gly	Lxx	Aib	Pro	Vxx	Aib	Vxx	Glu	Gln	Pheol	(Positional isomer of Pept-A-XXIII)	→ Pept-A-XXIII
Pept-A-XXVIIb	1978	2001	1012	1191	788	51.59	Ac	Aib	Ala	Aib	Ala	Aib	Aib	Gln	Aib	Lxx	Aib	Gly	Lxx	Aib	Pro	Vxx	Aib	Vxx	Gln	Gln	Pheol	(Position isomer of Pept-A-XXVb and Pept-XXVIb)	→ Pept-A-XVb

*Variable residues are UNDERLINED in the table header; minor sequence variants are UNDERLINED in the sequences. Amino acid exchanges in new compounds are set in italic*.

**Table 3:** Sequences of the newly identified group B peptaibol compounds from *Trichoderma* species of the Longibrachiatum Clade and their similarities to known peptaibols available in the “Comprehensive Peptaibiotics Database.”

**Peptide**	**M**	**[M+Na]^**+**^**	**[M+2Na]^**2+**^**	**b_**13**_**	**y_**7**_**	**rt-GK (min)**	**R**	**R1**	**R2**	**R3**	**R4**	**R5**	**R6**	**R7**	**R8**	**R9**	**R10**	**R11**	**R12**	**R13**	**R14**	**R15**	**R16**	**R17**	**R18**	**R19**	**R20**	**Compound identical or positionally isomeric with**	**References**
Pept-B-I	1908	1931	977	1135	774	22.59	Ac	Aib	Ala	*Ala*	Ala	Aib	Ala	Gln	Aib	Lxx	Aib	Gly	Aib	Aib	Pro	Vxx	Aib	Aib	Gln	Gln	Pheol	**New:** Paracelsin B: [Aib]^3^ → [*Ala*]^3^ (Positional isomer of Pept-B-II, III, and V)	Pócsfalvi et al., 1997
																												**New:** Saturnisporin SA I: [Aib]^3^ → [*Ala*]^3^	Rebuffat et al., 1993
																												**New:** Suzukacillin A 02, A 06: [Aib]^3^ → [*Ala*]^3^	Krause et al., 2006b
																												**New:** Trichocellin TC-A-I, TC-A-III: [Aib]^3^ → [*Ala*]^3^	Wada et al., 1994
Pept-B-II	1908	1931	977	1135	774	24.79	Ac	Aib	Ala	*Ala*	Ala	Aib	Ala	Gln	Aib	Lxx	Aib	Gly	Aib	Aib	Pro	Vxx	Aib	Aib	Gln	Gln	Pheol	**New:** (Positional isomer of Pept-B-I, III, and V)	→ Pept-B-I
Pept-B-III	1908	1931	977	1135	774	25.62	Ac	Aib	Ala	*Ala*	Ala	Aib	Ala	Gln	Aib	Lxx	Aib	Gly	Aib	Aib	Pro	Vxx	Aib	Aib	Gln	Gln	Pheol	**New:** (Positional isomer of Pept-B-I, II, and V)	→ Pept-B-I
Pept-B-IV	1922	1945	984	1135	788	25.72	Ac	Aib	Ala	*Ala*	Ala	Aib	Ala	Gln	Aib	Lxx	Aib	Gly	Aib	Aib	Pro	Vxx	Aib	Vxx	Gln	Gln	Pheol	**New:** Paracelsin H: [Aib]^3^ → [*Ala*]^3^ (Positional isomer of Pept-B-VII)	Pócsfalvi et al., 1997
																												**New:** Saturnisporin SA II: [Aib]^3^ → [*Ala*]^3^	Rebuffat et al., 1993
																												**New:** Suzukacillin A 04, A 08: [Aib]^3^ → [*Ala*]^3^	Krause et al., 2006b
																												**New:** Trichocellin TC-A-II, TC-A-IV: [Aib]^3^ → [*Ala*]^3^	Wada et al., 1994
Pept-B-V	1908	1931	977	1135	774	26.35	Ac	Aib	Ala	*Ala*	Ala	Aib	Ala	Gln	Aib	Lxx	Aib	Gly	Aib	Aib	Pro	Vxx	Aib	Aib	Gln	Gln	Pheol	**New:** (Positional isomer of Pept-B-I, II, and III)	→ Pept-B-I
Pept-B-VI	1922	1945	984	1149	774	27.22	Ac	Aib	Ala	Aib	Ala	Aib	Ala	Gln	Aib	Lxx	Aib	Gly	Aib	Aib	Pro	Vxx	Aib	Aib	Gln	Gln	Pheol	Paracelsin B (Positional isomer of Pept-B-XII, XVIII, and XXIII)	Pócsfalvi et al., 1997
																												Saturnisporin SA I	Rebuffat et al., 1993
																												Suzukacillin A 02, A 06	Krause et al., 2006b
																												Trichocellin TC-A-I, TC-A-III	Wada et al., 1994
Pept-B-VII	1922	1945	984	1135	788	27.80	Ac	Aib	Ala	*Ala*	Ala	Aib	Ala	Gln	Aib	Lxx	Aib	Gly	Aib	Aib	Pro	Vxx	Aib	Vxx	Gln	Gln	Pheol	**New:** (Positional isomer of Pept-B-IV)	→ Pept-B-IV
Pept-B-VIII	1936	1959	991	1149	788	27.27	Ac	Aib	Ala	Aib	Ala	Aib	Ala	Gln	Aib	Lxx	Aib	Gly	Aib	Aib	Pro	Vxx	Aib	Vxx	Gln	Gln	Pheol	Paracelsin H (Positional isomer of Pept-B-XVII, XIX, XXII, and XXIXb)	Pócsfalvi et al., 1997
																												Saturnisporin SA II	Rebuffat et al., 1993
																												Suzukacillin A 04, A 08	Krause et al., 2006b
																												Trichocellin TC-A-II, TC-A-IV	Wada et al., 1994
Pept-B-IXa	1908	1931	977	1135	774	28.44	Ac	Aib	Ala	Aib	Ala	Aib	Ala	Gln	Aib	Vxx	Aib	Gly	Aib	Aib	Pro	Vxx	Aib	Aib	Gln	Gln	Pheol	Paracelsin A	Pócsfalvi et al., 1997
																												Suzukacillin A 01	Krause et al., 2006b
Pept-B-IXb	1908	1931	977	1135	774	28.38	Ac	Aib	Ala	Aib	Ala	Aib	Ala	Gln	Aib	Lxx	Aib	Gly	Ala	Aib	Pro	Vxx	Aib	*Aib*	Gln	Gln	Pheol	**New:** *Trichoderma citrinoviride* sequence 1: [Vxx]^17^ → [*Aib*]^17^	Maddau et al., 2009
Pept-B-X	1922	1945	984	1135	788	28.77	Ac	Aib	Ala	Aib	Ala	Aib	Ala	Gln	Aib	Vxx	Aib	Gly	Aib	Aib	Pro	Vxx	Aib	Vxx	Gln	Gln	Pheol	Paracelsin F (Positional isomer of Pept-B-XI, XIII, and XVa)	Pócsfalvi et al., 1997
																												Suzukacillin A 03	Krause et al., 2006b
Pept-B-XI	1922	1945	984	1135	788	29.25	Ac	Aib	Ala	Aib	Ala	Aib	Ala	Gln	Aib	Vxx	Aib	Gly	Aib	Aib	Pro	Vxx	Aib	Vxx	Gln	Gln	Pheol	(Positional isomer of Pept-B-X, XIII, and XVa)	→ Pept-B-X
Pept-B-XII	1922	1945	984	1149	774	29.90	Ac	Aib	Ala	Aib	Ala	Aib	Ala	Gln	Aib	Lxx	Aib	Gly	Aib	Aib	Pro	Vxx	Aib	Aib	Gln	Gln	Pheol	(Positional isomer of Pept-B-VI, XVIII, and XXIII)	→ Pept-B-VI
Pept-B-XIII	1922	1945	984	1135	788	30.28	Ac	Aib	Ala	Aib	Ala	Aib	Ala	Gln	Aib	Vxx	Aib	Gly	Aib	Aib	Pro	Vxx	Aib	Vxx	Gln	Gln	Pheol	(Positional isomer of Pept-B-X, XI, and XVa)	→ Pept-B-X
Pept-B-XIVa	1923	1946	984.5	1149	775	31.36	Ac	Aib	Ala	Aib	Ala	Aib	Ala	Gln	Aib	Lxx	Aib	Gly	Aib	Aib	Pro	Vxx	Aib	Aib	Glu	Gln	Pheol	Trichocellin TC-B-I	Wada et al., 1994
Pept-B-XIVb	1923	1946	984.5	1149	775	31.40	Ac	Aib	Ala	Aib	Ala	Aib	*Aib*	Gln	Aib	*Vxx*	Aib	Gly	Aib	Aib	Pro	Vxx	Aib	Aib	Glu	Gln	Pheol	**New:** Trichocellin TC-B-I: [Ala]^6^ → [*Aib*]^6^ and [Leu]^9^ → [*Vxx*]^9^	Wada et al., 1994
Pept-B-XVa	1922	1945	984	1135	788	31.48	Ac	Aib	Ala	Aib	Ala	Aib	Ala	Gln	Aib	Vxx	Aib	Gly	Aib	Aib	Pro	Vxx	Aib	Vxx	Gln	Gln	Pheol	(Positional isomer of Pept-B-X, XI, and -XIII)	→ Pept-B-X
Pept-B-XVb	1922	1945	984	1135	788	31.53	Ac	Aib	Ala	Aib	Ala	Aib	Ala	Gln	Aib	Lxx	Aib	Gly	Ala	Aib	Pro	Vxx	Aib	Vxx	Gln	Gln	Pheol	*Trichoderma citrinoviride* sequence 1	Maddau et al., 2009
Pept-B-XVI	1922	1945	984	1149	774	31.98	Ac	Aib	Ala	Aib	Ala	Aib	Aib	Gln	Aib	Vxx	Aib	Gly	Aib	Aib	Pro	Vxx	Aib	Aib	Gln	Gln	Pheol	Paracelsin C	Pócsfalvi et al., 1997
Pept-B-XVII	1936	1959	991	1149	788	32.67	Ac	Aib	Ala	Aib	Ala	Aib	Ala	Gln	Aib	Lxx	Aib	Gly	Aib	Aib	Pro	Vxx	Aib	Vxx	Gln	Gln	Pheol	(Positional isomer of Pept-B-VIII, XIX, XXII, and XXIXb)	→ Pept-B-VIII
Pept-B-XVIII	1922	1945	984	1149	774	33.49	Ac	Aib	Ala	Aib	Ala	Aib	Ala	Gln	Aib	Lxx	Aib	Gly	Aib	Aib	Pro	Vxx	Aib	Aib	Gln	Gln	Pheol	(Positional isomer of Pept-B-VI, XII, and XXIII)	→ Pept-B-VI
Pept-B-XIX	1936	1959	991	1149	788	33.55	Ac	Aib	Ala	Aib	Ala	Aib	Ala	Gln	Aib	Lxx	Aib	Gly	Aib	Aib	Pro	Vxx	Aib	Vxx	Gln	Gln	Pheol	(Positional isomer of Pept-B-VIII, XVII, XXII, and XXIXb)	→ Pept-B-VIII
Pept-B-XX	1936	1959	991	1163	774	34.41	Ac	Aib	Ala	Aib	Ala	Aib	Aib	Gln	Aib	Lxx	Aib	Gly	Aib	Aib	Pro	Vxx	Aib	Aib	Gln	Gln	Pheol	Paracelsin D (Positional isomer of Pept-B-XXXIIIa, XXXVa, XLIIb, XLVIa, and LVIII)	Pócsfalvi et al., 1997
																												Saturnisporin SA III	Rebuffat et al., 1993
																												Suzukacillin A 05	Krause et al., 2006b
Pept-B-XXI	1937	1960	991.5	1149	789	34.15	Ac	Aib	Ala	Aib	Ala	Aib	Ala	Gln	Aib	Lxx	Aib	Gly	Aib	Aib	Pro	Vxx	Aib	Vxx	Glu	Gln	Pheol	Trichocellin TC-B-II	Wada et al., 1994
Pept-B-XXII	1936	1959	991	1149	788	34.59	Ac	Aib	Ala	Aib	Ala	Aib	Ala	Gln	Aib	Lxx	Aib	Gly	Aib	Aib	Pro	Vxx	Aib	Vxx	Gln	Gln	Pheol	(Positional isomer of Pept-B-VIII, XVII, XIX, and XXIXb)	→ Pept-B-VIII
Pept-B-XXIII	1922	1945	984	1149	774	35.25	Ac	Aib	Ala	Aib	Ala	Aib	Ala	Gln	Aib	Lxx	Aib	Gly	Aib	Aib	Pro	Vxx	Aib	Aib	Gln	Gln	Pheol	(Positional isomer of Pept-B-VI, XII, and XVIII)	→ Pept-B-VI
Pept-B-XXIV	1950	1973	998	1163	788	35.59	Ac	Aib	Ala	Aib	Ala	Aib	Aib	Gln	Aib	Lxx	Aib	Gly	Aib	Aib	Pro	Vxx	Aib	Vxx	Gln	Gln	Pheol	Saturnisporin SA IV (Positional isomer of Pept-B-XXVII, XXXIIa, XXXVIII, and XLVa)	Rebuffat et al., 1993
																												Suzukacillin A 07	Krause et al., 2006b
Pept-B-XXV	1937	1960	991.5	1163	775	35.97	Ac	Aib	Ala	Aib	Ala	Aib	*Aib*	Gln	Aib	Lxx	Aib	Gly	Aib	Aib	Pro	Vxx	Aib	Aib	*Glu*	Gln	Pheol	**New:** Trichocellin TC-B-I: [Ala]^6^ → [*Aib*]^6^ (Positional isomer of Pept-B-XXXVII)	Wada et al., 1994
																												**New:** Paracelsin D: [Gln]^18^ → [*Glu*]^18^	Pócsfalvi et al., 1997
																												**New:** Saturnisporin SA III: [Gln]^18^ → [*Glu*]^18^	Rebuffat et al., 1993
																												**New:** Suzukacillin A 05: [Gln]^18^ → [*Glu*]^18^	Krause et al., 2006b
Pept-B-XXVI	1950	1973	998	1177	774	36.65	Ac	Aib	Ala	*Vxx*	Ala	Aib	Aib	Gln	Aib	Lxx	Aib	Gly	Aib	Aib	Pro	Vxx	Aib	Aib	Gln	Gln	Pheol	**New:** Paracelsin D: [Aib]^3^ → [*Vxx*]^3^	Pócsfalvi et al., 1997
																												**New:** Saturnisporin SA III: [Aib]^3^ → [*Vxx*]^3^	Rebuffat et al., 1993
																												**New:** Suzukacillin A 05: [Aib]^3^ → [*Vxx*]^3^	Krause et al., 2006b
Pept-B-XXVII	1950	1973	998	1163	788	37.31	Ac	Aib	Ala	Aib	Ala	Aib	Aib	Gln	Aib	Lxx	Aib	Gly	Aib	Aib	Pro	Vxx	Aib	Vxx	Gln	Gln	Pheol	(Positional isomer of Pept-B-XXIV, XXXIIa, XXXVIII, and XLVa)	→ Pept-B-XXIV
Pept-B-XXVIII	1950	1973	998	1177	774	37.89	Ac	Aib	Ala	Aib	Ala	*Vxx*	Aib	Gln	Aib	Lxx	Aib	Gly	Aib	Aib	Pro	Vxx	Aib	Aib	Gln	Gln	Pheol	**New:** Paracelsin D: [Aib]^5^ → [*Vxx*]^5^	Pócsfalvi et al., 1997
																												**New:** Saturnisporin SA III: [Aib]^5^ → [*Vxx*]^5^	Rebuffat et al., 1993
																												**New:** Suzukacillin A 05: [Aib]^5^ → [*Vxx*]^5^	Krause et al., 2006b
Pept-B-XXIXa	1936	1959	991	1149	788	38.30	Ac	Aib	Ala	Aib	Ala	Aib	Aib	Gln	Aib	Lxx	*Ala*	Gly	Aib	Aib	Pro	Vxx	Aib	Vxx	Gln	Gln	Pheol	**New:** Paracelsin D: [Aib]^10^ → [*Ala*]^10^	Pócsfalvi et al., 1997
																												**New:** Saturnisporin SA III: [Aib]^10^ → [*Ala*]^10^	Rebuffat et al., 1993
																												**New:** Suzukacillin A 05: [Aib]^10^ → [*Ala*]^10^	Krause et al., 2006b
Pept-B-XXIXb	1936	1959	991	1149	788	37.80	Ac	Aib	Ala	Aib	Ala	Aib	Ala	Gln	Aib	Lxx	Aib	Gly	Aib	Aib	Pro	Vxx	Aib	Vxx	Gln	Gln	Pheol	(Positional isomer of Pept-B-VIII, XVII, XIX, and XXII)	→ Pept-B-VIII
Pept-B-XXX	1950	1973	998	1177	774	38.51	Ac	Aib	Ala	Aib	Ala	Aib	Aib	Gln	Aib	Lxx	Aib	Gly	Vxx	Aib	Pro	Vxx	Aib	Aib	Gln	Gln	Pheol	*Trichoderma citrinoviride* sequence 4 (Positional isomer of Pept-B-XXXIIIc, XLIIa, XLVIb, and LIII)	Maddau et al., 2009
Pept-B-XXXI	1951	1974	998.5	1163	789	39.13	Ac	Aib	Ala	Aib	Ala	Aib	*Aib*	Gln	Aib	Lxx	Aib	Gly	Aib	Aib	Pro	Vxx	Aib	Vxx	*Glu*	Gln	Pheol	**New:** Trichocellin TC-B-II: [Ala]^6^ → [*Aib*]^6^ (Positional isomer of Pept-B-XXXIVb and LII)	Wada et al., 1994
																												**New:** Saturnisporin SA IV: [Gln]^18^ → [*Glu*]^18^	Rebuffat et al., 1993
																												**New:** Suzukacillin A 07: [Gln]^18^ → [*Glu*]^18^	Krause et al., 2006b
Pept-B-XXXIIa	1950	1973	998	1163	788	39.15	Ac	Aib	Ala	Aib	Ala	Aib	Aib	Gln	Aib	Lxx	Aib	Gly	Aib	Aib	Pro	Vxx	Aib	Vxx	Gln	Gln	Pheol	(Positional isomer of Pept-B-XXIV, XXVII, XXXVIII, and XLVa)	→ Pept-B-XXIV
Pept-B-XXXIIb	1964	1987	1005	1177	788	39.20	Ac	Aib	Ala	Aib	Ala	Aib	*Vxx*	Gln	Aib	Lxx	Aib	Gly	Aib	Aib	Pro	Vxx	Aib	Vxx	Gln	Gln	Pheol	**New:** Paracelsin H: [Ala]^6^ → [Vxx]^6^ (Positional isomer of Pept-B-XLIb)	Pócsfalvi et al., 1997
																												**New:** Saturnisporin SA II: [Ala]^6^ → [Vxx]^6^	Rebuffat et al., 1993
																												**New:** Saturnisporin SA IV: [Aib]^6^ → [Vxx]^6^	Rebuffat et al., 1993
																												**New:** Suzukacillin A 04, 08: [Ala]^6^ → [Vxx]^6^	Krause et al., 2006b
																												**New:** Suzukacillin A 07: [Ala]^6^ → [Vxx]^6^	Krause et al., 2006b
																												**New:** Trichocellin TC-A-II, TC-A-IV: [Ala]^6^ → [Vxx]^6^	Wada et al., 1994
Pept-B-XXXIIIa	1936	1959	991	1163	774	38.98	Ac	Aib	Ala	Aib	Ala	Aib	Aib	Gln	Aib	Lxx	Aib	Gly	Aib	Aib	Pro	Vxx	Aib	Aib	Gln	Gln	Pheol	(Positional isomer of Pept-B-XX, XXXVa, XLIIb, XLVIa, and LVIII)	→ Pept-B-XX
Pept-B-XXXIIIb	1936	1959	991	1163	774	39.25	Ac	Aib	Ala	Aib	Ala	Aib	Ala	Gln	Aib	Lxx	Aib	Gly	Vxx	Aib	Pro	Vxx	Aib	Aib	Gln	Gln	Pheol	*Trichoderma citrinoviride* sequence 2 (Poitional isomer of Pept-XXXVb)	Maddau et al., 2009
Pept-B-XXXIIIc	1950	1973	998	1177	774	39.20	Ac	Aib	Ala	Aib	Ala	Aib	Aib	Gln	Aib	Lxx	Aib	Gly	Vxx	Aib	Pro	Vxx	Aib	Aib	Gln	Gln	Pheol	(Positional isomer of Pept-B-XXX, XLIIa, XLVIb, and LIII)	→ Pept-B-XXX
Pept-B-XXXIIId	1951	1974	998.5	1177	775	39.31	Ac	Aib	Ala	Aib	Ala	*Vxx*	Aib	Gln	Aib	Lxx	Aib	Gly	Aib	Aib	Pro	Vxx	Aib	Aib	*Glu*	Gln	Pheol	**New:** Paracelsin D: [Aib]^5^ → [*Vxx*]^5^ and [Aib]^18^ → [*Glu*]^18^	Pócsfalvi et al., 1997
																												**New:** Saturnisporin SA III: [Aib]^5^ → [*Vxx*]^5^ and [Aib]^18^ → [*Glu*]^18^	Rebuffat et al., 1993
																												**New:** Suzukacillin A 05: [Aib]^5^ → [*Vxx*]^5^ and [Aib]^18^ → [*Glu*]^18^	Krause et al., 2006b
Pept-B-XXXIVa	1937	1960	991.5	1149	789	39.59	Ac	Aib	Ala	Aib	Ala	Aib	Aib	Gln	Aib	Lxx	Ala	Gly	*Aib*	Aib	Pro	Vxx	Aib	Vxx	Glu	Gln	Pheol	**New:** Pept-1966-d: [Lxx]^12^ → [Aib]^12^	Tamandegani et al., 2016
Pept-B-XXXIVb	1951	1974	998.5	1163	789	39.13	Ac	Aib	Ala	Aib	Ala	Aib	*Aib*	Gln	Aib	Lxx	Aib	Gly	Aib	Aib	Pro	Vxx	Aib	Vxx	*Glu*	Gln	Pheol	**New:** (Positional isomer of Pept-B-XXXI and LII)	→ Pept-B-XXXI
Pept-B-XXXVa	1936	1959	991	1163	774	39.17	Ac	Aib	Ala	Aib	Ala	Aib	Aib	Gln	Aib	Lxx	Aib	Gly	Aib	Aib	Pro	Vxx	Aib	Aib	Gln	Gln	Pheol	(Positional isomer of Pept-B-XX, XXXIIIa, XLIIb, XLVIa, and LVIII)	→ Pept-B-XX
Pept-B-XXXVb	1936	1959	991	1163	774	39.78	Ac	Aib	Ala	Aib	Ala	Aib	Ala	Gln	Aib	Lxx	Aib	Gly	Vxx	Aib	Pro	Vxx	Aib	Aib	Gln	Gln	Pheol	(Positional isomer of Pept-B-XXXIIIb)	→ Pept-B-XXXIIIb
Pept-B-XXXVI	1964	1987	1005	1191	774	39.85	Ac	Aib	Ala	*Vxx*	Ala	*Vxx*	Aib	Gln	Aib	Lxx	Aib	Gly	Aib	Aib	Pro	Vxx	Aib	Aib	Gln	Gln	Pheol	**New:** Paracelsin D: [Aib]^3^ → [*Vxx*]^3^ and [Aib]^5^ → [*Vxx*]^5^	Pócsfalvi et al., 1997
																												**New:** Saturnisporin SA III: [Aib]^3^ → [*Vxx*]^3^ and [Aib]^5^ → [*Vxx*]^5^	Rebuffat et al., 1993
																												**New:** Suzukacillin A 05: [Aib]^3^ → [*Vxx*]^3^ and [Aib]^5^ → [*Vxx*]^5^	Krause et al., 2006b
Pept-B-XXXVII	1937	1960	991.5	1163	775	40.54	Ac	Aib	Ala	Aib	Ala	Aib	*Aib*	Gln	Aib	Lxx	Aib	Gly	Aib	Aib	Pro	Vxx	Aib	Aib	*Glu*	Gln	Pheol	**New:** (Positional isomer of Pept-B-XXV)	→ Pept-B-XXV
Pept-B-XXXVIII	1950	1973	998	1163	788	40.13	Ac	Aib	Ala	Aib	Ala	Aib	Aib	Gln	Aib	Lxx	Aib	Gly	Aib	Aib	Pro	Vxx	Aib	Vxx	Gln	Gln	Pheol	(Positional isomer of Pept-B-XXIV, XXVII, XXXIIa, and XLVa)	→ Pept-B-XXIV
Pept-B-XXXIX	1964	1987	1005	1177	788	39.57	Ac	Aib	Ala	Aib	Ala	*Vxx*	Aib	Gln	Aib	Lxx	Aib	Gly	Aib	Aib	Pro	Vxx	Aib	Vxx	Gln	Gln	Pheol	**New:** Saturnisporin SA IV: [Aib]^5^ → [*Vxx*]^5^	Rebuffat et al., 1993
																												**New:** Suzukacillin A 07: [Aib]^5^ → [*Vxx*]^5^	Krause et al., 2006b
Pept-B-XL	1950	1973	998	1163	788	40.55	Ac	Aib	Ala	Aib	Ala	Aib	*Ala*	Gln	Aib	Lxx	Aib	Gly	Vxx	Aib	Pro	Vxx	Aib	Vxx	Gln	Gln	Pheol	**New:** *Trichoderma citrinoviride* sequence 5: [Aib]^6^ → [*Ala*]^6^ (Positional isomer of Pept-B-XLVIIIa)	Maddau et al., 2009
																												**New:** *Trichoderma citrinoviride* sequence 6: [Aib]^6^ → [*Ala*]^6^	Maddau et al., 2009
																												**New:** *Trichoderma citrinoviride* sequence 8: [Aib]^6^ → [*Ala*]^6^	Maddau et al., 2009
Pept-B-XLIa	1964	1987	1005	1177	788	40.98	Ac	Aib	Ala	Aib	Ala	Aib	Aib	Gln	Aib	Lxx	Aib	Gly	Vxx	Aib	Pro	Vxx	Aib	Vxx	Gln	Gln	Pheol	*Trichoderma citrinoviride* sequence 5 (Positional isomer of Pept-B-XLVIIIb, LV, LVI, LXb, and LXI)	Maddau et al., 2009
																												*Trichoderma citrinoviride* sequence 6	Maddau et al., 2009
																												*Trichoderma citrinoviride* sequence 8	Maddau et al., 2009
Pept-B-XLIb	1964	1987	1005	1177	788	40.82	Ac	Aib	Ala	Aib	Ala	Aib	*Vxx*	Gln	Aib	Lxx	Aib	Gly	Aib	Aib	Pro	Vxx	Aib	Vxx	Gln	Gln	Pheol	**New:** (Positional isomer of Pept-B-XXXIIb)	→ Pept-B-XXXIIb
Pept-B-XLIIa	1950	1973	998	1177	774	41.54	Ac	Aib	Ala	Aib	Ala	Aib	Aib	Gln	Aib	Lxx	Aib	Gly	Vxx	Aib	Pro	Vxx	Aib	Aib	Gln	Gln	Pheol	(Positional isomer of Pept-B-XXX, XXXIIIc, XLVIb, and LIII)	→ Pept-B-XXX
Pept-B-XLIIb	1936	1959	991	1163	774	41.64	Ac	Aib	Ala	Aib	Ala	Aib	Aib	Gln	Aib	Lxx	Aib	Gly	Aib	Aib	Pro	Vxx	Aib	Aib	Gln	Gln	Pheol	Paracelsin D (Positional isomer of Pept-B-XX, XXXIIIa, XXXVa, XLVIa, and LVIII)	→ Pept-B-XX
Pept-B-XLIII	1964	1987	1005	1191	774	41.26	Ac	Aib	Ala	Aib	Ala	*Vxx*	*Vxx*	Gln	Aib	Lxx	Aib	Gly	Aib	Aib	Pro	Vxx	Aib	Aib	Gln	Gln	Pheol	**New:** Paracelsin B: [Aib]^5^ → [*Vxx*]^5^ and [Ala]^6^ → [*Vxx*]^6^	Pócsfalvi et al., 1997
																												**New:** Paracelsin D: [Aib]^5^ → [*Vxx*]^5^ and [Aib]^6^ → [*Vxx*]^6^	Pócsfalvi et al., 1997
																												**New:** Saturnisporin SA I: [Aib]^5^ → [*Vxx*]^5^ and [Ala]^6^ → [*Vxx*]^6^	Rebuffat et al., 1993
																												**New:** Saturnisporin SA III: [Aib]^5^ → [*Vxx*]^5^ and [Aib]^6^ → [*Vxx*]^6^	Rebuffat et al., 1993
																												**New:** Suzukacillin A 02, A 06: [Aib]^5^ → [*Vxx*]^5^ and [Ala]^6^ → [*Vxx*]^6^	Krause et al., 2006b
																												**New:** Suzukacillin A 05: [Aib]^5^ → [*Vxx*]^5^ and [Aib]^6^ → [*Vxx*]^6^	Krause et al., 2006b
																												**New:** Trichocellin TC-A-I, TC-A-III: [Aib]^5^ → [*Vxx*]^5^ and [Ala]^6^ → [*Vxx*]^6^	Wada et al., 1994
Pept-B-XLIV	1965	1988	1005.5	1191	775	41.65	Ac	Aib	Ala	Aib	Ala	*Vxx*	*Vxx*	Gln	Aib	Lxx	Aib	Gly	Aib	Aib	Pro	Vxx	Aib	Aib	Glu	Gln	Pheol	**New:** Trichocellin TC-B-I: [Aib]^5^ → [*Vxx*]^5^ and [Ala]^6^ → [*Vxx*]^6^ (Positionar isomer of Pept-B-L)	Wada et al., 1994
Pept-B-XLVa	1950	1973	998	1163	788	41.92	Ac	Aib	Ala	Aib	Ala	Aib	Aib	Gln	Aib	Lxx	Aib	Gly	Aib	Aib	Pro	Vxx	Aib	Vxx	Gln	Gln	Pheol	(Positional isomer of Pept-B-XXIV, XXVII, XXXIIa and XXXVIII)	→ Pept-B-XXIV
Pept-B-XLVb	1978	2001	1012	1191	788	42.47	Ac	Aib	Ala	*Vxx*	Ala	Aib	*Vxx*	Gln	Aib	Lxx	Aib	Gly	Aib	Aib	Pro	Vxx	Aib	Vxx	Gln	Gln	Pheol	**New:** Paracelsin H: [Aib]^3^ → [*Vxx*]^3^ and [Ala]^5^ → [*Vxx*]^5^ (Positional isomer of Pept-B-XLIX)	Pócsfalvi et al., 1997
																												**New:** Saturnisporin SA II: [Aib]^3^ → [*Vxx*]^3^ and [Ala]^6^ → [*Vxx*]^6^	Rebuffat et al., 1993
																												**New:** Saturnisporin SA IV: [Aib]^3^ → [*Vxx*]^3^ and [Aib]^6^ → [*Vxx*]^6^	Rebuffat et al., 1993
																												**New:** Suzukacillin A 04, 08: [Aib]^3^ → [*Vxx*]^3^ and [Ala]^6^ → [*Vxx*]^6^	Krause et al., 2006b
																												**New:** Suzukacillin A 07: [Aib]^3^ → [*Vxx*]^3^ and [Aib]^6^ → [*Vxx*]^6^	Krause et al., 2006b
																												**New:** Trichocellin TC-A-II, A-IV: [Aib]^3^ → [*Vxx*]^3^ and [Ala]^6^ → [*Vxx*]^6^	Wada et al., 1994
Pept-B-XLVIa	1936	1959	991	1163	774	42.42	Ac	Aib	Ala	Aib	Ala	Aib	Aib	Gln	Aib	Lxx	Aib	Gly	Aib	Aib	Pro	Vxx	Aib	Aib	Gln	Gln	Pheol	(Positional isomer of Pept-B-XX, XXXIIIa, XXXVa, XLIIb, and LVIII)	→ Pept-B-XX
Pept-B-XLVIb	1950	1973	998	1177	774	42.42	Ac	Aib	Ala	Aib	Ala	Aib	Aib	Gln	Aib	Lxx	Aib	Gly	Vxx	Aib	Pro	Vxx	Aib	Aib	Gln	Gln	Pheol	(Positional isomer of Pept-B-XXX, XXXIIIc, XLIIa, and LIII)	→ Pept-B-XXX
Pept-B-XLVII	1965	1988	1005.5	1177	789	42.13	Ac	Aib	Ala	Aib	Ala	*Vxx*	*Aib*	Gln	Aib	Lxx	Aib	Gly	Aib	Aib	Pro	Vxx	Aib	Vxx	*Glu*	Gln	Pheol	**New:** Trichocellin TC-B-II: [Aib]^5^ → [*Vxx*]^5^ and [Gln]^18^ → [*Glu*]^18^	Wada et al., 1994
																												**New:** Saturnisporin SA IV: [Aib]^5^ → [*Vxx*]^5^ and [Gln]^18^ → [*Glu*]^18^	Rebuffat et al., 1993
																												**New:** Suzukacillin A 07: [Aib]^5^ → [*Vxx*]^5^ and [Gln]^18^ → [*Glu*]^18^	Krause et al., 2006b
Pept-B-XLVIIIa	1950	1973	998	1163	788	43.00	Ac	Aib	Ala	Aib	Ala	Aib	*Ala*	Gln	Aib	Lxx	Aib	Gly	Vxx	Aib	Pro	Vxx	Aib	Vxx	Gln	Gln	Pheol	**New:** (Positional isomer of Pept-B-XL)	→ Pept-B-XL
Pept-B-XLVIIIb	1964	1987	1005	1177	788	42.85	Ac	Aib	Ala	Aib	Ala	Aib	Aib	Gln	Aib	Lxx	Aib	Gly	Vxx	Aib	Pro	Vxx	Aib	Vxx	Gln	Gln	Pheol	(Positional isomer of Pept-B-XLIa, LV, LVI, LXb, and LXI)	→ Pept-B-XLIa
Pept-B-XLIX	1978	2001	1012	1191	788	42.56	Ac	Aib	Ala	*Vxx*	Ala	Aib	*Vxx*	Gln	Aib	Lxx	Aib	Gly	Aib	Aib	Pro	Vxx	Aib	Vxx	Gln	Gln	Pheol	**New:** (Positional isomer of Pept-B-XLVb)	→ Pept-B-XLVb
Pept-B-L	1965	1988	1005.5	1191	775	42.67	Ac	Aib	Ala	Aib	Ala	*Vxx*	*Vxx*	Gln	Aib	Lxx	Aib	Gly	Aib	Aib	Pro	Vxx	Aib	Aib	Glu	Gln	Pheol	**New:** (Positionar isomer of Pept-B-XLIV)	→ Pept-B-XLIV
Pept-B-LI	1978	2001	1012	1205	774	43.24	Ac	Aib	Ala	*Vxx*	Ala	*Vxx*	*Vxx*	Gln	Aib	Lxx	Aib	Gly	Aib	Aib	Pro	Vxx	Aib	Aib	Gln	Gln	Pheol	**New:** Paracelsin B: [Aib]^3^ → [*Vxx*]^3^, [Aib]^5^ → [*Vxx*]^5^, and [Ala]^6^ → [*Vxx*]^6^	Pócsfalvi et al., 1997
																												**New:** Paracelsin D: [Aib]^3^ → [*Vxx*]^3^, [Aib]^5^ → [*Vxx*]^5^, and [Aib]^6^ → [*Vxx*]^6^	Pócsfalvi et al., 1997
																												**New:** Saturnisporin SA I: [Aib]^3^ → [*Vxx*]^3^, [Aib]^5^ → [*Vxx*]^5^, and [Ala]^6^ → [*Vxx*]^6^	Rebuffat et al., 1993
																												**New:** Saturnisporin SA III: [Aib]^3^ → [*Vxx*]^3^, [Aib]^5^ → [*Vxx*]^5^, and [Aib]^6^ → [*Vxx*]^6^	Rebuffat et al., 1993
																												**New:** Suzukacillin A 02, A 06: [Aib]^3^ → [*Vxx*]^3^, [Aib]^5^ → [*Vxx*]^5^, and [Ala]^6^ → [*Vxx*]^6^	Krause et al., 2006b
																												**New:** Suzukacillin A 05: [Aib]^3^ → [*Vxx*]^3^, [Aib]^5^ → [*Vxx*]^5^, and [Aib]^6^ → [*Vxx*]^6^	Krause et al., 2006b
																												**New:** Trichocellin TC-A-I, TC-A-III: [Aib]^3^ → [*Vxx*]^3^, [Aib]^5^ → [*Vxx*]^5^, and [Ala]^6^ → [*Vxx*]^6^	Wada et al., 1994
Pept-B-LII	1951	1974	998.5	1163	789	43.38	Ac	Aib	Ala	Aib	Ala	Aib	*Aib*	Gln	Aib	Lxx	Aib	Gly	Aib	Aib	Pro	Vxx	Aib	Vxx	*Glu*	Gln	Pheol	**New:** (Positional isomer of Pept-B-XXXI and XXXIVb)	→ Pept-B-XXXI
Pept-B-LIII	1950	1973	998	1177	774	43.99	Ac	Aib	Ala	Aib	Ala	Aib	Aib	Gln	Aib	Lxx	Aib	Gly	Vxx	Aib	Pro	Vxx	Aib	Aib	Gln	Gln	Pheol	(Positional isomer of Pept-B-XXX, XXXIIIc, XLIIa, and XLVIb)	→ Pept-B-XXX
Pept-B-LIV	1978	2001	1012	1191	788	44.00	Ac	Aib	Ala	Aib	Ala	*Vxx*	*Vxx*	Gln	Aib	Lxx	Aib	Gly	Aib	Aib	Pro	Vxx	Aib	Vxx	Gln	Gln	Pheol	**New:** Paracelsin H: [Aib]^5^ → [*Vxx*]^5^ and [Ala]^6^ → [*Vxx*]^6^	Pócsfalvi et al., 1997
																												**New:** Saturnisporin SA II: [Aib]^5^ → [*Vxx*]^5^ and [Aib]^6^ → [*Vxx*]^6^	Rebuffat et al., 1993
																												**New:** Saturnisporin SA IV: [Aib]^5^ → [*Vxx*]^5^ and [Ala]^6^ → [*Vxx*]^6^	Rebuffat et al., 1993
																												**New:** Suzukacillin A 04, 08: [Aib]^5^ → [*Vxx*]^5^ and [Ala]^6^ → [*Vxx*]^6^	Krause et al., 2006b
																												**New:** Suzukacillin A 07: [Aib]^5^ → [*Vxx*]^5^ and [Aib]^6^ → [*Vxx*]^6^	Krause et al., 2006b
																												**New:** Trichocellin TC-A-II, A-IV:[Aib]^5^ → [*Vxx*]^5^ and [Ala]^6^ → [*Vxx*]^6^	Wada et al., 1994
Pept-B-LV	1964	1987	1005	1177	788	44.44	Ac	Aib	Ala	Aib	Ala	Aib	Aib	Gln	Aib	Lxx	Aib	Gly	Vxx	Aib	Pro	Vxx	Aib	Vxx	Gln	Gln	Pheol	(Positional isomer of Pept-B-XLIa, XLVIIIb, LVI, LXb, and LXI)	→ Pept-B-XLIa
Pept-B-LVI	1950	1973	998	1163	788	45.07	Ac	Aib	Ala	Aib	Ala	Aib	Ala	Gln	Aib	Lxx	Aib	Gly	Vxx	Aib	Pro	Vxx	Aib	Vxx	Gln	Gln	Pheol	(Positional isomer of Pept-B-XLIa, XLVIIIb, LV, LXb, and LXI)	→ Pept-B-XLIa
Pept-B-LVII	1979	2002	1012.5	1191	789	45.36	Ac	Aib	Ala	Aib	Ala	*Vxx*	*Vxx*	Gln	Aib	Lxx	Aib	Gly	Aib	Aib	Pro	Vxx	Aib	Vxx	Glu	Gln	Pheol	**New:** Trichocellin TC-B-II: [Aib]^5^ → [*Vxx*]^5^ and [Ala]^6^ → [*Vxx*]^6^	Wada et al., 1994
Pept-B-LVIII	1936	1959	991	1163	774	45.81	Ac	Aib	Ala	Aib	Ala	Aib	Aib	Gln	Aib	Lxx	Aib	Gly	Aib	Aib	Pro	Vxx	Aib	Aib	Gln	Gln	Pheol	(Positional isomer of Pept-B-XX, XXXIIIa, XXVc, XLIIb, and XLVIa)	→ Pept-B-XX
Pept-B-LIX	1992	2015	1019	1205	788	45.74	Ac	Aib	Ala	*Vxx*	Ala	*Vxx*	*Vxx*	Gln	Aib	Lxx	Aib	Gly	Aib	Aib	Pro	Vxx	Aib	Vxx	Gln	Gln	Pheol	**New:** Paracelsin H: [Aib]^3^ → [*Vxx*]^3^, [Aib]^5^ → [*Vxx*]^5^, and [Ala]^6^ → [*Vxx*]^6^	Pócsfalvi et al., 1997
																												**New:** Saturnisporin SA II: [Aib]^3^ → [*Vxx*]^3^, [Aib]^5^ → [*Vxx*]^5^, and [Ala]^6^ → [*Vxx*]^6^	Rebuffat et al., 1993
																												**New:** Saturnisporin SA IV: [Aib]^3^ → [*Vxx*]^3^, [Aib]^5^ → [*Vxx*]^5^, and [Aib]^6^ → [*Vxx*]^6^	Rebuffat et al., 1993
																												**New:** Suzukacillin A 04, 08: [Aib]^3^ → [*Vxx*]^3^, [Aib]^5^ → [*Vxx*]^5^, and [Ala]^6^ → [*Vxx*]^6^	Krause et al., 2006b
																												**New:** Suzukacillin A 07: [Aib]^3^ → [*Vxx*]^3^, [Aib]^5^ → [*Vxx*]^5^, and [Aib]^6^ → [*Vxx*]^6^	Krause et al., 2006b
																												**New:** Trichocellin TC-A-II, A-IV: [Aib]^3^ → [*Vxx*]^3^, [Aib]^5^ → [*Vxx*]^5^, and [Ala]^6^ → [*Vxx*]^6^	Wada et al., 1994
Pept-B-LXa	1964	1987	1005	1177	788	46.50	Ac	Aib	Ala	Aib	Ala	Aib	Aib	Gln	Aib	Vxx	Aib	Gly	Lxx	Aib	Pro	Vxx	Aib	Vxx	Gln	Gln	Pheol	(Positional isomer of Pept-B-XLIXa)	→ Pept-B-XLIXa
Pept-B-LXb	1964	1987	1005	1177	788	46.29	Ac	Aib	Ala	Aib	Ala	Aib	Aib	Gln	Aib	Lxx	Aib	Gly	Vxx	Aib	Pro	Vxx	Aib	Vxx	Gln	Gln	Pheol	(Positional isomer of Pept-B-XLIa, XLVIIIb, LV, LVI, and LXI)	→ Pept-B-XLIa
Pept-B-LXI	1964	1987	1005	1177	788	48.35	Ac	Aib	Ala	Aib	Ala	Aib	Aib	Gln	Aib	Lxx	Aib	Gly	Vxx	Aib	Pro	Vxx	Aib	Vxx	Gln	Gln	Pheol	(Positional isomer of Pept-B-XLIa, XLVIIIb, LV, LVIII, and LXb)	→ Pept-B-XLIa

*Variable residues are UNDERLINED in the table header; minor sequence variants are UNDERLINED in the sequences. Amino acid exchanges in new compounds are set in italic*.

**Table 4:** Sequences of the newly identified brevicelsins (group C) from *Trichoderma* species of the Longibrachiatum Clade and their similarities to known peptaibols available in the “Comprehensive Peptaibiotics Database.”

**Peptide**	**M**	**[M+Na]^**+**^**	**[M+2Na]^**2+**^**	**b_**13**_**	**y_**7**_**	**rt-GK (min)**	**R**	**R1**	**R2**	**R3**	**R4**	**R5**	**R6**	**R7**	**R8**	**R9**	**R10**	**R11**	**R12**	**R13**	**R14**	**R15**	**R16**	**R17**	**R18**	**R19**	**R20**	**Compound identical or positionally isomeric with**	**References**
Brevicelsin-I	1851	1874	948.5	1078	774	28.72	Ac	Aib	Ala	Aib	Ala	Aib	-	Gln	Aib	Lxx	Aib	Gly	*Aib*	Aib	Pro	Vxx	Aib	Aib	Gln	Gln	Pheol	**New**: Hypophellin 18, 35, 39: [Lxx]^11^ → [*Aib*]^11^	Röhrich et al., 2013
																												**New**: Paracelsin B, D: [Ala]^6^, [Aib]^6^	Pócsfalvi et al., 1997
																												**New**: Saturnisporin SA I;, SA III: [Ala]^6^, [Aib]^6^	Rebuffat et al., 1993
																												**New**: Suzukacillin A 02, A 06, A 05: [Ala]^6^, [Aib]^6^	Krause et al., 2006b
																												**New**: Trichocellin TC-A-I, -III: [Ala]^6^	Wada et al., 1994
Brevicelsin-II	1865	1888	955.5	1092	774	29.98	Ac	Aib	Ala	*Vxx*	Ala	Aib	-	Gln	Aib	Lxx	Aib	Gly	*Aib*	Aib	Pro	Vxx	Aib	Aib	Gln	Gln	Pheol	**New**: HPV-9,−12,−20b: [Ala]^3^ → [*Vxx*]^3^, [Lxx]^11^ → [*Aib*]^11^	Röhrich et al., 2012
																												**New**: HPV-3: [Ser]^3^ → [*Vxx*]^3^, [Lxx]^11^ → [*Aib*]^11^	Röhrich et al., 2012
																												**New**: HPV-5: [Ala]^3^ → [*Vxx*]^3^, [Vxx]^11^ → [*Aib*]^11^	Röhrich et al., 2012
																												**New**: Hypophellin 17, 34: [Ala]^3^ → [*Vxx*]^3^, [Lxx]^11^ → [*Aib*]^11^	Röhrich et al., 2013
																												**New**: Hypophellin 20, 40: [Aib]^3^ → [*Vxx*]^3^, [Lxx]^11^ → [*Aib*]^11^	Röhrich et al., 2013
																												**New**: Hypophellin 18, 35, 39: [Aib]^3^ → [*Vxx*]^3^, [Lxx]^11^ → [*Aib*]^11^	Röhrich et al., 2013
																												**New**: Hypophellin 31: [Ser]^3^ → [*Vxx*]^3^, [Lxx]^11^ → [*Aib*]^11^	Röhrich et al., 2013
																												**New**: Paracelsin B, D: [Aib]^3^ → [Vxx]^3^ [Ala]^6^, [Aib]^6^,	Pócsfalvi et al., 1997
																												**New**: Saturnisporin SA I; SA III: [Aib]^3^ → [Vxx]^3^, [Ala]^6^, [Aib]^6^	Rebuffat et al., 1993
																												**New**: Suzukacillin A 02, A 06, A 05: [Aib]^3^ → [Vxx]^3^, [Ala]^6^, [Aib]^6^	Krause et al., 2006b
																												**New**: Trichocellin TC-A-I, -III: [Aib]^3^ → [Vxx]^3^, [Ala]^6^	Wada et al., 1994
Brevicelsin-III	1852	1875	949	1078	775	30.56	Ac	Aib	Ala	Aib	Ala	Aib	-	Gln	Aib	Lxx	Aib	Gly	*Aib*	Aib	Pro	Vxx	Aib	Aib	Glu	Gln	Pheol	**New**: Hypophellin 38: [Lxx]^11^ → [*Aib*]^11^	Röhrich et al., 2013
																												**New**: Trichocellin TC-A-I, -III: [Ala]^6^, [Aib]^6^	Wada et al., 1994
Brevicelsin-IV	1865	1888	955.5	1078	788	31.82	Ac	Aib	Ala	Aib	Ala	Aib	-	Gln	Aib	Lxx	Aib	Gly	*Aib*	Aib	Pro	Vxx	Aib	Vxx	Gln	Gln	Pheol	**New**: Hypophellin 20, 40: [Lxx]^11^ → [*Aib*]^11^	Röhrich et al., 2013
																												**New**: Paracelsin H: [Ala]^6^	Pócsfalvi et al., 1997
																												**New**: Saturnisporin SA II, IV: [Ala]^6^, [Aib]^6^	Rebuffat et al., 1993
																												**New**: Suzukacillin A 04, A 08, 07: [Ala]^6^, [Aib]^6^	Krause et al., 2006b
																												**New**: Trichocellin TC-A-II, -IV: [Ala]^6^	Wada et al., 1994
Brevicelsin-V	1879	1902	962.5	1092	788	32.82	Ac	Aib	Ala	Aib	Ala	*Vxx*	-	Gln	Aib	Lxx	Aib	Gly	*Aib*	Aib	Pro	Vxx	Aib	Vxx	Gln	Gln	Pheol	**New**: Hypophellin 22, 45: [Lxx]^11^ → [*Aib*]^11^ (Positional isomer of Brevicelsin VIII)	Röhrich et al., 2013
																												**New**: Paracelsin H: [Aib]^5^ → [*Vxx*]^5^, [Ala]^6^	Pócsfalvi et al., 1997
																												**New**: Saturnisporin SA II, IV: [Aib]^5^ → [*Vxx*]^5^, [Ala]^6^, [Aib]^6^	Rebuffat et al., 1993
																												**New**: Suzukacillin A 04, A 08, 07: [Aib]^5^ → [*Vxx*]^5^, [Ala]^6^, [Aib]^6^	Krause et al., 2006b
																												**New**: Trichocellin TC-A-II, -IV: [Aib]^5^ → [*Vxx*]^5^, [Ala]^6^	Wada et al., 1994
Brevicelsin-VI	1865	1888	955.5	1092	774	33.20	Ac	Aib	Ala	Aib	Ala	*Vxx*	-	Gln	Aib	*Lxx*	Aib	Gly	*Aib*	Aib	Pro	Vxx	Aib	Aib	Gln	Gln	Pheol	**New**: Hypophellin 4: [Lxx]^11^ → [*Aib*]^11^	Röhrich et al., 2013
																												**New**: Paracelsin B, D: [Aib]^5^ → [*Vxx*]^5^, [Ala]^6^, [Aib]^6^,	Pócsfalvi et al., 1997
																												**New**: Saturnisporin SA I;, SA III: [Aib]^5^ → [*Vxx*]^5^, [Ala]^6^, [Aib]^6^	Rebuffat et al., 1993
																												**New**: Suzukacillin A 02, A 06, A 05: [Aib]^5^ → [*Vxx*]^5^, [Ala]^6^, [Aib]^6^	Krause et al., 2006b
																												**New**: Trichocellin TC-A-I, -III: [Aib]^5^ → [*Vxx*]^5^, [Ala]^6^	Wada et al., 1994
Brevicelsin-VII	1866	1889	956	1078	789	33.65	Ac	Aib	Ala	Aib	Ala	Aib	-	Gln	Aib	Lxx	*Aib*	Gly	*Aib*	Aib	Pro	Vxx	Aib	Vxx	Glu	Gln	Pheol	**New**: Hypophellin 21, 43: [Lxx]^11^ → [*Aib*]^11^	Röhrich et al., 2013
																												**New**: Hypocitrinin-7: [Lxx]^11^ → [*Aib*]^11^	Röhrich et al., 2014
Brevicelsin-VIII	1879	1902	962.5	1092	788	36.19	Ac	Aib	Ala	Aib	Ala	*Vxx*	-	Gln	Aib	Lxx	*Aib*	Gly	*Aib*	Aib	Pro	Vxx	Aib	Vxx	Gln	Gln	Pheol	**New**: (Positional isomer of Brevicelsin V)	→ Brevicelsin V

*Variable residues are UNDERLINED in the table header; minor sequence variants are UNDERLINED in the sequences. Amino acid exchanges in new compounds are set in italic*.

Of the 49 sequences from group A consisting exclusively of 20-residue peptaibols, 27 have been previously described in the literature, and 22 were new, differing by 1–3 amino acids from known sequences (Table 2). Group B also comprises 20-residue sequences (Table 3). The main difference between group B and group A peptaibols is located at the R12 position, where Aib instead of Lxx is present in most of the group B sequences. Another major difference from group A is that the R5 position is not conserved due to a high percentage of Vxx instead of Aib. Of the 86 group B sequences, 37 were identified as new. An entirely new compound, Pept-B-LIX, with a mass of 1992 Da was detected in the crude extracts of three strains (*T*. *konilangbra* SzMC 22607, *T*. *flagellatum* SzMC 22608 and *T*. *sinensis* SzMC 22609). All sequences of group C produced by three strains (*T*. *flagellatum* SzMC 22608, *T*. *sinensis* SzMC 22609 and *T*. *parareesei* SzMC 22615) proved to belong to a new group of peptaibols, which was named brevicelsins, as they are similar to, but one amino acid shorter than paracelsins (Brückner and Graf, 1983; Pócsfalvi et al., 1997) (Table 4).

### Qualitative and Semi-quantitative Peptaibol Profiles of the Strains

After investigation of all strains producing peptaibols from group A, “a” and “b” versions of their peptaibol compounds were apparent. Pept-A-XI has a “c” version of the compound, and a few others are represented by only a single sequence (Supplementary Table 7). Compounds such as Pept-A-IV-a and -b were produced constantly in high quantities by all strains. Both Pept-A-IX-a and -b were produced in high quantities by all strains except *T*. *aethiopicum* SzMC 22602, *T*. *pinnatum* SzMC 22603 and *T*. *longibrachiatum* SzMC 1775. Similarly, Pept-A-XVI-a and -b were produced by all strains. In this group, seven mainly produced peptaibol varieties appeared on the spectra, Pept-A-IV-a and -b, Pept-A-VI-a and -b, Pept-A-IX-a and -b, Pept-A-XV-a and -b, Pept-A-XVI-a and -b, Pept-A-XIXa as well as Pept-A-XXI-a and -b.

The analysis of the four *T*. *longibrachiatum* strains (SzMC 1773, 1775, 1776, and 12546) revealed similar, but still different, profiles (Supplementary Table 8). Environmental isolates of *T*. *longibrachiatum* produced more similar profiles, whereas the peptaibol profile of the clinical isolate was different from those of the three environmental strains. Pept-B-XX and Pept-B-XXVII were produced by all of the strains examined, whereas the other compounds were produced only by certain strains. Five peptaibol compounds (Pept-B-VII, Pept-B-XVII, Pept-B-XX, Pept-B-XXVII, and Pept-XLV-a and b) were produced at high levels. Certain strains could also produce other compounds, such as Pept-B-XXVIII, Pept-B-XXIX-a and b, Pept-B-XXXIIIa, Pept-A-IVb, Pept-XLIb, Pept-XLIII, Pept-B-XLVa, Pept-B-LI, Pept-B-LIV, and Pept-B-LVIb, at high levels. The most diverse peptaibol profile was observed in *T*. *reesei* QM6a (SzMC 22614), which produced 41 different peptaibol compounds, whereas the least diverse profiles were that of *T*. *effusum* SzMC 22611 and *T*. *konilangbra* SzMC 22607, which produced 11 and 12 sequences, respectively. Some species producing mostly group B peptaibols, *T. reesei* QM6A (SzMC 22614), *T. saturnisporum* SzMC 22606 and *T. konilangbra* SzMC 22607 could also produce peptaibols from group A. Interestingly, group A sequences could not be detected from the two mutant strains of *T. reesei* SzMC 22614 (*T. reesei* SzMC 22616 and SzMC 22617). Brevicelsins from group C were only produced by three species, *T. sinensis, T. flagellatum* and, to a lesser extent, *T. parareesei*. Brevicelsin I and Brevicelsin IV were produced by the examined strains (*T*. *flagellatum* SzMC 22608, *T*. *sinensis* SzMC 22609 and *T*. *parareesei* SzMC 22615) of all three species, but *T*. *parareesei* produced only these two compounds of group C in addition to the group B sequences.

**Figure 1:**
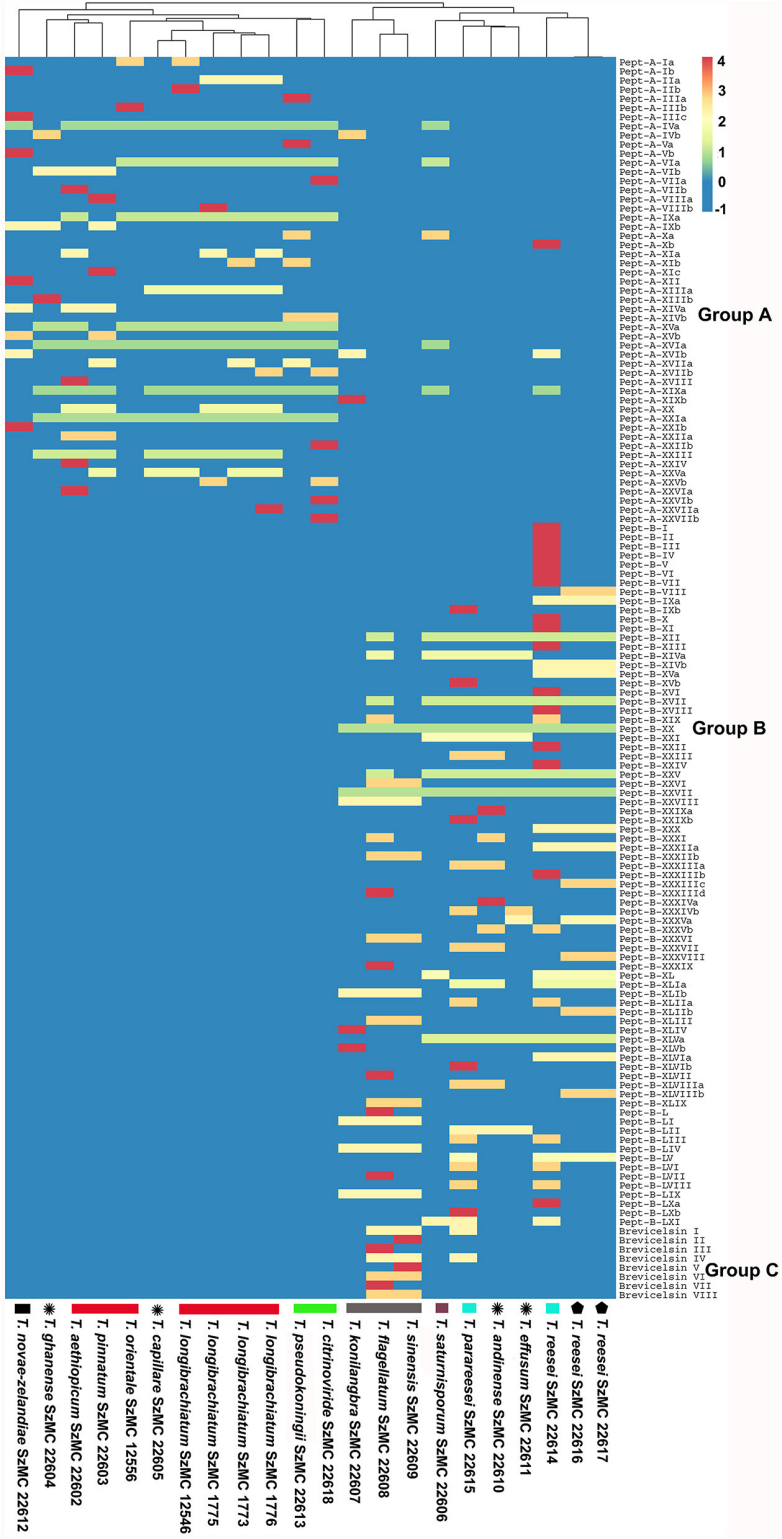
Heatmap showing the correlation between the production of peptaibols and the phylogenetic relationship between the strains. Monophyletic species are indicated by the bottom bar of the same color, species attributed to single phylogenetic lineages are marked with a star, while mutant strains are indicated with a filled pentagon. The color scale denotes production level increasing from zero (deep blue) to high (deep red).

We carried out a cluster analysis of the peptaibol diversity profiles in different *Trichoderma* species of the Longibrachiatum Clade based on the production levels of different peptaibols by various fungal producers (Supplementary Tables 7, 8). According to their peptaibol profiles, members of the Longibrachiatum Clade were divided into two main clusters (Figure 1). The first cluster involves species producing exclusively group A peptaibols. Among them, *T. novae-zelandiae* is characterized with a relatively poor, but sharply distinct, profile of abundantly produced peptaibol compounds from group A, like Pept-A-XXIb, XVIb, XII, Vb, Ib, and IIIc. Further species in this cluster include members of the phylogenetic subclades Longibrachiatum/Orientale and Citrinoviride/Pseudokoningii, along with the lone lineages *T. ghanense* and *T. capillare* (Table 1). This cluster is consisting of three subclusters, the first one containing the closely related species *T. aethiopicum* and *T. pinnatum* and the second one involving *T. longibrachiatum* and *T. orientale*—all belonging to the phylogenetic subclade Longibrachiatum/Orientale—while the third subcluster is corresponding with the subclade Citrinoviride/Pseudokoningii (Table 1; Figure 1). The second main cluster is comprised of species producing mainly group B peptaibols and includes 2 subclusters, with the first containing the phylogenetic subclades Parareesei/Reesei, Saturnisporum and the lone lineages *T. andinense* and *T. effusum*, while the second harboring the three examined species from subclade Konilangbra/Sinensis (Table 1; Figure 1). All three examined members of this subclade produced the entirely new compound Pept-B-LIX (1992 Da).

### Annotation of NRPS Domains From the Genomes of *T. longibrachiatum, T. citrinoviride, T. reesei*, and *T. parareesei*

**Figure 2:**
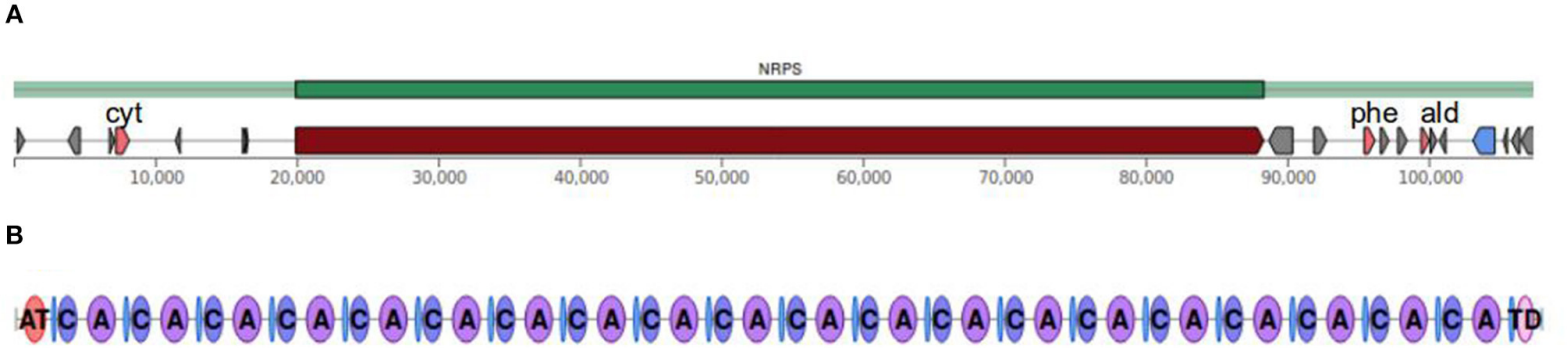
Schematic structure of the 20-residue non-ribosomal peptide synthase (NRPS) from *T*. *longibrachiatum*. **(A)** Up- and downstream regions of the gene encoding the NRPS enzyme; pink pentagons indicate genes of additional putative biosynthetic enzymes (cyt: cytochrome P450, phe: phenylalanine-specific permease, ald: aldo/keto reductase). **(B)** The 20 modules of the NRPS. AT, acyl transferase; C, condensation module; A, adenylation module; TD, thioesterase domain.

The NRPS gene sequences from *T*. *longibrachiatum* (https://genome.jgi.doe.gov/Trilo1/Trilo1.home.html, Xie et al., 2014), *T*. *citrinoviride* (https://genome.jgi.doe.gov/Trici4/Trici4.home.html), *T*. *reesei* (https://genome.jgi.doe.gov/Trire2/Trire2.home.html, Martinez et al., 2008) and *T*. *parareesei* (NCBI Bioproject Id: PRJNA287603, Yang et al., 2015) predicted by the SMIPS software were analyzed using the fungiSMASH software pipeline (Blin et al., 2017), which was designed to identify gene clusters of secondary metabolite biosynthesis from nucleotide sequences and to predict the products of the clusters identified. The *T*. *longibrachiatum, T*. *citrinoviride, T*. *reesei*, and *T*. *parareesei* genome sequences contain genes encoding 20-module NRPSs of 69.505, 68.508, 69.516, and 69.516 bp, as well as 14-module NRPSs of 43.422, 44.196, 49.386, and 52.395 bp with adenylation, condensation, thiolation, single acyl transferase and thioesterase domains. Figure 2 shows the schematic structure of the 20-mer NRPS gene cluster and the encoded modular enzyme from *T*. *longibrachiatum*. The 5′ ends of 20-module synthetase sequences contain a ketide synthase, whereas a Phe-specific permease-like and an aldo/keto reductase-like gene can be found downstream from the NRPS gene cluster. These two genes were also identified in the region downstream of the 18-module peptaibol synthetase gene clusters of the mushroom green mold agents *T*. *aggressivum* and *T*. *pleuroti* (Marik et al., 2017a). The identification of the presence of Pro in the peptaibol sequences and the close proximity of a Pro-specific permease gene to the NRPS gene cluster in these six *Trichoderma* species suggests that the permease may have a role in the secretion of these secondary metabolites.

Table 5 shows the incorporated amino acids predicted by the NRPS/PKS substrate predictor and NRPSPredictor3, based on the annotated adenylation domains and the eight amino acid residue signature sequences. The four 20-module NRPSs from the Longibrachiatum Clade were identical in positions R15 and R16 according to the signature sequences and the incorporated amino acids, respectively. Two positions (R6 and R9) were different only in *T*. *longibrachiatum*, whereas position R17 showed identity between *T*. *longibrachiatum*/*T*. *citrinoviride*, and *T*. *reesei*/*T*. *parareesei*. The most variable position was predicted to be R12, in which all signature sequences differed, and the incorporated amino acid was different in the case of *T*. *citrinoviride*.

**Table 5:** Comparison of signature sequences of NRPS modules and predicted amino acid incorporations with the detected amino acid composition of the 20-residue peptaibols in the case of four *Trichoderma* species from the Longibrachiatum Clade.

	***Trichoderma reesei***			***Trichoderma citrinoviride***			***Trichoderma longibrachiatum***			***Trichoderma parareesei***		
**Amino acid position in peptaibols**	**Sequence of amino acid binding pocket in NRPS module**	**Possible amino acids predicted NRPS/PKS substrate predictor/ NRPSPredictor3 SVM**	**Amino acids detected in peptaibol sequences**	**Sequence of amino acid binding pocket in NRPS module**	**Possible amino acids predicted NRPS/PKS substrate predictor /NRPSPredictor3 SVM**	**Amino acids detected in peptaibol sequences**	**Sequence of amino acid binding pocket in NRPS module**	**Possible amino acids predicted NRPS /PKS substrate predictor /NRPSPredictor3 SVM**	**Amino acids detected in peptaibol sequences**	**Sequence of amino acid binding pocket in NRPS module**	**Possible amino acids predicted NRPS /PKS substrate predictor/ NRPSPredictor3 SVM**	**Amino acids detected in peptaibol sequences**
1	D L G Y L A G V	Aib, Iva/**Iva**	Aib	D L G Y L A G V	Aib, Iva/**Iva**	Aib	D L G Y L A G V	Aib, Iva/**Iva**	Aib	D L G Y L A G V	Aib, Iva**/Iva**	Aib
2	D I L F N G L I	Ala/–	Ala	D I L F N G L I	Ala/–	Ala	D I L F N G L I	Ala/–	Ala	D I L F N G L I	Ala/–	Ala
3	D L G F L A G V	Aib, Iva/**Iva**	Aib, Ala	D L G F L A G V	Aib, Iva/**Iva**	Aib	D L G F L A G V	Aib, Iva/**Iva**	Aib, Iva	D L G F L A G V	Aib, Iva/**Iva**	Aib
4	D V G F V A G V	Aib, Iva/**ala**	Ala	D V G F V A G V	Aib, Iva/**ala**	Ala	D V G F V A G V	Aib, Iva/**ala**	Ala	D V G F V A G V	Aib, Iva/**ala**	Ala
5	D L G F L A G V	Aib, Iva/**Iva**	Aib	D L G F L A G V	Aib, Iva/**Iva**	Aib	D L G F L A G V	Aib, Iva/**Iva**	Aib	D L G F L A G V	Aib, Iva/**Iva**	Aib
6	D V G C I E G V	Aib, Iva/**Iva**	Ala	D V G C I E G V	Aib, Iva/**Iva**	Ala	D V G C I A G V	Aib, Iva/–	Ala	D V G C I E G V	Aib, Iva/**Iva**	Ala
7	D G G M V G G N	Gln/**Gln**	Gln	D G G M V G G N	Gln/**Gln**	Gln	D G G M V G G N	Gln/**Gln**	Gln	D G G M V G G N	Gln**/Gln**	Gln
8	D L G Y L A G V	Aib, Iva/**Iva**	Aib	D L G Y L A G V	Aib, Iva/**Iva**	Aib	D L G Y L A G V	Aib, Iva/**Iva**	Aib	D L G Y L A G V	Aib, Iva/**Iva**	Aib
9	D A F L I G G V	Aib, Iva/**Leu**	Iva, Ile	D A F L I G G V	Aib, Iva/**Leu**	Iva, Ile	D A F L L G A V	Ala/**Als**	Iva, Ile	D A F L I G A V	Aib, Iva/**Leu**	Iva, Ile
10	D L G Y L A G V	Aib, Iva/**Iva**	Aib	D L G Y L A G V	Aib, Iva/**Iva**	Aib	D L G Y L A G V	Aib, Iva/**Iva**	Aib	D L G Y L A G V	Aib, Iva/**Iva**	Aib
11	D V G Y L I A V	Aib, Iva/–	Gly	D V G Y L I A V	Aib, Iva/–	Gly	D V G Y L I A V	Aib, Iva/–	Gly	D V G Y L I A V	Aib, Iva/–	Gly
12	D L G Y L A G -	Aib, Iva/–	Ile, Iva, Aib	D L G Y L A G V	Aib, Iva/**ala**	Ile	D F G F L G A V	Aib, Iva/–	Ile, Iva	D L A Y L A G -	Aib, Iva/–	Ala, Iva, Aib
13	D L G F L A G V	Aib, Iva/**Iva**	Aib	D L G F L A G V	Aib, Iva/**Iva**	Aib	D L G F L A G V	Aib, Iva/**Iva**	Aib	D L G Y L A G V	Aib, Iva/**Iva**	Aib
14	D V L F C G L I	Pro/–	Pro	D V L F C G L I	Pro**/–**	Pro	D V L F C G L I	Pro/–	Pro	D V L F C G L I	Pro**/–**	Pro
15	D A G M I I G V	Aib, Iva/**Iva**	Iva	D A G M I I G V	Aib, Iva/**Iva**	Iva	D A G M I I G V	Aib, Iva/**Iva**	Iva	D A G M I I G V	Aib, Iva/**Iva**	Iva
16	D L G F L A G V	Aib, Iva/**Iva**	Aib	D L G F L A G V	Aib, Iva/**Iva**	Aib	D L G F L A G V	Aib, Iva/**Iva**	Aib	D L G F L A G V	Aib, Iva/**Iva**	Aib
17	D M G W F A G -	Aib, Iva**/Iva**	Aib, Iva	D M G W F A G V	Aib, Iva**/Iva**	Aib, Iva	D M G W F A G V	Aib, Iva**/Iva**	Aib, Iva	D M G W F A G -	Aib, Iva**/Iva**	Aib, Iva
18	D G G M V G G N	Gln/**Gln**	Glu, Gln	D G G M V G G N	Gln/**Gln**	Glu, Gln	D G G M V G G N	Gln/**Gln**	Glu, Gln	D G G M V G G N	Gln/**Gln**	Glu, Gln
19	D G G M V G G N	Gln/**Gln**	Gln	D G G M V G G N	Gln/**Gln**	Gln	D G G M V G G N	Gln/**Gln**	Gln	D G G M I G G N	Gln**/Gln**	Gln
20	D A A F I M G V	–/–	Pheol	D A A F I M G V	–/–	Pheol	D A A F I M G V	–/–	Pheol	D A A F I M G V	–/–	Pheol

Comparison of the amino acids predicted by the NRPS/PKS substrate predictor and the ones detected showed agreement at 11 positions in all four species. In positions R6, R11 and R18, the prediction did not match with the detected Ala, Gly and Glu, respectively. Position R11 of the four species showed identity with position R10 of *T*. *aggressivum* (Marik et al., 2017a) in its signature sequence (DVGYLIAV), but the amino acid prediction in these positions was incorrect in all cases. At the last position, the predictor software identified the signature sequence of adenylation domains, but the amino acid prediction failed. These unsuccessful predictions suggest that these signature sequences are missing from the database. Based on the signature sequences, the highest variability is in position R12, where the amino acids detected are also variable.

### Structural Characterization of 20- and 19-Residue Peptaibols

Two previously described sequences, Paracelsin B (AcAib-Ala- Aib-Ala-Aib-Ala-Gln-Aib-Leu-Aib-Gly-Aib-Aib-Pro-Val-Aib-Aib-Gln-Gln-Pheol) and Paracelsin H (AcAib-Ala-Aib-Ala-Aib-Ala-Gln-Aib-Leu-Aib-Gly-Aib-Aib-Pro-Val-Aib-Val-Gln-Gln-Pheol), together with their 19-residue counterparts Brevicelsin I (AcAib-Ala-Aib-Ala-Aib-Gln-Aib-Leu-Aib-Gly-Aib-Aib-Pro- Val-Aib-Aib-Gln-Gln-Pheol) and Brevicelsin IV (AcAib-Ala- Aib-Ala-Aib-Gln-Aib-Leu-Aib-Gly-Aib-Aib-Pro-Val-Aib-Val-Gln-Gln-Pheol) were selected for structural characterization. Based on their sequences, Paracelsin B and H appear to correspond with Pept-B-XII and Pept-B-XVIII, respectively, both of which were produced by six examined species (*T*. *reesei, T*. *saturnisporum, T*. *andinense, T*. *effusum, T*. *parareesei*, and *T*. *flagellatum*). Our aim was to observe structural differences resulting from the loss of Ala at the R6 position.

**Figure 3:**
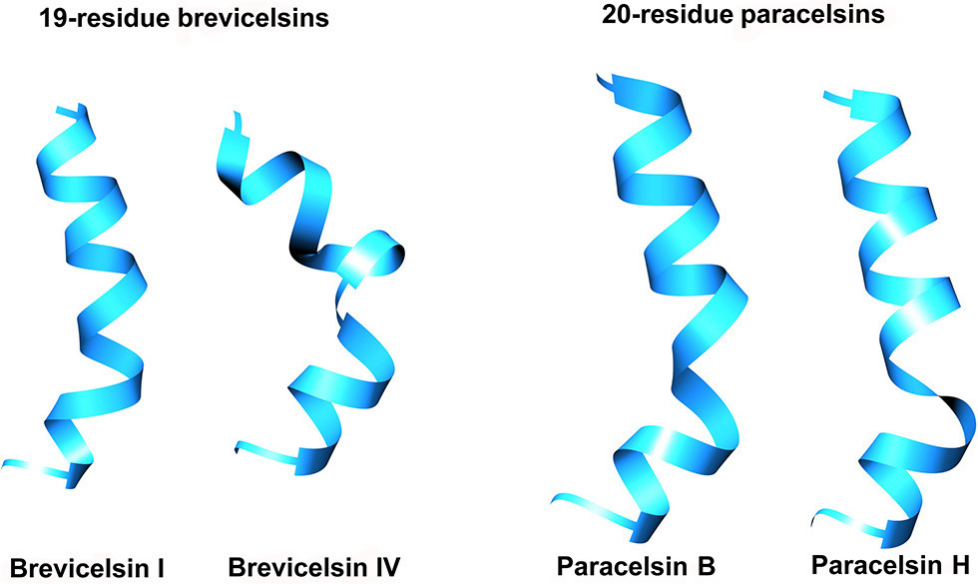
Representative structures of the most populated clusters for each peptaibol simulation. The simulations were carried out for 1,000 ns in each case.

**Figure 4:**
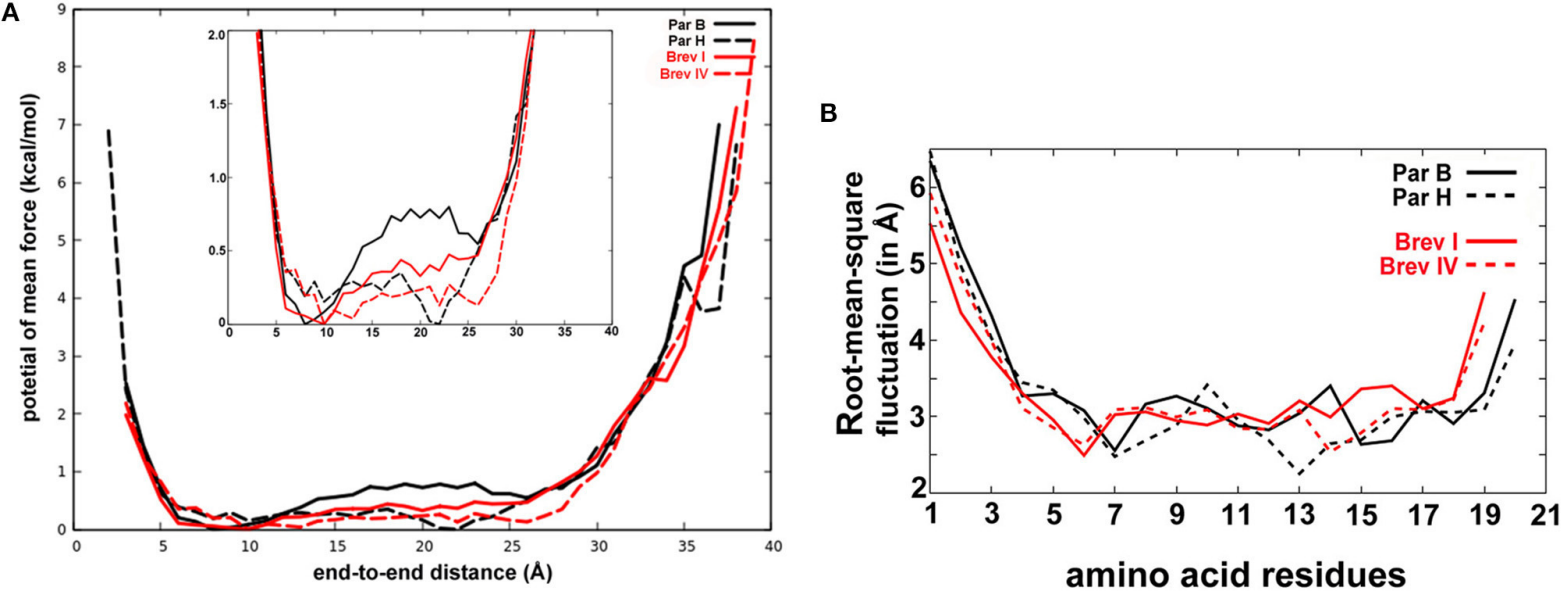
**(A)** The reweighted potential of mean force (PMF in kcal mol^−1^) values calculated for end-to-end distance (in Å). The 20-residue paracelsins have been plotted in black, whereas 19-residue brevicelsins are shown in red. The zoomed version of the plot is provided in the inset. The end-to-end values where PMF is zero or close to zero are the energetically stable values and define the most favorable linear conformation. **(B)** The root-mean-square deviation (in Å) calculated for each residue for all sequences gives an idea of the average fluctuation undergone by the system. Par, Paracelsin; Brev, Brevicelsin.

All peptides show a strong tendency to form right-handed helical structures with a slight bend at the Aib-Pro position (Figure 3). Cluster analysis of the simulation trajectories of all four peptaibols revealed different energetically stable conformations that occur during folding, and the representative structures of the most populated cluster are provided for each peptaibol. All peptides fold into an energetically favored, highly bent helical conformation along with a linear helical conformation. Based on the reweighted potential of mean force (PMF) values calculated for end-to-end distance (distance in Å from the N-terminus to the C-terminus), it can be speculated that a highly curved conformation for all peptaibols, except for Paracelsin H, lies in the energy minimum and requires an energy “jump” of <1 kcal mol^−1^ to attain the linear backbone conformation (Figure 4A). Overall, the end-to-end distance values as low as 5 to 27 Å, that lie close to the energy minima, show that all conformations starting from a hairpin-like helix structure to a straight backbone with just a slight bend are easily accessible. The PMF values increase rapidly beyond these two points for all four peptaibols, as shown in the inset image focusing only on PMF values up to 2 kcal mol^−1^. However, the sequences Paracelsin B and Brevicelsin I, with an Aib residue in position R17, have higher PMF values for higher end-to-end distance values; the energy cost for attaining linearity of the helical backbone is slightly higher than in Paracelsin H and Brevicelsin IV, where a Val residue replaces Aib in the R17 position. The energy minimum for Paracelsin H lies at an end-to-end distance of 22 Å, whereas Brevicelsin IV exhibits a slight fall at this point, even though its energy minimum also lies at 10 Å. The presence of Aib residue in position R17 (in Paracelsin B and Brevicelsin I) results in a highly dynamic folding process, which means that many conformations were visited during the trajectory, whereas Val in the same position (in Paracelsin H and Brevicelsin IV) led to fewer energetically stable conformers. The root-mean-square-atomic fluctuation (RMSF) graph (Figure 4B) shows higher fluctuation of N-terminus region for all peptides. No other significant differences were observed between the RMSF values of the 19-residue peptaibols, Brevicelsins I and IV, in comparison to 20-residue peptaibols, Paracelsins B and H, except that the sequences containing more Aib residues show a slight elevation in atomic fluctuation at the corresponding sequence position. For example, at R16 for Brevicelsin I and R17 for Paracelsin B, also, the R6 Aib in Paracelsins B and H shows higher average atomic fluctuation than the R6 Gln of Brevicelsins I and IV. This observation establishes the fluctuating and dynamic nature of the Aib residue in peptaibol sequences which can be explained by its tendency to oscillate between right- and left-handed helical forms. The Gln residues at R7 and R6 positions of paracelsins and brevicelsins, respectively, show a sharp dip in atomic fluctuation indicating higher stability in comparison to the C-terminal Gln residues and highlights importance of glutamines in ion-channel stabilization (Whitmore and Wallace, 2004).

### Antifungal Effects of *T. reesei* Peptaibols on Filamentous Fungi

The purified peptaibol extracts of *T*. *reesei* QM9414 were tested on human and plant pathogenic filamentous fungi, furthermore, the producer strain itself, as well as its Δ*lae*1 mutant (Table 6). Treatment with 0.4 and 0.2 mg ml^−1^ purified peptaibol solution resulted in growth inhibition of all strains, whereas a weaker, but still notable, inhibition was detected after treatment with the purified extract at a concentration of 0.1 mg ml^−1^. The peptaibol extract from *T*. *reesei* QM9414 exhibited an inhibition profile highly similar to that of alamethicin.

**Table 6:** Antifungal activity of the purified peptaibol extract from *T. reesei* QM9414 to filamentous fungi.

**Tested filamentous fungal strain**	**MIC of purified peptaibol extract (mg ml^**−1**^)**	MIC of alamethicin^*^ standard (mg ml^**−1**^)	**MIC of nystatin standard (mg ml^**−1**^)**
*Alternaria alternata* SzMC 16085	0.1	0.05	0.003125
*Aspergillus fumigatus* SzMC 23245	0.1	0.1	0.0125
*Fusarium falciforme* SzMC 11407	0.05	0.05	0.025
*Fusarium keratoplasticum* SzMC 11414	0.1	0.1	0.05
*Fusarium solani* SC SzMC 11467	0.1	0.1	0.05
*Phoma cucurbitacearum* SZMC 16088	0.05	0.05	0.1
*Trichoderma reesei* QM9414	0.1	0.05	0.00625
*Trichoderma reesei* QM9414 G2*Δlae*1	0.05	0.05	0.00625

*SC, species complex*.

* *Harzianum A contamination could not be detected in the alamethicin standard based on the exact mass of its deprotonated molecular ion ([M-H]^−^, m/z = 399.1808)*.

### Bioactivities of *T*. *reesei* Peptaibols on *Arabidopsis thaliana* Plants

**Figure 5:**
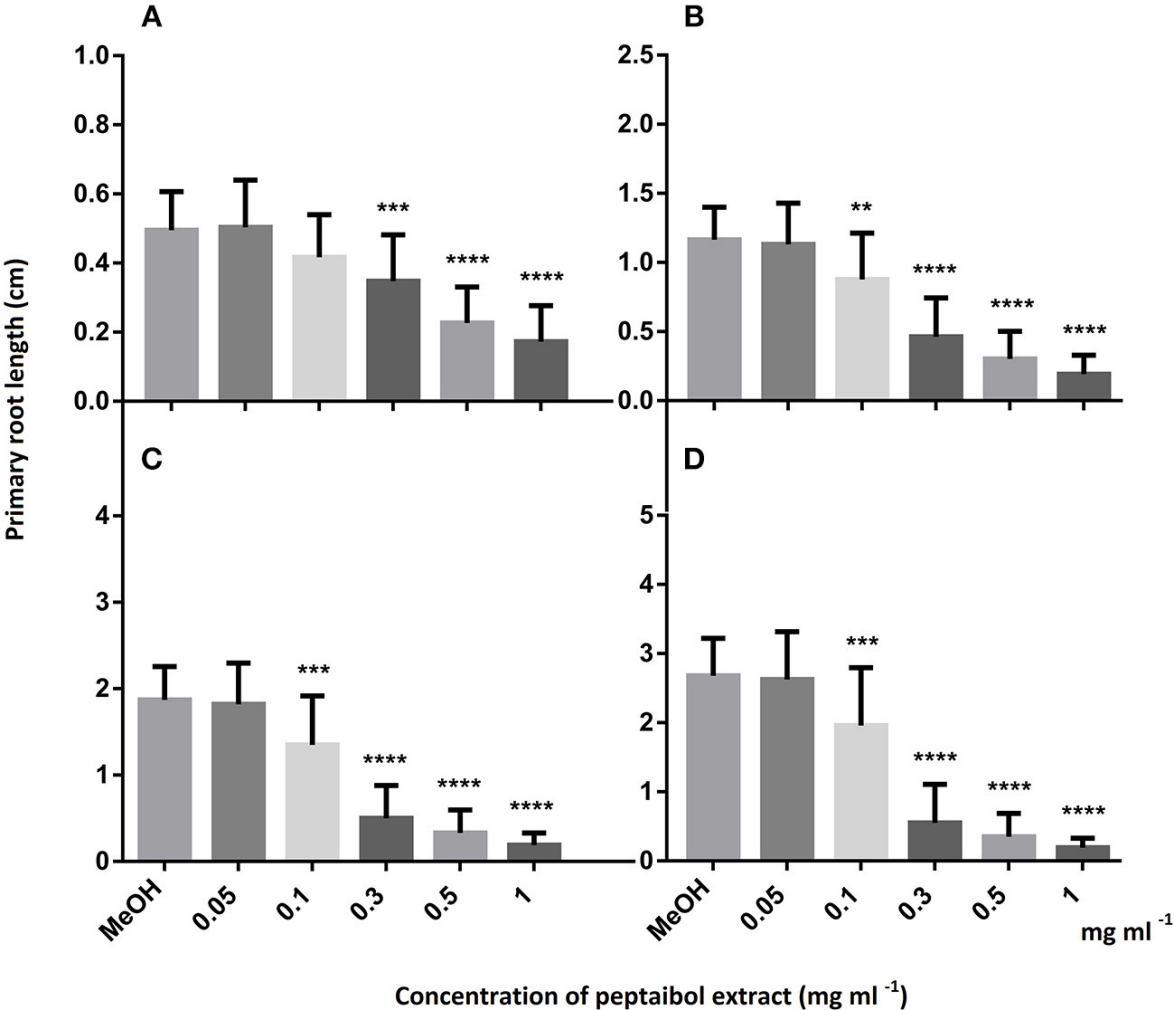
Primary root growth of 6 **(A)**, 7 **(B)**, 8 **(C)**, and 9 **(D)** days old *Arabidopsis thaliana* plants after treatment with peptaibol extract from *Trichoderma reesei* QM9414. Methanol was used for the control plants as all peptaibol extracts were prepared in this solvent. Significance is assessed based on *P*-values: ^*^P ≤ 0.05; ^**^P ≤ 0.01; ^***^P ≤ 0.001 and ^****^P ≤ 0.0001.

**Figure 6:**
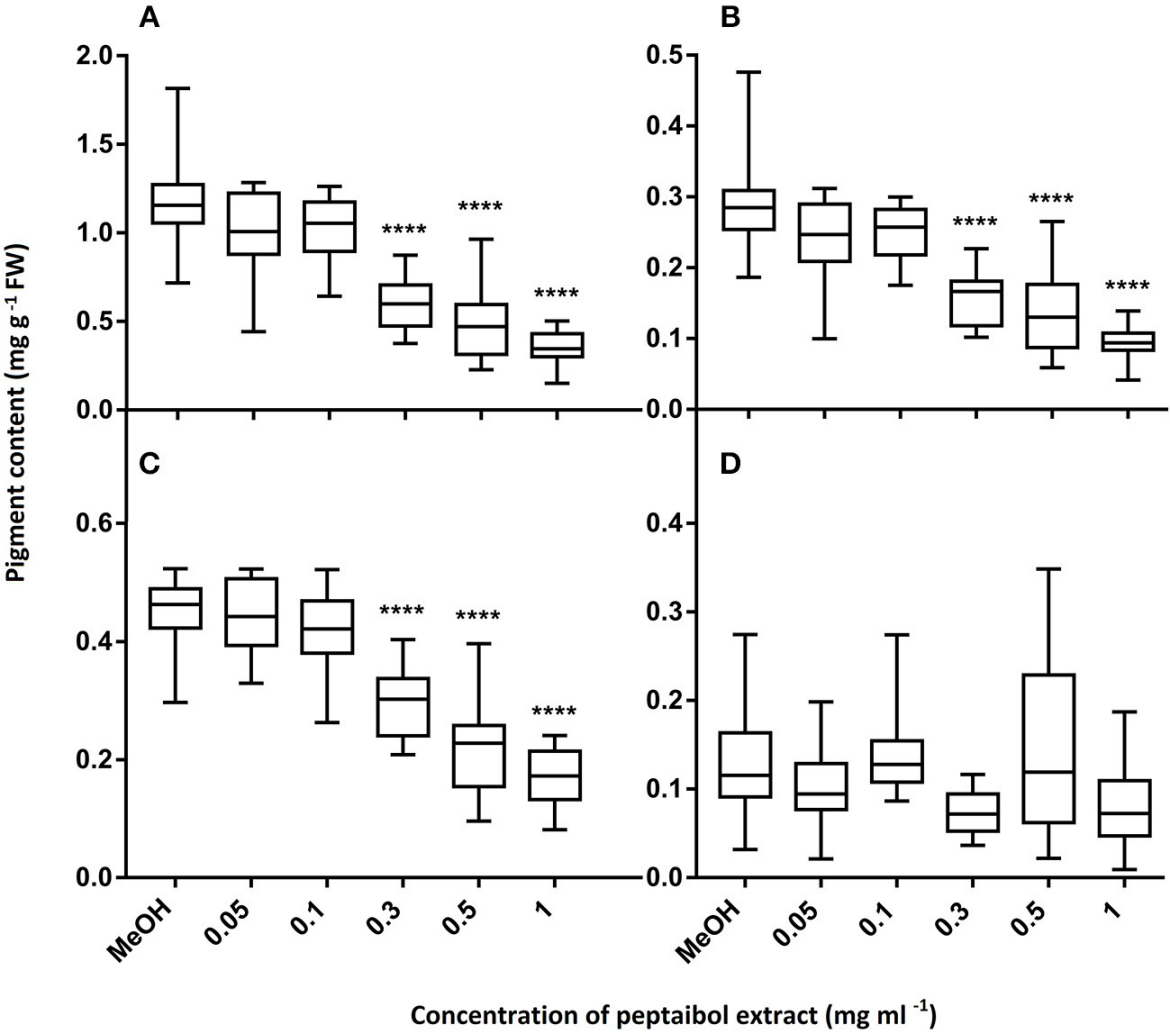
Pigment content of 15-day-old *Arabidopsis thaliana* leaves after treatment with peptaibol extract from *Trichoderma reesei* QM9414: chlorophyll-a **(A)**, chlorophyll-b **(B)**, carotenoids **(C)** and anthocyanins **(D)**. Methanol was used for the control plants. Significance is assessed based on *P*-values: ^*^P ≤ 0.05; ^**^P ≤ 0.01; ^***^P ≤ 0.001 and ^****^P ≤ 0.0001.

**Figure 7:**
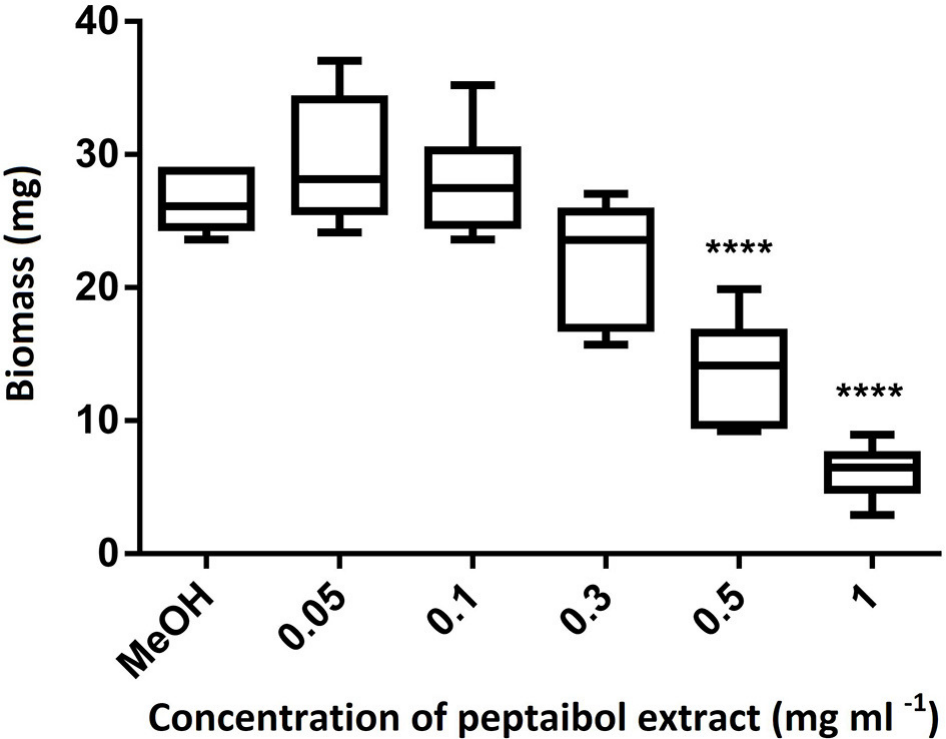
Biomass of 15-day-old *Arabidopsis thaliana* plants after treatment with peptaibol extract from *Trichoderma reesei* QM9414. Methanol was used for the control plants. Significance is assessed based on *P*-values: ^*^P ≤ 0.05; ^**^P ≤ 0.01; ^***^P ≤ 0.001 and ^****^P ≤ 0.0001.

In order to evaluate the value of peptaibols as antifungal agents for plant protection, the purified (98%) peptaibol extract of *T*. *reesei* QM9414 was investigated for toxicity in the model plant *A*. *thaliana*. The extract was diluted to 50, 10, 5, 1, 0.5, 0.3, 0.1, and 0.05 mg ml^−1^. All of the treated plants were inhibited after treatment with the peptaibol extract at concentrations of 50, 10, and 5 mg ml^−1^. Root growth was observed only at concentrations ≤ 1 mg ml^−1^; however, inhibited growth could be observed down to concentrations of 0.1 mg/ml (Figure 5). Treatment with 1 mg ml^−1^ peptaibol solution resulted in a hook formation of the primary roots. Chlorophyll-a, -b and carotenoid levels decreased after treatment with extracts of ≥0.3 mg ml^−1^ (Figure 6). Treatment with a peptaibol solution of 0.1 mg ml^−1^ resulted in a similar rate of production of photosynthetic pigments but an increased anthocyanin level in 15-day-old plants. The root growth of these plants was suppressed in 6- to 9-day-old plants, although the plants showed normal biomass and could probably eventually survive this minimal toxicity because of the increased levels of anthocyanin (Figure 7).

### Bioactivities of *T. reesei* Peptaibols on Mammalian Cells

The endpoint of toxic concentration—the last dilution step of the purified peptaibol solution which is toxic to mammalian cells—was determined for the peptaibol extract of *T*. *reesei* QM9414 (Table 7). After 20 min incubation at 37°C or 24 h at room temperature, the boar sperm motility inhibition end point was detected after treatment with 3 μg ml^−1^ peptaibol solution. The acrosome of the exposed sperm cells reacted at the same concentration, which inhibited motility, indicating that the toxic effect involves the plasma membrane. The inhibition end point of proliferation in porcine kidney PK-15 cells was observed at a concentration of 8 μg ml^−1^ peptaibol solution.

**Table 7:** Toxicity of the peptaibol extract from *T. reesei* QM9414 to boar sperm and porcine kidney cells.

	**EC_50_ (μg ml^−1^)**			
**Purified peptaibol extract**	**Sperm motility inhibition**		**Acrosome reaction**	**Inhibition of proliferation of Porcine kidney cells PK-15**
	**20 min**	**24 h**		
*T. reesei* QM9414	3	3	3	8
**REFRENCE SUBSTANCE**				
Alamethicin	5	0.2	0.2	8

*The values are the median of three measurements, represented by four microscopic fields. The variation between measurements was one dilution step*.

## Discussion

In this study, the structural diversity and bioactivity of peptaibol compounds produced by *Trichoderma* species belonging to the Longibrachiatum Clade were investigated and compared. The Longibrachiatum Clade is ecologically highly versatile as it contains both environmental and opportunistically pathogenic species, some of which can be found worldwide, whereas others are ecologically restricted. In total, 143 20-residue peptaibols could be identified from the 17 species examined, including 59 new and 76 recurrent compounds, as well as eight new 19-residue sequences. The peptaibols can be categorized into groups A, B and C, based on their primary structure, where groups A and B consist of 20-residue peptaibols, whereas group C is comprised exclusively of 19-residue sequences. The main difference between peptaibols of group A in relation to group B is in the R12 position. Sequence analysis identified several conserved regions along with some variable positions (R3, R5, R6, R10, R12, and R17), which have also been reported in a previous study (Pócsfalvi et al., 1997). Vxx was usually found instead of Ala and Aib at certain variable positions like R3, R5 and R6, which has never been observed among similar peptaibols. Although all of these amino acids have helix-forming properties, a substitution by Val would render a more linear and less fluctuating helical conformation owing to its bulkier sidechain. The highly curved backbone conformation is not energetically favored with increasing number of Val in peptaibol sequences. It has been hypothesized that the equilibrium between the bent (closed form) and linear conformations (open amphipathic form) may act as a “conformational switch” of voltage gating in ion channels across bilayers (North et al., 1995). Clearly, such substitutions have an important functional relevance, especially at subterminal positions like R3 and R17.

Brevicelsins from group C form a new family of 19-residue peptaibols similar to, but one amino acid shorter than group B sequences. They are not N-terminally truncated derivatives of their full-length precursors—like it is the case for the 16-residue brevikindins deriving from 18-residue trichokindin-like peptaibols (Degenkolb et al., 2016) – but differ from group B sequences by the internal deletion of position 6. This position is critical, since the following Gln plays an important role in the formation of ion channels (Wilson et al., 2011). Brevicelsins could be found only in three species: *T*. *flagellatum, T*. *sinense* and *T*. *parareesei*. A full genome sequence is available for *T. parareesei*, analysis of this sequence, however, revealed no extra 19-module NRPS synthetases but only a 20-module enzyme. The 19-residue peptaibols could be produced by the same, 20-module NRPS via the interaction of non-neighboring modules known as internal module skipping. The mechanisms of this phenomenon resulting in additional classes of 10-, 13-, 18-, and 19-residue peptaibols were proposed by Degenkolb et al. (2012). R6 is also skipped in *T*. *phellinicola* peptaibols (Röhrich et al., 2013), which does, however, contain Lxx in position R12, similar to group A peptaibols and unlike brevicelsins with Aib in this position.

The unique group A peptaibol profile of *T. novae-zelandiae* (Figure 1) may be related to the geographical origin of this species, which is endemic to New Zealand, and to its occupying a basal position in the Longibrachiatum Clade (Samuels et al., 2012). This species has tuberculate conidia, a trait also found in the Viride Clade (Jaklitsch et al., 2006), and it may be an ancestral trait of the Longibrachiatum Clade (Druzhinina et al., 2012). Our results suggest that the production of group A peptaibols may be another ancestral trait of the Longibrachiatum Clade, while the switch to the production of group B peptaibols might have occurred multiple times and seems therefore to be the result of convergent evolution. This switch from group A to group B has not fully completed in certain species: wild-type *T. reesei* as well as *T. saturnisporum* and *T. konilangbra* are also producing some group A compounds in addition to group B peptaibols.

Except from *T. reesei*, which was separated from its closest relative *T. parareesei*, the clustering based on peptaibol profiles reflected the close relationships within phylogenetic subclades in most of the cases (e.g., within subclades Longibrachiatum/Orientale, Citrinoviride/Pseudokoningii, or Konilangbra/Sinensis). For example, the species from the Konilangbra/Sinensis subclade are phylogenetically close to each other and are only known from the Paleotropical/Asian areas including Ethiopia (*T*. *flagellatum*), Uganda (*T*. *konilangbra*) and Taiwan (*T*. *sinensis*) (Samuels et al., 2012). The very close relationship of *T. sinensis* and *T. flagellatum* is also reflected by their ability to produce group C peptaibols in addition to group B sequences. The phylogenetic relationships between the subclades are less reflected by the clustering based on peptaibol profiles. Distantly related subclades (e.g., Longibrachiatum/Orientale and Citrinoviride/Pseudokoningii) may share similar profiles, while closely related subclades may exhibit substantially different ones—e.g., members of subclade Citrinoviride/Pseudokoningii produce group A peptaibols, while group B compounds are produced by their close relative *T. effusum*. This could be explained by multiple events of switching from the production of group A to group B during the evolution of the Longibrachiatum Clade.

Based on molecular dynamics simulations, 20-residue peptaibols result in higher linearity of helices than their 19-residue counterparts and are also relatively stable in terms of the atomic fluctuations of each residue. Paracelsins B, H and their 19-residue deletion sequences Brevicelsin I and IV all fold into right-handed helical structures with a slight bend at the Aib-Pro bond, except for Brevicelsin IV where the bend occurs at the Aib11-Aib12 bond. The Aib-Pro bond at R13-R14 in the case of 20-residue sequences is important for the secondary structure of the bent molecule. An important observation was made with respect to Val substitution instead of Aib at R17 which seems to hinder the formation of a bent backbone in close proximity to the N-terminal side-chains, because it is a chiral, hydrophobic amino acid with a bulkier side-chain than that of the achiral Aib. Frequent occurrence of Aib could be detected at the termini of the sequences, which are very important for the determination of the formation of helical structures including α- or 3_10_-helices (De Zotti et al., 2010; Gessmann et al., 2012a,b). The other promotor of the helical structure, D-Iva, is most often found close to the N-terminus, prior to the Gln-Aib bond in position R6, based on different previously described peptaibols such as boletusin 1, chrysospermins, peptaivirins, trichorzianins TA and TB, or the TA1938, 1924, 1910 and 1909a compounds (El-Hajji et al., 1987; Rebuffat et al., 1989; Dornberger et al., 1995; Lee et al., 1999; Yun et al., 2000; Panizel et al., 2013).

The growth of filamentous fungi pathogenic to plants or humans could be inhibited by the purified peptaibol extract of *T*. *reesei* QM9414. A stronger inhibition was observed in the case of the Δ*lae1* mutant of *T*. *reesei* than in the case of the other strains, suggesting that the mutation in the methyl transferase gene, which is known as a global epigenetic regulator of gene expression, may also affect tolerance to these metabolites. A previous study (Marik et al., 2018), in which crude peptaibol extracts were tested on several bacterial, yeast and filamentous fungal strains showed similar results. The inhibitory effects of peptaibols to bacteria and filamentous fungi have previously been reviewed (Szekeres et al., 2005; Daniel and Rodrigues Filho, 2007). It has also been demonstrated that purified trichokonin VI triggers a change of fungal membrane permeability and disintegration of subcellular structures, has an effect on mitochondrial membrane permeabilisation and intracellular ROS production, induces phosphatidylserine exposure and eventually triggers metacaspase-independent apoptosis in *F*. *oxysporum* (Shi et al., 2012).

Alamethicin, the most studied peptaibol was shown to induce resistance in plants (Leitgeb et al., 2007; Kredics et al., 2013), although it can also be toxic, causing lesions on *Arabidopsis* leaves (Rippa et al., 2010). At higher concentration, it induces rRNA cleavage-associated rapid death (Rippa et al., 2007). Alamethicin could permeabilise mainly the apical meristem and epidermis cells of the root tips, but not the basal meristem cells, cortex cells or the root cap of *A. thaliana* (Dotson et al., 2018). If the root was pretreated with cellulase, permeabilisation could not be observed. This study proved cellulose-induced resistance and cell-specific alamethicin permeabilisation of *A. thaliana* roots. Engelberth et al. (2001) successfully demonstrated the high biological activity of alamethicin that caused emission of volatile compounds from lima beans (*Phaseolus lunatus*) placed under low concentration of the peptaibol solution. When it was applied to *Bryonia dioica* tendrils at the same concentration, it elicited jasmonate-induced tendril coiling. Therefore, peptaibols may be used as potential elicitors of plant defense responses. Recently, antiviral activity of trichorzins was also reported on cowpea plants against *Cucumber mosaic virus* (Kai et al., 2018). In this recent study, bioactivity tests with the selected, purified peptaibol extract of *T*. *reesei* QM9414 demonstrated toxicity to *A*. *thaliana* plants at higher concentrations. An interesting effect of the peptaibol extract was the induction of hook formation in the root tips. A previous study revealed similar results, where the inoculation of *A*. *thaliana* with *T*. *atroviride* resulted in shortened primary root growth of the plants and ended in a hook formation, although the lateral root numbers were increased (Pelagio-Flores et al., 2017). An inhibitory effect on primary root growth in *A*. *thaliana* was also observed after interaction with *T*. *longibrachiatum* SMF2, and its peptaibols induced auxin production and disruption of the auxin response gradients in root tips (Shi et al., 2016).

Boar sperm cells are frequently used for the detection of toxins, which affect plasma membranes (Vicente-Carrillo, 2018; Castagnoli et al., 2018). Due to the high sensitivity of boar sperm cells to toxins, many studies have concluded that these tests are appropriate for toxin detection (Peltola et al., 2004; Andersson et al., 2009, 2010). Similar measurements of peptaibol extracts produced by *T*. *longibrachiatum* Thb have been reported, and a mixture of trilongins proved to be a stronger inhibitor of motility than trilongins alone, or any of the crude extracts (Mikkola et al., 2012). Single ion channels remained in an open state for a longer time when exposed to a combination of the long peptaibols (trilongins BI–BIV) with the short ones (trilongin AI), than for the long peptaibols alone. Furthermore, peptaibols (trichokonin VI) could inhibit HepG2 cancer cells by inducing autophagy and apoptosis through an influx of Ca^2+^, which triggered the activation of μ-calpain and proceeded to the translocation of Bax to mitochondria and the subsequent promotion of apoptosis (Shi et al., 2010). Another peptaibol, emericellipsin A, which is a short lipopeptaibol, exhibited selective cytotoxic activity against HepG2 and HeLa cell lines (Rogozhin et al., 2018), similar to culicinin D, another short linear peptaibol which has been described as a potent anticancer compound (He et al., 2006). In the present study, the partially purified peptaibol extract of *T*. *reesei* QM9414 proved to inhibit boar spermatozoa and porcine kidney PK-15 cells at 0.1 mg ml^−1^, which rises the question of a possible *in vivo* toxicity. Degenkolb et al. (2008) discussed this issue in detail and suggested that the toxicity of peptaibols may be well below the threshold of human consequence, and it may require direct contact with cell membranes, like in the case of common amphiphilic detergents. This is supported by previous observations demonstrating the very low toxicity of various peptaibols orally administered to rodents and ruminants (Hou et al., 1972; Nayar et al., 1973; Hino et al., 1994).

In conclusion, negative effects on *Arabidopsis* plants could not be detected below a certain concentration of the purified peptaibol extract from *T*. *reesei* QM9414, which could still inhibit plant pathogenic filamentous fungi. This observation suggests that purified peptaibol extracts may have potential value for plant protection. *T*. *reesei* is a well-characterized, widely used cellulase producer in the biotechnological industry, and so its peptaibols could be produced as the main product, or a valuable by-product of fermentation.

## Data Availability

All datasets generated for this study are included in the manuscript and/or the Supplementary Files.

## Author Contributions

LK, TM, AS, and CV designed the study and coordinated the draft of the manuscript. TM and DB took part in the extraction, HPLC separation, sequence determination, and antifungal activity testing of the peptaibol compounds. GE, DR, and AS conducted the mass spectrometry measurements. ID performed the sequence alignments and the comparative sequence analysis of peptaibol profiles. PU performed the annotation and bioinformatic analysis of NRPS gene clusters. CT contributed with the molecular dynamics simulations of peptaibols. TM, ÁS, and LB designed and performed the bioactivity tests on *A. thaliana*. MA and HS conducted the bioactivity assays on mammalian cells. TM, LK, and CV analyzed the results and designed the figures and tables. All authors read and approved the final manuscript.

### Conflict of Interest Statement

The authors declare that the research was conducted in the absence of any commercial or financial relationships that could be construed as a potential conflict of interest.
