# Corrigendum: Structural Diversity and Bioactivities of Peptaibol Compounds From the Longibrachiatum Clade of the Filamentous Fungal Genus *Trichoderma*

**DOI:** 10.3389/fmicb.2020.586868

**Published:** 2020-10-21

**Authors:** Tamás Marik, Chetna Tyagi, Dóra Balázs, Péter Urbán, Ágnes Szepesi, László Bakacsy, Gábor Endre, Dávid Rakk, András Szekeres, Maria A. Andersson, Heidi Salonen, Irina S. Druzhinina, Csaba Vágvölgyi, László Kredics

**Affiliations:** ^1^Department of Microbiology, Faculty of Science and Informatics, University of Szeged, Szeged, Hungary; ^2^Department of General and Environmental Microbiology, Faculty of Sciences, and Szentágothai Research Center, University of Pécs, Pécs, Hungary; ^3^Department of Plant Biology, Faculty of Science and Informatics, University of Szeged, Szeged, Hungary; ^4^Department of Civil Engineering, Aalto University, Espoo, Finland; ^5^Research Area Biochemical Technology, Institute of Chemical, Environmental and Bioscience Engineering, TU Wien, Vienna, Austria; ^6^Jiangsu Provincial Key Laboratory of Organic Solid Waste Utilization, Nanjing Agricultural University, Nanjing, China

**Keywords:** *Trichoderma*, Longibrachiatum, peptaibol, brevicelsin, mass spectrometry, antifungal activity, *Arabidopsis*, mammalian cells

In the original article, there was a mistake in the legend for Figure 4B as published. The root-mean-square deviation (in Å) calculated for each residue for all sequences gives an idea of the average fluctuation undergone by the system. The 19-residue brevicelsins show higher fluctuation than their 20-residue paracelsin counterparts. The correct legend appears below.

**Figure 4:**
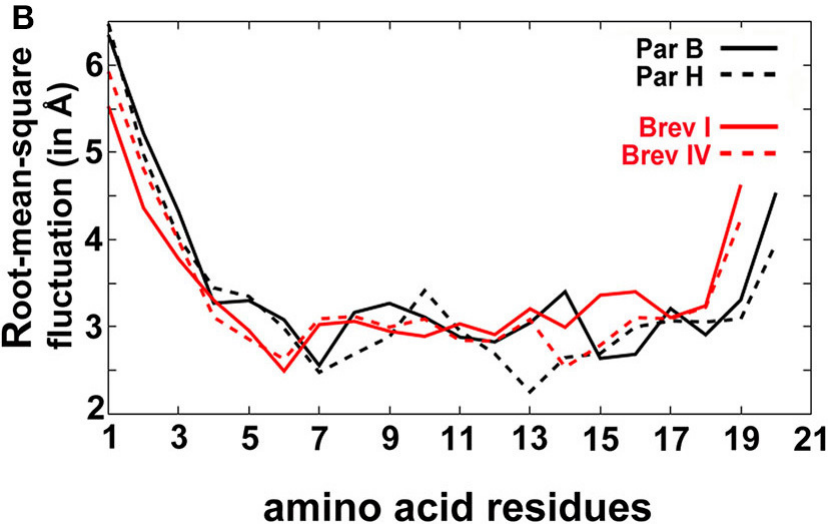
**(B)** The root-mean-square deviation (in Å) calculated for each residue for all sequences gives an idea of the average fluctuation undergone by the system.

Figure 4B. The root-mean-square deviation (in Å) calculated for each residue for all sequences gives an idea of the average fluctuation undergone by the system.

In the original article, there was a mistake in Figure 4B as published. The root-mean-square atomic fluctuation values were calculated without the root-mean-square fitting of the simulation trajectory with an average structure which removes the effect of global motions. The corrected Figure 4 appears below.

In the original article, there was an error in the section **Structural Characterization of 20- and 19-Residue Peptaibols**, Paragraph 2:

“The root-mean-square-atomic fluctuation (RMSF) graph (Figure 4B) shows higher fluctuation in the N- and C-terminal regions of all peptides in comparison with their central regions. However, the most significant observation is that there is considerably higher atomic fluctuation of the 19-residue peptaibols Brevicelsin I and IV in comparison to the 20-residue peptaibols Paracelsin B and H. It seems that the loss of one residue, resulting in a shorter sequence, results in higher atomic fluctuations, whereas longer peptaibols are comparatively more stable. In all four sequences, a small but sharp spike in the RMSF value of Gln at R6 of the 19-residue peptaibols and R7 of the 20-residue peptaibols reinforces the importance of glutamines in channel formation and stabilization (Whitmore and Wallace, 2004). Aib17 has higher average atomic fluctuation than Val17, due to its tendency to oscillate between right- and left-handed helical forms, whereas Val17 takes a rigid conformation.”

A correction has been made to **Structural Characterization of 20- and 19-Residue Peptaibols**, Paragraph 2:

“The root-mean-square-atomic fluctuation (RMSF) graph (Figure 4B) shows higher fluctuation of N-terminus region for all peptides. No other significant differences were observed between the RMSF values of the 19-residue peptaibols, Brevicelsins I and IV, in comparison to 20-residue peptaibols, Paracelsins B and H, except that the sequences containing more Aib residues show a slight elevation in atomic fluctuation at the corresponding sequence position. For example, at R16 for Brevicelsin I and R17 for Paracelsin B, also, the R6 Aib in Paracelsins B and H shows higher average atomic fluctuation than the R6 Gln of Brevicelsins I and IV. This observation establishes the fluctuating and dynamic nature of the Aib residue in peptaibol sequences which can be explained by its tendency to oscillate between right- and left-handed helical forms. The Gln residues at R7 and R6 positions of paracelsins and brevicelsins, respectively, show a sharp dip in atomic fluctuation indicating higher stability in comparison to the C-terminal Gln residues and highlights importance of glutamines in ion-channel stabilization (Whitmore and Wallace, 2004).”

The authors apologize for this error and state that this does not change the scientific conclusions of the article. The original article has been updated.
