# Supplementary material for: Structural Diversity and Bioactivities of Peptaibol Compounds From the Longibrachiatum Clade of the Filamentous Fungal Genus Trichoderma
Source: Front Microbiol. 2019 Jun 26;10:1434. doi: 10.3389/fmicb.2019.01434 (PMC6606783; doi:10.3389/fmicb.2019.01434)
Supplement: Supplementary file 1 [file Data_Sheet_1.docx]

**Supplementary Table 1 ǀ** Diagnostic fragment ions of peptaibols detected with the full scan MS measurement of peptaibol compounds from group A

| **Peptide** | **M** | **[M+H]^+^** | **b_1_** | **b_2_** | **b_3_** | **b_4_** | **b_5_** | **b_6_** | **b_7_** | **b_8_** | **b_9_** | **b_10_** | **b_11_** | **b_12_** | **b_13_** | **y_7_** |
| --- | --- | --- | --- | --- | --- | --- | --- | --- | --- | --- | --- | --- | --- | --- | --- | --- |
| Pept-A-Ia | 1922.0989 | 1923.1048 | 128.0704 | 199.1071 | 284.1598 | 355.1985 | 440.2528 | 511.2871 | n.d. | 724.3967 | 823.4642 | 894.5065 | 951.5278 | 1064.6081 | 1149.6611 | 774.4469 |
| Pept-A-Ib | 1922.0989 | 1923.1048 | 128.0704 | 199.1071 | 284.1598 | 355.1985 | 440.2528 | 525.3029 | n.d. | 738.4129 | 837.4860 | 908.5167 | 965.5449 | 1064.6081 | 1149.6611 | 774.4469 |
| Pept-A-IIa | 1923.0829 | 1924.0999 | 128.0704 | 199.1071 | 284.1598 | 355.1985 | 440.2528 | 511.2871 | n.d. | 724.3967 | 823.4642 | 908.5167 | 965.5449 | 1064.6081 | 1149.6611 | 775.4322 |
| Pept-A-IIb | 1923.0829 | 1924.0999 | 128.0704 | 199.1071 | 284.1598 | 355.1985 | 440.2528 | 511.2871 | n.d. | 724.3967 | 823.4642 | 894.5065 | 951.5278 | 1064.6081 | 1149.6611 | 775.4322 |
| Pept-A-IIIa | 1936.1146 | 1937.1206 | 128.0704 | 199.1071 | 284.1598 | 355.1985 | 440.2528 | 511.2871 | n.d. | 724.3967 | 823.4642 | 908.5167 | 965.5449 | 1064.6081 | 1149.6611 | 788.4616 |
| Pept-A-IIIb | 1936.1146 | 1937.1206 | 128.0704 | 199.1071 | 284.1598 | 355.1985 | 440.2528 | 511.2871 | n.d. | 724.3967 | 823.4642 | 894.5065 | 951.5278 | 1064.6081 | 1149.6611 | 788.4616 |
| Pept-A-IIIc | 1936.1146 | 1937.1206 | 128.0704 | 199.1071 | 284.1598 | 355.1985 | 440.2528 | 525.3029 | n.d. | 738.4129 | 837.4860 | 908.5167 | 965.5449 | 1064.6081 | 1149.6611 | 788.4616 |
| Pept-A-IVa | 1936.1146 | 1937.1206 | 128.0704 | 199.1071 | 284.1598 | 355.1985 | 440.2528 | 511.2871 | n.d. | 724.3967 | 823.4642 | 908.5167 | 965.5449 | 1078.6224 | 1163.6769 | 774.4469 |
| Pept-A-IVb | 1936.1146 | 1937.1206 | 128.0704 | 199.1071 | 284.1598 | 355.1985 | 440.2528 | 511.2871 | n.d. | 724.3967 | 837.4860 | 908.5167 | 965.5449 | 1078.6224 | 1163.6769 | 774.4469 |
| Pept-A-Va | 1950.1302 | 1951.1368 | 128.0704 | 199.1071 | 284.1598 | 355.1985 | 440.2528 | 525.3029 | n.d. | 738.4129 | 837.4860 | 922.5319 | 979.5613 | 1092.6402 | 1177.6921 | 774.4469 |
| Pept-A-Vb | 1950.1302 | 1951.1368 | 128.0704 | 199.1071 | 284.1598 | 355.1985 | 440.2528 | 511.2871 | n.d. | 724.3967 | 837.4860 | 922.5319 | 979.5613 | 1092.6402 | 1177.6921 | 774.4469 |
| Pept-A-VIa | 1937.0986 | 1938.0984 | 128.0704 | 199.1071 | 284.1598 | 355.1985 | 440.2528 | 511.2871 | n.d. | 724.3967 | 823.4642 | 908.5167 | 965.5449 | 1078.6224 | 1163.6769 | 775.4322 |
| Pept-A-VIb | 1937.0986 | 1938.0984 | 128.0704 | 199.1071 | 284.1598 | 355.1985 | 440.2528 | 511.2871 | n.d. | 724.3967 | 837.4860 | 908.5167 | 965.5449 | 1078.6224 | 1163.6769 | 775.4322 |
| Pept-A-VIIa | 1936.1146 | 1937.1206 | 128.0704 | 199.1071 | 284.1598 | 355.1985 | 440.2528 | 511.2871 | n.d. | 724.3967 | 823.4642 | 908.5167 | 965.5449 | 1078.6224 | 1163.6769 | 774.4469 |
| Pept-A-VIIb | 1936.1146 | 1937.1206 | 128.0704 | 199.1071 | 298.1597 | 369.2134 | 454.2668 | 525.3029 | n.d. | 738.4129 | 837.4860 | 908.5167 | 965.5449 | 1078.6224 | 1163.6769 | 774.4469 |
| Pept-A-VIIIa | 1950.1302 | 1951.1368 | 128.0704 | 199.1071 | 298.1597 | 369.2134 | 454.2668 | 525.3029 | n.d. | 738.4129 | 851.4953 | 922.5319 | 979.5613 | 1092.6402 | 1177.6921 | 774.4469 |
| Pept-A-VIIIb | 1950.1302 | 1951.1368 | 128.0704 | 199.1071 | 298.1597 | 369.2134 | 454.2668 | 525.3029 | n.d. | 738.4129 | 837.4860 | 922.5319 | 979.5613 | 1092.6402 | 1177.6921 | 774.4469 |
| Pept-A-IXa | 1950.1302 | 1951.1368 | 128.0704 | 199.1071 | 284.1598 | 355.1985 | 440.2528 | 511.2871 | n.d. | 724.3967 | 823.4642 | 908.5167 | 965.5449 | 1078.6224 | 1163.6769 | 788.4616 |
| Pept-A-IXb | 1950.1302 | 1951.1368 | 128.0704 | 199.1071 | 284.1598 | 355.1985 | 440.2528 | 511.2871 | n.d. | 724.3967 | 837.4860 | 908.5167 | 965.5449 | 1078.6224 | 1163.6769 | 788.4616 |
| Pept-A-Xa | 1964.1459 | 1965.1522 | 128.0704 | 199.1071 | 284.1598 | 355.1985 | 440.2528 | 525.3029 | n.d. | 738.4129 | 837.4860 | 922.5319 | 979.5613 | 1092.6402 | 1177.6921 | 788.4616 |
| Pept-A-Xb | 1964.1459 | 1965.1522 | 128.0704 | 199.1071 | 284.1598 | 355.1985 | 440.2528 | 511.2871 | n.d. | 724.3967 | 837.4860 | 922.5319 | 979.5613 | 1092.6402 | 1177.6921 | 788.4616 |
| Pept-A-XIa | 1951.1143 | 1952.1302 | 128.0704 | 199.1071 | 298.1597 | 369.2134 | 454.2668 | 525.3029 | n.d. | 738.4129 | 837.4860 | 922.5319 | 979.5613 | 1092.6402 | 1177.6921 | 775.4322 |
| Pept-A-XIb | 1951.1143 | 1952.1302 | 128.0704 | 199.1071 | 284.1598 | 355.1985 | 440.2528 | 525.3029 | n.d. | 738.4129 | 837.4860 | 922.5319 | 979.5613 | 1092.6402 | 1177.6921 | 775.4322 |
| Pept-A-XIc | 1951.1143 | 1952.1302 | 128.0704 | 199.1071 | 298.1597 | 369.2134 | 454.2668 | 525.3029 | n.d. | 738.4129 | 851.4953 | 922.5319 | 979.5613 | 1092.6402 | 1177.6921 | 775.4322 |
| Pept-A-XII | 1937.0986 | 1938.0984 | 128.0704 | 199.1071 | 284.1598 | 355.1985 | 440.2528 | 511.2871 | n.d. | 724.3967 | 837.4860 | 908.5167 | 965.5449 | 1078.6224 | 1163.6769 | 775.4322 |

n.d.: not detected

**Supplementary Table 1 ǀ** continued

| **Peptide** | **M** | **[M+H]^+^** | **b_1_** | **b_2_** | **b_3_** | **b_4_** | **b_5_** | **b_6_** | **b_7_** | **b_8_** | **b_9_** | **b_10_** | **b_11_** | **b_12_** | **b_13_** | **y_7_** |
| --- | --- | --- | --- | --- | --- | --- | --- | --- | --- | --- | --- | --- | --- | --- | --- | --- |
| Pept-A-XIIIa | 1951.1143 | 1952.1302 | 128.0704 | 199.1071 | 284.1598 | 355.1985 | 440.2528 | 511.2871 | n.d. | 724.3967 | 823.4642 | 908.5167 | 965.5449 | 1078.6224 | 1163.6769 | 789.4509 |
| Pept-A-XIIIb | 1951.1143 | 1952.1302 | 128.0704 | 199.1071 | 284.1598 | 355.1985 | 440.2528 | 511.2871 | n.d. | 724.3967 | 837.4860 | 908.5167 | 965.5449 | 1078.6224 | 1163.6769 | 789.4509 |
| Pept-A-XIVa | 1965.1299 | 1966.1444 | 128.0704 | 199.1071 | 284.1598 | 355.1985 | 440.2528 | 525.3029 | n.d. | 738.4129 | 851.4953 | 922.5319 | 979.5613 | 1092.6402 | 1177.6921 | 789.4509 |
| Pept-A- XIVb | 1964.1459 | 1965.1522 | 128.0704 | 199.1071 | 284.1598 | 355.1985 | 440.2528 | 525.3029 | n.d. | 738.4129 | 837.4860 | 922.5319 | 979.5613 | 1092.6402 | 1177.6921 | 788.4616 |
| Pept-A-XVa | 1950.1302 | 1951.1368 | 128.0704 | 199.1071 | 284.1598 | 355.1985 | 440.2528 | 511.2871 | n.d. | 724.3967 | 823.4642 | 908.5167 | 965.5449 | 1078.6224 | 1163.6769 | 788.4616 |
| Pept-A-XVb | 1964.1459 | 1965.1522 | 128.0704 | 199.1071 | 284.1598 | 355.1985 | 440.2528 | 525.3029 | n.d. | 738.4129 | 851.4953 | 922.5319 | 979.5613 | 1092.6402 | 1177.6921 | 788.4616 |
| Pept-A-XVIa | 1950.1302 | 1951.1368 | 128.0704 | 199.1071 | 284.1598 | 355.1985 | 440.2528 | 525.3029 | n.d. | 738.4129 | 837.4860 | 922.5319 | 979.5613 | 1092.6402 | 1177.6921 | 774.4469 |
| Pept-A-XVIb | 1950.1302 | 1951.1368 | 128.0704 | 199.1071 | 284.1598 | 355.1985 | 440.2528 | 525.3029 | n.d. | 738.4129 | 851.4953 | 922.5319 | 979.5613 | 1092.6402 | 1177.6921 | 774.4469 |
| Pept-A-XVIIa | 1964.1459 | 1965.1522 | 128.0704 | 199.1071 | 284.1598 | 355.1985 | 440.2528 | 525.3029 | n.d. | 738.4129 | 837.4860 | 922.5319 | 979.5613 | 1092.6402 | 1177.6921 | 788.4616 |
| Pept-A-XVIIb | 1950.1302 | 1951.1368 | 128.0704 | 199.1071 | 284.1598 | 355.1985 | 440.2528 | 511.2871 | n.d. | 724.3967 | 823.4642 | 908.5167 | 965.5449 | 1078.6224 | 1163.6769 | 788.4616 |
| Pept-A-XVIII | 1978.1615 | 1979.1713 | 128.0704 | 199.1071 | 298.1597 | 369.2134 | 454.2668 | 539.3186 | n.d. | 752.4301 | 851.4953 | 936.5563 | 993.5684 | 1106.6547 | 1191.7080 | 788.4616 |
| Pept-A-XIXa | 1951.1143 | 1952.1302 | 128.0704 | 199.1071 | 284.1598 | 355.1985 | 440.2528 | 525.3029 | n.d. | 738.4129 | 837.4860 | 922.5319 | 979.5613 | 1092.6402 | 1177.6921 | 775.4322 |
| Pept-A-XIXb | 1951.1143 | 1952.1302 | 128.0704 | 199.1071 | 284.1598 | 355.1985 | 440.2528 | 525.3029 | n.d. | 738.4129 | 851.4953 | 922.5319 | 979.5613 | 1092.6402 | 1177.6921 | 775.4322 |
| Pept-A-XX | 1964.1459 | 1965.1522 | 128.0704 | 199.1071 | 298.1597 | 369.2134 | 454.2668 | 539.3186 | n.d. | 752.4301 | 851.4953 | 936.5563 | 993.5867 | 1106.6547 | 1191.7080 | 774.4469 |
| Pept-A-XXIa | 1964.1459 | 1965.1522 | 128.0704 | 199.1071 | 284.1598 | 355.1985 | 440.2528 | 525.3029 | n.d. | 738.4129 | 837.4860 | 922.5319 | 979.5613 | 1092.6402 | 1177.6921 | 788.4616 |
| Pept-A-XXIb | 1964.1459 | 1965.1522 | 128.0704 | 199.1071 | 284.1598 | 355.1985 | 440.2528 | 525.3029 | n.d. | 738.4129 | 851.4953 | 922.5319 | 979.5613 | 1092.6402 | 1177.6921 | 788.4616 |
| Pept-A-XXIIa | 1964.1459 | 1965.1522 | 128.0704 | 199.1071 | 298.1597 | 369.2134 | 454.2668 | 539.3186 | n.d. | 752.4301 | 851.4953 | 936.5563 | 993.5684 | 1106.6547 | 1191.7080 | 774.4469 |
| Pept-A-XXIIb | 1964.1459 | 1965.1522 | 128.0704 | 199.1071 | 284.1598 | 355.1985 | 440.2528 | 525.3029 | n.d. | 738.4129 | 851.4953 | 936.5563 | 993.5684 | 1106.6547 | 1191.7080 | 774.4469 |
| Pept-A-XXIII | 1965.1299 | 1966.1444 | 128.0704 | 199.1071 | 284.1598 | 355.1985 | 440.2528 | 525.3029 | n.d. | 738.4129 | 837.4860 | 922.5319 | 979.5613 | 1092.6402 | 1177.6921 | 789.4509 |
| Pept-A-XXIV | 1978.1615 | 1979.1713 | 128.0706 | 199.1072 | 298.1597 | 369.2134 | 454.2668 | 539.3186 | n.d. | 752.4301 | 851.4953 | 936.5563 | 993.5684 | 1106.6547 | 1191.7080 | 788.4616 |
| Pept-A-XXVa | 1964.1459 | 1965.1522 | 128.0704 | 199.1071 | 284.1598 | 355.1985 | 440.2528 | 525.3029 | n.d. | 738.4129 | 837.4860 | 922.5319 | 979.5613 | 1092.6402 | 1177.6921 | 788.4616 |
| Pept-A-XXVb | 1978.1615 | 1979.1713 | 128.0704 | 199.1071 | 284.1598 | 355.1985 | 440.2528 | 525.3029 | n.d. | 738.4129 | 851.4953 | 936.5563 | 993.5684 | 1106.6547 | 1191.7080 | 788.4616 |
| Pept-A-XXVIa | 1978.1615 | 1979.1713 | 128.0704 | 199.1071 | 298.1597 | 369.2134 | 454.2668 | 539.3186 | n.d. | 752.4301 | 851.4953 | 936.5563 | 993.5684 | 1106.6547 | 1191.7080 | 788.4616 |
| Pept-A-XXVIb | 1978.1615 | 1979.1713 | 128.0704 | 199.1071 | 284.1598 | 355.1985 | 440.2528 | 525.3029 | n.d. | 738.4129 | 851.4953 | 936.5563 | 993.5684 | 1106.6547 | 1191.7080 | 788.4616 |
| Pept-A-XXVIIa | 1965.1299 | 1966.1444 | 128.0704 | 199.1071 | 284.1598 | 355.1985 | 440.2528 | 525.3029 | n.d. | 738.4129 | 837.4860 | 922.5319 | 979.5613 | 1092.6402 | 1177.6921 | 789.4509 |
| Pept-A-XXVIIb | 1978.1615 | 1979.1713 | 128.0704 | 199.1071 | 284.1598 | 355.1985 | 440.2528 | 525.3029 | n.d. | 738.4129 | 851.4953 | 936.5563 | 993.5684 | 1106.6547 | 1191.7080 | 788.4616 |

n.d.: not detected

**Supplementary Table 2 ǀ** Diagnostic fragment ions of peptaibols detected with the full scan MS measurement of peptaibol compounds from group B

| **Peptide** | **M** | **[M+H]^+^** | **b_1_** | **b_2_** | **b_3_** | **b_4_** | **b_5_** | **b_6_** | **b_7_** | **b_8_** | **b_9_** | **b_10_** | **b_11_** | **b_12_** | **b_13_** | **y_7_** |
| --- | --- | --- | --- | --- | --- | --- | --- | --- | --- | --- | --- | --- | --- | --- | --- | --- |
| Pept-B-I | 1908.0833 | 1909.0897 | 128.0704 | 199.1071 | 270.1443 | 341.1819 | 426.2344 | 497.2745 | n.d. | 710.3871 | 823.4642 | 908.5167 | 965.5449 | 1050.5927 | 1135.6458 | 774.4469 |
| Pept-B-II | 1908.0833 | 1909.0897 | 128.0704 | 199.1071 | 270.1443 | 341.1819 | 426.2344 | 497.2745 | n.d. | 710.3871 | 823.4642 | 908.5167 | 965.5449 | 1050.5927 | 1135.6458 | 774.4469 |
| Pept-B-III | 1908.0833 | 1909.0897 | 128.0704 | 199.1071 | 270.1443 | 341.1819 | 426.2344 | 497.2745 | n.d. | 710.3871 | 823.4642 | 908.5167 | 965.5449 | 1050.5927 | 1135.6458 | 774.4469 |
| Pept-B-IV | 1922.0989 | 1923.1048 | 128.0704 | 199.1071 | 270.1443 | 341.1819 | 426.2344 | 497.2745 | n.d. | 710.3871 | 823.4642 | 908.5167 | 965.5449 | 1050.5927 | 1135.6458 | 788.4616 |
| Pept-B-V | 1908.0833 | 1909.0897 | 128.0704 | 199.1071 | 270.1443 | 341.1819 | 426.2344 | 497.2745 | n.d. | 710.4143 | 823.4719 | 908.5167 | 965.5449 | 1050.5927 | 1135.6458 | 774.4469 |
| Pept-B-VI | 1922.0989 | 1923.1048 | 128.0704 | 199.1071 | 284.1598 | 355.1985 | 440.2505 | 511.2871 | n.d. | 724.3967 | 837.4860 | 922.5308 | 979.5613 | 1064.6081 | 1149.6611 | 774.4469 |
| Pept-B-VII | 1922.0989 | 1923.1048 | 128.0704 | 199.1071 | 270.1443 | 341.1819 | 426.2344 | 497.2745 | n.d. | 710.3871 | 823.4642 | 908.5167 | 965.5449 | 1050.5927 | 1135.6458 | 788.4616 |
| Pept-B-VIII | 1936.1146 | 1937.1206 | 128.0704 | 199.1071 | 284.1598 | 355.1985 | 440.2528 | 511.2871 | n.d. | 724.3967 | 837.4860 | 922.5319 | 979.5613 | 1064.6081 | 1149.6611 | 788.4616 |
| Pept-B-IXa | 1908.0833 | 1909.0897 | 128.0704 | 199.1071 | 284.1598 | 355.1985 | 440.2528 | 511.2871 | n.d. | 724.3967 | 823.4642 | 908.5167 | 965.5449 | 1050.5927 | 1135.6458 | 774.4469 |
| Pept-B-IXb | 1909.0986 | 1909.0897 | 128.0704 | 199.1071 | 284.1598 | 355.1985 | 440.2528 | 511.2871 | n.d. | 724.3967 | 837.4860 | 922.5319 | 979.5613 | 1050.5927 | 1135.6458 | 774.4469 |
| Pept-B-X | 1922.0989 | 1923.1048 | 128.0704 | 199.1071 | 284.1598 | 355.1985 | 440.2528 | 511.2871 | n.d. | 724.3967 | 823.4642 | 908.5167 | 965.5449 | 1050.5927 | 1135.6458 | 788.4616 |
| Pept-B-XI | 1922.0989 | 1923.1048 | 128.0704 | 199.1071 | 284.1598 | 355.1985 | 440.2528 | 511.2871 | n.d. | 724.3967 | 823.4642 | 908.5167 | 965.5449 | 1050.5927 | 1135.6458 | 788.4616 |
| Pept-B-XII | 1922.0989 | 1923.1048 | 128.0704 | 199.1071 | 284.1598 | 355.1985 | 440.2528 | 511.2871 | n.d. | 724.3967 | 837.4860 | 922.5319 | 979.5613 | 1064.6081 | 1149.6611 | 774.4469 |
| Pept-B-XIII | 1922.0989 | 1923.1048 | 128.0704 | 199.1071 | 284.1598 | 355.1985 | 440.2528 | 511.2871 | n.d. | 724.3967 | 823.4642 | 908.5167 | 965.5449 | 1050.5927 | 1135.6458 | 788.4616 |
| Pept-B-XIVa | 1923.0829 | 1924.0999 | 128.0704 | 199.1071 | 284.1598 | 355.1985 | 440.2528 | 511.2871 | n.d. | 724.3967 | 837.4860 | 922.5319 | 979.5613 | 1064.6081 | 1149.6611 | 775.4322 |
| Pept-B-XIVb | 1923.0829 | 1924.0999 | 128.0704 | 199.1071 | 284.1598 | 355.1985 | 440.2528 | 525.3029 | n.d. | 738.4078 | 837.4860 | 922.5319 | 979.5613 | 1064.6081 | 1149.6611 | 775.4322 |
| Pept-B-XVa | 1922.0989 | 1923.1048 | 128.0704 | 199.1071 | 284.1598 | 355.1985 | 440.2528 | 511.2871 | n.d. | 724.3967 | 823.4642 | 908.5167 | 965.5449 | 1050.5927 | 1135.6458 | 788.4616 |
| Pept-B-XVb | 1922.0989 | 1923.1048 | 128.0704 | 199.1071 | 284.1598 | 355.1985 | 440.2528 | 511.2871 | n.d. | 724.3967 | 837.4860 | 922.5319 | 979.5613 | 1050.5927 | 1135.6458 | 788.4616 |
| Pept-B-XVI | 1922.0989 | 1923.1048 | 128.0704 | 199.1071 | 284.1598 | 355.1985 | 440.2528 | 525.3029 | n.d. | 738.4129 | 837.4860 | 922.5319 | 979.5613 | 1064.6081 | 1149.6611 | 774.4469 |
| Pept-B-XVII | 1936.1146 | 1937.1206 | 128.0704 | 199.1071 | 284.1598 | 355.1985 | 440.2528 | 511.2871 | n.d. | 724.3967 | 837.4860 | 922.5319 | 979.5613 | 1064.6081 | 1149.6611 | 788.4616 |
| Pept-B-XVIII | 1923.1067 | 1924.0999 | 128.0704 | 199.1071 | 284.1598 | 355.1985 | 440.2528 | 511.2871 | n.d. | 724.3967 | 837.4860 | 922.5319 | 979.5613 | 1064.6081 | 1149.6611 | 774.4469 |
| Pept-B-XIX | 1936.1146 | 1937.1206 | 128.0704 | 199.1071 | 284.1598 | 355.1985 | 440.2528 | 511.2871 | n.d. | 724.3967 | 837.4860 | 922.5319 | 979.5613 | 1064.6081 | 1149.6611 | 788.4616 |
| Pept-B-XX | 1936.1146 | 1937.1206 | 128.0704 | 199.1071 | 284.1598 | 355.1985 | 440.2528 | 525.3029 | n.d. | 738.4129 | 851.4953 | 936.5465 | 993.5684 | 1078.6224 | 1163.6769 | 774.4469 |
| Pept-B-XXI | 1937.0986 | 1938.0984 | 128.0704 | 199.1071 | 284.1598 | 355.1985 | 440.2528 | 511.2871 | n.d. | 724.3967 | 837.4860 | 922.5319 | 979.5613 | 1064.6081 | 1149.6611 | 789.4509 |
| Pept-B-XXII | 1936.1146 | 1937.1206 | 128.0704 | 199.1071 | 284.1598 | 355.1985 | 440.2528 | 511.2871 | n.d. | 724.3967 | 837.4860 | 922.5319 | 979.5613 | 1064.6081 | 1149.6611 | 788.4616 |
| Pept-B-XXIII | 1922.0989 | 1923.1048 | 128.0704 | 199.1071 | 284.1598 | 355.1985 | 440.2528 | 511.2871 | n.d. | 724.3967 | 837.4860 | 922.5319 | 979.5613 | 1064.6081 | 1149.6611 | 774.4469 |
| Pept-B-XXIV | 1950.1302 | 1951.1368 | 128.0704 | 199.1071 | 284.1598 | 355.1985 | 440.2528 | 525.3029 | n.d. | 738.4129 | 851.5267 | 936.5563 | 993.5684 | 1078.6224 | 1163.6755 | 788.4616 |
| Pept-B-XXV | 1937.0986 | 1938.0984 | 128.0704 | 199.1071 | 284.1598 | 355.1985 | 440.2528 | 525.3029 | n.d. | 738.4129 | 851.4953 | 936.5563 | 993.5684 | 1078.6224 | 1163.6769 | 775.4322 |
| Pept-B-XXVI | 1950.1302 | 1951.1368 | 128.0704 | 199.1071 | 298.1756 | 369.2134 | 454.2668 | 539.3186 | n.d. | 752.4368 | 865.5170 | 950.5699 | 1007.5874 | 1092.6402 | 1177.6921 | 774.4469 |

n.d.: not detected

**Supplementary Table 2 ǀ** continued

| **Peptide** | **M** | **[M+H]^+^** | **b_1_** | **b_2_** | **b_3_** | **b_4_** | **b_5_** | **b_6_** | **b_7_** | **b_8_** | **b_9_** | **b_10_** | **b_11_** | **b_12_** | **b_13_** | **y_7_** |
| --- | --- | --- | --- | --- | --- | --- | --- | --- | --- | --- | --- | --- | --- | --- | --- | --- |
| Pept-B-XXVII | 1950.1302 | 1951.1368 | 128.0704 | 199.1071 | 284.1598 | 355.1985 | 440.2528 | 525.3029 | n.d. | 738.4129 | 851.4953 | 936.5563 | 993.5684 | 1078.6224 | 1163.6769 | 788.4616 |
| Pept-B-XXVIII | 1950.1302 | 1951.1368 | 128.0704 | 199.1071 | 284.1598 | 355.1985 | 454.2668 | 539.3186 | n.d. | 752.4301 | 865.5170 | 950.5699 | 1007.5874 | 1092.6402 | 1177.6921 | 774.4469 |
| Pept-B-XXIXa | 1936.1146 | 1937.1206 | 128.0704 | 199.1071 | 284.1598 | 355.1985 | 440.2528 | 525.3029 | n.d. | 738.4129 | 851.4953 | 922.5319 | 979.5613 | 1064.6081 | 1149.6611 | 788.4616 |
| Pept-B-XXIXb | 1936.1146 | 1937.1206 | 128.0704 | 199.1071 | 284.1598 | 355.1985 | 440.2528 | 511.2871 | n.d. | 724.3967 | 837.4860 | 922.5319 | 979.5613 | 1064.6081 | 1149.6611 | 788.4616 |
| Pept-B-XXX | 1950.1302 | 1951.1368 | 128.0704 | 199.1071 | 284.1598 | 355.1985 | 440.2528 | 525.3029 | n.d. | 738.4129 | 851.4953 | 936.5563 | 993.5684 | 1092.6402 | 1177.6921 | 774.4469 |
| Pept-B-XXXI | 1951.1143 | 1952.1302 | 128.0704 | 199.1071 | 284.1598 | 355.1985 | 440.2528 | 525.3029 | n.d. | 738.4129 | 851.4953 | 936.5563 | 993.5684 | 1078.6224 | 1163.6769 | 789.4509 |
| Pept-B-XXXIIa | 1950.1302 | 1951.1368 | 128.0704 | 199.1071 | 284.1598 | 355.1985 | 440.2528 | 525.3029 | n.d. | 738.4129 | 851.4953 | 936.5563 | 993.5684 | 1078.6224 | 1163.6769 | 788.4616 |
| Pept-B-XXXIIb | 1964.1459 | 1965.1522 | 128.0704 | 199.1071 | 284.1598 | 355.1985 | 440.2528 | 539.3186 | n.d. | 752.4301 | 865.5170 | 950.5699 | 1007.5874 | 1092.6402 | 1177.6921 | 788.4616 |
| Pept-B-XXXIIIa | 1936.1146 | 1937.1206 | 128.0704 | 199.1071 | 284.1598 | 355.1985 | 440.2528 | 525.3029 | n.d. | 738.4129 | 851.4953 | 936.5563 | 993.5684 | 1078.6224 | 1163.6769 | 774.4469 |
| Pept-B-XXXIIIb | 1936.1146 | 1937.1206 | 128.0704 | 199.1071 | 284.1598 | 355.1985 | 440.2528 | 511.2871 | n.d. | 724.3967 | 837.4860 | 922.5319 | 979.5613 | 1078.6224 | 1163.6769 | 774.4469 |
| Pept-B-XXXIIIc | 1950.1302 | 1951.1368 | 128.0704 | 199.1071 | 284.1598 | 355.1985 | 440.2528 | 525.3029 | n.d. | 738.4129 | 851.4953 | 936.5563 | 993.5684 | 1092.6402 | 1177.6921 | 774.4469 |
| Pept-B-XXXIIId | 1951.1143 | 1952.1302 | 128.0704 | 199.1071 | 284.1598 | 355.1985 | 454.2668 | 539.3186 | n.d. | 752.4301 | 865.5170 | 950.5699 | 1007.5874 | 1092.6402 | 1177.6921 | 775.4322 |
| Pept-B-XXXIVa | 1937.0986 | 1938.0984 | 128.0704 | 199.1071 | 284.1598 | 355.1985 | 440.2528 | 525.3029 | n.d. | 738.4129 | 851.4953 | 922.5319 | 979.5613 | 1064.6081 | 1149.6611 | 789.4509 |
| Pept-B-XXXIVb | 1951.1143 | 1952.1302 | 128.0704 | 199.1071 | 284.1598 | 355.1985 | 440.2528 | 525.3029 | n.d. | 738.4129 | 851.4953 | 936.5563 | 993.5684 | 1078.6224 | 1163.6769 | 789.4509 |
| Pept-B-XXXVa | 1936.1146 | 1937.1206 | 128.0704 | 199.1071 | 284.1598 | 355.1985 | 440.2528 | 525.3029 | n.d. | 738.4129 | 851.4953 | 936.5563 | 993.5684 | 1078.6224 | 1163.6769 | 774.4469 |
| Pept-B-XXXVb | 1936.1146 | 1937.1206 | 128.0704 | 199.1071 | 284.1598 | 355.1985 | 440.2528 | 511.2871 | n.d. | 724.3967 | 837.4860 | 922.5319 | 979.5613 | 1078.6224 | 1163.6769 | 774.4469 |
| Pept-B-XXXVI | 1964.1459 | 1965.1522 | 128.0704 | 199.1071 | 298.1756 | 369.2134 | 468.2668 | 553.3335 | n.d. | 766.4425 | 879.5344 | 964.5869 | 1021.6084 | 1106.7307 | 1191.7080 | 774.4469 |
| Pept-B-XXXVII | 1937.0986 | 1938.0984 | 128.0704 | 199.1071 | 284.1598 | 355.1985 | 440.2528 | 525.3029 | n.d. | 738.4129 | 851.4953 | 936.5563 | 993.5684 | 1078.6224 | 1163.6769 | 775.4322 |
| Pept-B-XXXVIII | 1950.1302 | 1951.1368 | 128.0704 | 199.1071 | 284.1598 | 355.1985 | 440.2528 | 525.3029 | n.d. | 738.4129 | 851.4953 | 936.5563 | 993.5684 | 1078.6224 | 1163.6769 | 788.4616 |
| Pept-B-XXXIX | 1964.1459 | 1965.1522 | 128.0704 | 199.1071 | 284.1598 | 355.1985 | 454.2668 | 539.3186 | n.d. | 752.4301 | 865.5170 | 950.5699 | 1007.5874 | 1092.6402 | 1177.6921 | 788.4616 |
| Pept-B-XL | 1950.1302 | 1951.1368 | 128.0704 | 199.1071 | 284.1598 | 355.1985 | 440.2528 | 511.2871 | n.d. | 724.3967 | 837.4860 | 922.5319 | 979.5613 | 1078.6224 | 1163.6769 | 788.4616 |
| Pept-B- XLIa | 1964.1459 | 1965.1522 | 128.0704 | 199.1071 | 284.1598 | 355.1985 | 440.2528 | 525.3029 | n.d. | 738.4129 | 851.4953 | 936.5563 | 993.5684 | 1092.6402 | 1177.6921 | 788.4616 |
| Pept-B- XLIb | 1964.1459 | 1965.1522 | 128.0704 | 199.1071 | 284.1598 | 355.1985 | 440.2528 | 539.3186 | n.d. | 752.4301 | 865.5170 | 950.5699 | 1007.5874 | 1092.6402 | 1177.6921 | 788.4616 |
| Pept-B- XLIIa | 1950.1302 | 1951.1368 | 128.0704 | 199.1071 | 284.1598 | 355.1985 | 440.2528 | 525.3029 | n.d. | 738.4129 | 851.4953 | 936.5563 | 993.5684 | 1092.6402 | 1177.6921 | 774.4469 |
| Pept-B- XLIIb | 1936.1146 | 1937.1206 | 128.0704 | 199.1071 | 284.1598 | 355.1985 | 440.2528 | 525.3029 | n.d. | 738.4129 | 851.4953 | 936.5563 | 993.5684 | 1078.6224 | 1163.6769 | 774.4469 |
| Pept-B- XLIII | 1964.1459 | 1965.1522 | 128.0704 | 199.1071 | 284.1598 | 355.1985 | 454.2668 | 553.3335 | n.d. | 766.4425 | 879.5344 | 964.5869 | 1021.6084 | 1106.7307 | 1191.7080 | 774.4469 |

n.d.: not detected

**Supplementary Table 2 ǀ** continued

| **Peptide** | **M** | **[M+H]^+^** | **b_1_** | **b_2_** | **b_3_** | **b_4_** | **b_5_** | **b_6_** | **b_7_** | **b_8_** | **b_9_** | **b_10_** | **b_11_** | **b_12_** | **b_13_** | **y_7_** |
| --- | --- | --- | --- | --- | --- | --- | --- | --- | --- | --- | --- | --- | --- | --- | --- | --- |
| Pept-B- XLIV | 1965.1299 | 1966.1444 | 128.0704 | 199.1071 | 284.1598 | 355.1985 | 454.2668 | 553.3337 | n.d. | 766.4421 | 879.5344 | 964.5869 | 1021.6047 | 1106.6547 | 1191.7080 | 775.4322 |
| Pept-B- XLVa | 1950.1302 | 1951.1368 | 128.0704 | 199.1071 | 284.1598 | 355.1985 | 440.2528 | 525.3029 | n.d. | 738.4129 | 851.4953 | 936.5563 | 993.5684 | 1078.6224 | 1163.6769 | 788.4616 |
| Pept-B- XLVb | 1978.1615 | 1979.1713 | 128.0704 | 199.1071 | 284.1598 | 369.2134 | 454.2668 | 553.3335 | n.d. | 766.4425 | 879.5344 | 964.5869 | 1021.6084 | 1106.6547 | 1191.7080 | 788.4616 |
| Pept-B-XLVIa | 1936.1146 | 1937.1206 | 128.0704 | 199.1071 | 284.1598 | 355.1985 | 440.2528 | 525.3029 | n.d. | 738.4129 | 851.4953 | 936.5563 | 993.5684 | 1078.6224 | 1163.6769 | 774.4469 |
| Pept-B-XLVIb | 1950.1302 | 1951.1368 | 128.0704 | 199.1071 | 284.1598 | 355.1985 | 440.2528 | 525.3029 | n.d. | 738.4129 | 851.4953 | 936.5563 | 993.5684 | 1092.6402 | 1177.6921 | 774.4469 |
| Pept-B-XLVII | 1964.1459 | 1965.1522 | 128.0704 | 199.1071 | 284.1598 | 355.1985 | 454.2668 | 539.3186 | n.d. | 752.4301 | 865.5170 | 950.5699 | 1007.5874 | 1092.6402 | 1177.6921 | 789.4509 |
| Pept-B- XLVIIIa | 1950.1302 | 1951.1368 | 128.0704 | 199.1071 | 284.1598 | 355.1985 | 440.2528 | 511.2871 | n.d. | 724.3967 | 837.4860 | 922.5319 | 979.5613 | 1078.6224 | 1163.6769 | 788.4616 |
| Pept-B- XLVIIIb | 1964.1459 | 1965.1522 | 128.0704 | 199.1071 | 284.1598 | 355.1985 | 440.2528 | 525.3029 | n.d. | 738.4129 | 851.4953 | 936.5563 | 993.5684 | 1092.6402 | 1177.6921 | 788.4616 |
| Pept-B-XLIX | 1978.1615 | 1979.1713 | 128.0704 | 199.1071 | 298.1756 | 369.2134 | 454.2668 | 553.3335 | n.d. | 766.4425 | 879.5344 | 964.5869 | 1021.6084 | 1106.6547 | 1191.7080 | 788.4616 |
| Pept-B-L | 1965.1299 | 1966.1444 | 128.0704 | 199.1071 | 284.1598 | 355.1985 | 454.2668 | 553.3335 | n.d. | 766.4425 | 879.5344 | 964.5869 | 1021.6084 | 1106.6547 | 1191.7080 | 775.4322 |
| Pept-B-LI | 1978.1615 | 1979.1713 | 128.0704 | 199.1071 | 284.1598 | 369.2134 | 468.2816 | 567.3482 | n.d. | 780.4582 | 893.5424 | 978.6031 | 1035.6162 | 1120.6686 | 1205.7234 | 774.4469 |
| Pept-B-LII | 1951.1143 | 1952.1302 | 128.0704 | 199.1071 | 284.1598 | 355.1985 | 440.2528 | 525.3029 | n.d. | 738.4129 | 851.4953 | 936.5563 | 993.5684 | 1078.6224 | 1163.6769 | 789.4509 |
| Pept-B-LIII | 1950.1302 | 1951.1368 | 128.0704 | 199.1071 | 284.1598 | 355.1985 | 440.2528 | 525.3029 | n.d. | 738.4129 | 851.4953 | 936.5563 | 993.5684 | 1092.6402 | 1177.6921 | 774.4469 |
| Pept-B-LIV | 1978.1615 | 1979.1713 | 128.0704 | 199.1071 | 284.1598 | 355.1985 | 454.2668 | 553.3335 | n.d. | 766.4425 | 879.5344 | 964.5869 | 1021.6084 | 1106.6547 | 1191.7080 | 788.4616 |
| Pept-B-LV | 1964.1459 | 1965.1522 | 128.0704 | 199.1071 | 284.1598 | 355.1985 | 440.2528 | 525.3029 | n.d. | 738.4129 | 851.4953 | 936.5563 | 993.5684 | 1092.6402 | 1177.6921 | 788.4616 |
| Pept-B-LVI | 1950.1302 | 1951.1368 | 128.0704 | 199.1071 | 284.1598 | 355.1985 | 440.2528 | 511.2871 | n.d. | 724.3967 | 837.4860 | 922.5319 | 979.5613 | 1078.6224 | 1163.6769 | 788.4616 |
| Pept-B-LVII | 1979.1456 | 1980.1604 | 128.0704 | 199.1071 | 284.1598 | 355.1985 | 454.2668 | 553.3335 | n.d. | 766.4425 | 879.5344 | 964.5869 | 1021.6084 | 1106.6547 | 1191.7080 | 789.4509 |
| Pept-B-LVIII | 1936.1146 | 1937.1206 | 128.0704 | 199.1071 | 284.1598 | 355.1985 | 440.2528 | 525.3029 | n.d. | 738.4129 | 851.4953 | 936.5563 | 993.5684 | 1078.6224 | 1163.6769 | 774.4469 |
| Pept-B-LIX | 1992.1772 | 1993.1845 | 128.0704 | 199.1071 | 298.1756 | 369.2134 | 468.2816 | 567.3482 | n.d. | 780.4582 | 893.5424 | 978.6031 | 1035.6162 | 1120.6686 | 1205.7234 | 788.4616 |
| Pept-B-LXa | 1964.1459 | 1965.1522 | 128.0704 | 199.1071 | 284.1598 | 355.1985 | 440.2528 | 525.3029 | n.d. | 738.4129 | 837.4860 | 922.5319 | 979.5613 | 1092.6402 | 1177.6921 | 788.4616 |
| Pept-B-LXb | 1964.1459 | 1965.1522 | 128.0704 | 199.1071 | 284.1598 | 355.1985 | 440.2528 | 525.3029 | n.d. | 738.4129 | 851.4953 | 936.5563 | 993.5684 | 1092.6402 | 1177.6921 | 788.4616 |
| Pept-B-LXI | 1964.1459 | 1965.1522 | 128.0704 | 199.1071 | 284.1598 | 355.1985 | 440.2528 | 525.3029 | n.d. | 738.4129 | 851.4953 | 936.5563 | 993.5684 | 1092.6402 | 1177.6921 | 788.4616 |

n.d.: not detected

**Supplementary Table 3 ǀ** Diagnostic fragment ions of peptaibols detected with the full scan MS measurement of brevicelsins (group C)

| **Peptide** | **M** | **[M+H]^+^** | **b_1_** | **b_2_** | **b_3_** | **b_4_** | **b_5_** | **b_6_** | **b_7_** | **b_8_** | **b_9_** | **b_10_** | **b_11_** | **b_12_** | **b_13_** | **y_7_** |
| --- | --- | --- | --- | --- | --- | --- | --- | --- | --- | --- | --- | --- | --- | --- | --- | --- |
| Brevicelsin I | 1851.0618 | 1852.0668 | 128.0704 | 199.1071 | 284.1598 | 355.1985 | 440.2528 | - | n.d. | 653.3604 | 766.4414 | 851.4953 | 908.5167 | 993.5684 | 1078.6224 | 774.4469 |
| Brevicelsin II | 1865.0775 | 1866.0851 | 128.0704 | 199.1071 | 298.1756 | 369..2134 | 454.2668 | - | n.d. | 667.4159 | 780.4582 | 865.5097 | 922.5319 | 1007.5874 | 1092.6402 | 774.4469 |
| Brevicelsin III | 1852.0458 | 1853.0613 | 128.0704 | 199.1071 | 284.1598 | 355.1985 | 440.2528 | - | n.d. | 653.3604 | 766.4425 | 851.4953 | 908.5167 | 993.5684 | 1078.6224 | 775.4322 |
| Brevicelsin IV | 1865.0775 | 1866.0851 | 128.0704 | 199.1071 | 284.1598 | 355.1985 | 440.2528 | - | n.d. | 653.3604 | 766.4425 | 851.4953 | 908.5167 | 993.5684 | 1078.6224 | 788.4616 |
| Brevicelsin V | 1879.0931 | 1880.1007 | 128.0704 | 199.1071 | 284.1598 | 355.1985 | 454.2668 | - | n.d. | 667.3751 | 780.4582 | 865.5170 | 922.5319 | 1007.5874 | 1092.6402 | 788.4616 |
| Brevicelsin VI | 1865.0775 | 1866.0851 | 128.0704 | 199.1071 | 284.1598 | 355.1985 | 454.2668 | - | n.d. | 667.3751 | 780.4582 | 865.5170 | 922.5319 | 1007.5874 | 1092.6402 | 774.4469 |
| Brevicelsin VII | 1866.0615 | 1867.0745 | 128.0704 | 199.1071 | 284.1598 | 355.1985 | 440.2528 | - | n.d. | 653.3604 | 766.4425 | 851.4953 | 908.5167 | 993.5684 | 1078.6224 | 789.4509 |
| Brevicelsin VIII | 1879.0931 | 1880.1007 | 128.0704 | 199.1071 | 284.1598 | 355.1985 | 454.2668 | - | n.d. | 667.3751 | 780.4582 | 865.5170 | 922.5319 | 1007.5874 | 1092.6402 | 788.4616 |

n.d.: not detected

**Supplementary Table 4 ǀ** Diagnostic fragment ions of acylium ion (y_7_) detected with MS^2^ measurements of peptaibol compounds from group A

| **Peptide** | **y_7_** | **y_7_ - H_2_O *** | **y_7_ - AA (19)** | **y_7_ - AA (19-18)** | **y_7_ - AA (19-17)** | **y_7_ - AA (19-16)** | **y_7_ - AA (19-15)** |
| --- | --- | --- | --- | --- | --- | --- | --- |
| Pept-A-Ia | 774.4469 | 756.4 | 623.3480 | 495.2911 | 367.2330 | 282.1803 | 197.1279 |
| Pept-A-Ib | 774.4469 | 756.4 | 623.3480 | 495.2911 | 367.2330 | 282.1803 | 197.1279 |
| Pept-A-IIa | 775.4322 | 757.4 | 624.3356 | 496.2768 | 367.2330 | 282.1803 | 197.1279 |
| Pept-A-IIb | 775.4322 | 757.4 | 624.3356 | 496.2768 | 367.2330 | 282.1803 | 197.1279 |
| Pept-A-IIIa | 788.4616 | 770.3 | 637.3637 | 509.3106 | 381.2485 | 282.1804 | 197.1280 |
| Pept-A-IIIb | 788.4616 | 770.3 | 637.3637 | 509.3106 | 381.2485 | 282.1804 | 197.1280 |
| Pept-A-IIIc | 788.4616 | 770.3 | 637.3637 | 509.3106 | 381.2485 | 282.1804 | 197.1280 |
| Pept-A-IVa | 774.4469 | 756.4 | 623.3480 | 495.2911 | 367.2330 | 282.1803 | 197.1279 |
| Pept-A-IVb | 774.4469 | 756.4 | 623.3480 | 495.2911 | 367.2330 | 282.1803 | 197.1279 |
| Pept-A-Va | 774.4469 | 756.4 | 623.3480 | 495.2911 | 367.2330 | 282.1803 | 197.1279 |
| Pept-A-Vb | 774.4469 | 756.4 | 623.3480 | 495.2911 | 367.2330 | 282.1803 | 197.1279 |
| Pept-A-VIa | 775.4322 | 757.4 | 624.3356 | 496.2768 | 367.2330 | 282.1803 | 197.1279 |
| Pept-A-VIb | 775.4322 | 757.4 | 624.3356 | 496.2768 | 367.2330 | 282.1803 | 197.1279 |
| Pept-A-VIIa | 774.4469 | 756.4 | 623.3480 | 495.2911 | 367.2330 | 282.1803 | 197.1279 |
| Pept-A-VIIb | 774.4469 | 756.4 | 623.3480 | 495.2911 | 367.2330 | 282.1803 | 197.1279 |
| Pept-A-VIIIa | 774.4469 | 756.4 | 623.3480 | 495.2911 | 367.2330 | 282.1803 | 197.1279 |
| Pept-A-VIIIb | 774.4469 | 756.4 | 623.3480 | 495.2911 | 367.2330 | 282.1803 | 197.1279 |
| Pept-A-IXa | 788.4616 | 770.3 | 637.3637 | 509.3106 | 381.2485 | 282.1804 | 197.1280 |
| Pept-A-IXb | 788.4616 | 770.3 | 637.3637 | 509.3106 | 381.2485 | 282.1804 | 197.1280 |
| Pept-A-Xa | 788.4616 | 770.3 | 637.3637 | 509.3106 | 381.2485 | 282.1804 | 197.1280 |
| Pept-A-Xb | 788.4616 | 770.3 | 637.3637 | 509.3106 | 381.2485 | 282.1804 | 197.1280 |
| Pept-A-XIa | 775.4322 | 757.4 | 624.3356 | 496.2768 | 367.2330 | 282.1803 | 197.1279 |
| Pept-A-XIb | 775.4322 | 757.4 | 624.3356 | 496.2768 | 367.2330 | 282.1803 | 197.1279 |
| Pept-A-XIc | 775.4322 | 757.4 | 624.3356 | 496.2768 | 367.2330 | 282.1803 | 197.1279 |
| Pept-A-XII | 775.4322 | 757.4 | 624.3356 | 496.2768 | 367.2330 | 282.1803 | 197.1279 |

* determined on Varian-MS system

**Supplementary Table 4 ǀ** continued

| **Peptide** | **y_7_** | **y_7_ - H_2_O *** | **y_7_ - AA (19)** | **y_7_ - AA (19-18)** | **y_7_ - AA (19-17)** | **y_7_ - AA (19-16)** | **y_7_ - AA (19-15)** |
| --- | --- | --- | --- | --- | --- | --- | --- |
| Pept-A-XIIIa | 789.4509 | 771.3 | 638.3518 | 510.2926 | 381.2485 | 282.1804 | 197.1280 |
| Pept-A-XIIIb | 789.4509 | 771.3 | 638.3518 | 510.2926 | 381.2485 | 282.1804 | 197.1280 |
| Pept-A-XIVa | 789.4509 | 771.3 | 638.3518 | 510.2926 | 381.2485 | 282.1804 | 197.1280 |
| Pept-A- XIVb | 788.4616 | 770.3 | 637.3637 | 509.3106 | 381.2485 | 282.1804 | 197.1280 |
| Pept-A-XVa | 788.4616 | 770.3 | 637.3637 | 509.3106 | 381.2485 | 282.1804 | 197.1280 |
| Pept-A-XVb | 788.4616 | 770.3 | 637.3637 | 509.3106 | 381.2485 | 282.1804 | 197.1280 |
| Pept-A-XVIa | 774.4469 | 756.4 | 623.3480 | 495.2911 | 367.2330 | 282.1803 | 197.1279 |
| Pept-A-XVIb | 774.4469 | 756.4 | 623.3480 | 495.2911 | 367.2330 | 282.1803 | 197.1279 |
| Pept-A-XVIIa | 788.4616 | 770.3 | 637.3637 | 509.3106 | 381.2485 | 282.1804 | 197.1280 |
| Pept-A-XVIIb | 788.4616 | 770.3 | 637.3637 | 509.3106 | 381.2485 | 282.1804 | 197.1280 |
| Pept-A-XVIII | 788.4616 | 770.3 | 637.3637 | 509.3106 | 381.2485 | 282.1804 | 197.1280 |
| Pept-A-XIXa | 775.4322 | 757.4 | 624.3356 | 496.2768 | 367.2330 | 282.1803 | 197.1279 |
| Pept-A-XIXb | 775.4322 | 757.4 | 624.3356 | 496.2768 | 367.2330 | 282.1803 | 197.1279 |
| Pept-A-XX | 774.4469 | 756.4 | 623.3480 | 495.2911 | 367.2330 | 282.1803 | 197.1279 |
| Pept-A-XXIa | 788.4616 | 770.3 | 637.3637 | 509.3106 | 381.2485 | 282.1804 | 197.1280 |
| Pept-A-XXIb | 788.4616 | 770.3 | 637.3637 | 509.3106 | 381.2485 | 282.1804 | 197.1280 |
| Pept-A-XXIIa | 774.4469 | 756.4 | 623.3480 | 495.2911 | 367.2330 | 282.1803 | 197.1279 |
| Pept-A-XXIIb | 774.4469 | 756.4 | 623.3480 | 495.2911 | 367.2330 | 282.1803 | 197.1279 |
| Pept-A-XXIII | 789.4509 | 771.3 | 638.3518 | 510.2926 | 381.2485 | 282.1804 | 197.1280 |
| Pept-A-XXIV | 788.4616 | 770.3 | 637.3637 | 509.3106 | 381.2485 | 282.1804 | 197.1280 |
| Pept-A-XXVa | 788.4616 | 770.3 | 637.3637 | 509.3106 | 381.2485 | 282.1804 | 197.1280 |
| Pept-A-XXVb | 788.4616 | 770.3 | 637.3637 | 509.3106 | 381.2485 | 282.1804 | 197.1280 |
| Pept-A-XXVIa | 788.4616 | 770.3 | 637.3637 | 509.3106 | 381.2485 | 282.1804 | 197.1280 |
| Pept-A-XXVIb | 788.4616 | 770.3 | 637.3637 | 509.3106 | 381.2485 | 282.1804 | 197.1280 |
| Pept-A-XXVIIa | 789.4509 | 771.3 | 638.3518 | 510.2926 | 381.2485 | 282.1804 | 197.1280 |
| Pept-A-XXVIIb | 788.4616 | 770.3 | 637.3637 | 509.3106 | 381.2485 | 282.1804 | 197.1280 |

* determined on Varian-MS system

**Supplementary Table 5 ǀ** Diagnostic fragment ions of acylium ion (y_7_) detected with MS^2^ measurements of peptaibol compounds from group B

| **Peptide** | **y_7_** | **y_7_ - H_2_O *** | **y_7_ - AA (19)** | **y_7_ - AA (19-18)** | **y_7_ - AA (19-17)** | **y_7_ - AA (19-16)** | **y_7_ - AA (19-15)** |
| --- | --- | --- | --- | --- | --- | --- | --- |
| Pept-B-I | 774.4469 | 756.4 | 623.3480 | 495.2911 | 367.2330 | 282.1803 | 197.1279 |
| Pept-B-II | 774.4469 | 756.4 | 623.3480 | 495.2911 | 367.2330 | 282.1803 | 197.1279 |
| Pept-B-III | 774.4469 | 756.4 | 623.3480 | 495.2911 | 367.2330 | 282.1803 | 197.1279 |
| Pept-B-IV | 788.4616 | 770.3 | 637.3637 | 509.3106 | 381.2485 | 282.1804 | 197.1280 |
| Pept-B-V | 774.4469 | 756.4 | 623.3480 | 495.2911 | 367.2330 | 282.1803 | 197.1279 |
| Pept-B-VI | 774.4469 | 756.4 | 623.3480 | 495.2911 | 367.2330 | 282.1803 | 197.1279 |
| Pept-B-VII | 788.4616 | 770.3 | 637.3637 | 509.3106 | 381.2485 | 282.1804 | 197.1280 |
| Pept-B-VIII | 788.4616 | 770.3 | 637.3637 | 509.3106 | 381.2485 | 282.1804 | 197.1280 |
| Pept-B-IXa | 774.4469 | 756.4 | 623.3480 | 495.2911 | 367.2330 | 282.1803 | 197.1279 |
| Pept-B-IXb | 774.4469 | 756.4 | 623.3480 | 495.2911 | 367.2330 | 282.1803 | 197.1279 |
| Pept-B-X | 788.4616 | 770.3 | 637.3637 | 509.3106 | 381.2485 | 282.1804 | 197.1280 |
| Pept-B-XI | 788.4616 | 770.3 | 637.3637 | 509.3106 | 381.2485 | 282.1804 | 197.1280 |
| Pept-B-XII | 774.4469 | 756.4 | 623.3480 | 495.2911 | 367.2330 | 282.1803 | 197.1279 |
| Pept-B-XIII | 788.4616 | 770.3 | 637.3637 | 509.3106 | 381.2485 | 282.1804 | 197.1280 |
| Pept-B-XIVa | 775.4322 | 757.4 | 624.3356 | 496.2768 | 367.2330 | 282.1803 | 197.1279 |
| Pept-B-XIVb | 775.4322 | 757.4 | 624.3356 | 496.2768 | 367.2330 | 282.1803 | 197.1279 |
| Pept-B-XVa | 788.4616 | 770.3 | 637.3637 | 509.3106 | 381.2485 | 282.1804 | 197.1280 |
| Pept-B-XVb | 788.4616 | 770.3 | 637.3637 | 509.3106 | 381.2485 | 282.1804 | 197.1280 |
| Pept-B-XVI | 774.4469 | 756.4 | 623.3480 | 495.2911 | 367.2330 | 282.1803 | 197.1279 |
| Pept-B-XVII | 788.4616 | 770.3 | 637.3637 | 509.3106 | 381.2485 | 282.1804 | 197.1280 |
| Pept-B-XVIII | 774.4469 | 756.4 | 623.3480 | 495.2911 | 367.2330 | 282.1803 | 197.1279 |
| Pept-B-XIX | 788.4616 | 770.3 | 637.3637 | 509.3106 | 381.2485 | 282.1804 | 197.1280 |
| Pept-B-XX | 774.4469 | 756.4 | 623.3480 | 495.2911 | 367.2330 | 282.1803 | 197.1279 |
| Pept-B-XXI | 789.4 | 771.3 | 638.3518 | 510.2926 | 381.2485 | 282.1804 | 197.1280 |
| Pept-B-XXII | 788.4616 | 770.3 | 637.3637 | 509.3106 | 381.2485 | 282.1804 | 197.1280 |
| Pept-B-XXIII | 774.4469 | 756.4 | 623.3480 | 495.2911 | 367.2330 | 282.1803 | 197.1279 |
| Pept-B-XXIV | 788.4616 | 770.3 | 637.3637 | 509.3106 | 381.2485 | 282.1804 | 197.1280 |
| Pept-B-XXV | 775.4322 | 757.4 | 624.3356 | 496.2768 | 367.2330 | 282.1803 | 197.1279 |
| Pept-B-XXVI | 774.4469 | 756.4 | 623.3480 | 495.2911 | 367.2330 | 282.1803 | 197.1279 |

* determined on Varian-MS system

**Supplementary Table 5 ǀ** continued

| **Peptide** | **y_7_** | **y_7_ - H_2_O *** | **y_7_ - AA (19)** | **y_7_ - AA (19-18)** | **y_7_ - AA (19-17)** | **y_7_ - AA (19-16)** | **y_7_ - AA (19-15)** |
| --- | --- | --- | --- | --- | --- | --- | --- |
| Pept-B-XXVII | 788.4616 | 770.3 | 637.3637 | 509.3106 | 381.2485 | 282.1804 | 197.1280 |
| Pept-B-XXVIII | 774.4469 | 756.4 | 623.3480 | 495.2911 | 367.2330 | 282.1803 | 197.1279 |
| Pept-B-XXIXa | 788.4616 | 770.3 | 637.3637 | 509.3106 | 381.2485 | 282.1804 | 197.1280 |
| Pept-B-XXIXb | 788.4616 | 770.3 | 637.3637 | 509.3106 | 381.2485 | 282.1804 | 197.1280 |
| Pept-B-XXX | 774.4469 | 756.4 | 623.3480 | 495.2911 | 367.2330 | 282.1803 | 197.1279 |
| Pept-B-XXXI | 789.4509 | 771.3 | 638.3518 | 510.2926 | 381.2485 | 282.1804 | 197.1280 |
| Pept-B-XXXIIa | 788.4616 | 770.3 | 637.3637 | 509.3106 | 381.2485 | 282.1804 | 197.1280 |
| Pept-B-XXXIIb | 788.4616 | 770.3 | 637.3637 | 509.3106 | 381.2485 | 282.1804 | 197.1280 |
| Pept-B-XXXIIIa | 774.4469 | 756.4 | 623.3480 | 495.2911 | 367.2330 | 282.1803 | 197.1279 |
| Pept-B-XXXIIIb | 774.4469 | 756.4 | 623.3480 | 495.2911 | 367.2330 | 282.1803 | 197.1279 |
| Pept-B-XXXIIIc | 774.4469 | 756.4 | 623.3480 | 495.2911 | 367.2330 | 282.1803 | 197.1279 |
| Pept-B-XXXIIId | 775.4322 | 757.4 | 624.3356 | 496.2768 | 367.2330 | 282.1803 | 197.1279 |
| Pept-B-XXXIVa | 789.4509 | 771.3 | 638.3518 | 510.2926 | 381.2485 | 282.1804 | 197.1280 |
| Pept-B-XXXIVb | 789.4509 | 771.3 | 638.3518 | 510.2926 | 381.2485 | 282.1804 | 197.1280 |
| Pept-B-XXXVa | 774.4469 | 756.4 | 623.3480 | 495.2911 | 367.2330 | 282.1803 | 197.1279 |
| Pept-B-XXXVb | 774.4469 | 756.4 | 623.3480 | 495.2911 | 367.2330 | 282.1803 | 197.1279 |
| Pept-B-XXXVI | 774.4469 | 756.4 | 623.3480 | 495.2911 | 367.2330 | 282.1803 | 197.1279 |
| Pept-B-XXXVII | 775.4322 | 757.4 | 624.3356 | 496.2768 | 367.2330 | 282.1803 | 197.1279 |
| Pept-B-XXXVIII | 788.4616 | 770.3 | 637.3637 | 509.3106 | 381.2485 | 282.1804 | 197.1280 |
| Pept-B-XXXIX | 788.4616 | 770.3 | 637.3637 | 509.3106 | 381.2485 | 282.1804 | 197.1280 |
| Pept-B-XL | 788.4616 | 770.3 | 637.3637 | 509.3106 | 381.2485 | 282.1804 | 197.1280 |
| Pept-B- XLIa | 788.4616 | 770.3 | 637.3637 | 509.3106 | 381.2485 | 282.1804 | 197.1280 |
| Pept-B- XLIb | 788.4616 | 770.3 | 637.3637 | 509.3106 | 381.2485 | 282.1804 | 197.1280 |
| Pept-B- XLIIa | 774.4469 | 756.4 | 623.3480 | 495.2911 | 367.2330 | 282.1803 | 197.1279 |
| Pept-B- XLIIb | 774.4469 | 756.4 | 623.3480 | 495.2911 | 367.2330 | 282.1803 | 197.1279 |
| Pept-B- XLIII | 774.4469 | 756.4 | 623.3480 | 495.2911 | 367.2330 | 282.1803 | 197.1279 |

* determined on Varian-MS system

**Supplementary Table 5 ǀ** continued

| **Peptide** | **y_7_** | **y_7_ - H_2_O *** | **y_7_ - AA (19)** | **y_7_ - AA (19-18)** | **y_7_ - AA (19-17)** | **y_7_ - AA (19-16)** | **y_7_ - AA (19-15)** |
| --- | --- | --- | --- | --- | --- | --- | --- |
| Pept-B- XLIV | 775.4322 | 757.4 | 624.3356 | 496.2768 | 367.2330 | 282.1803 | 197.1279 |
| Pept-B- XLVa | 788.4616 | 770.3 | 637.3637 | 509.3106 | 381.2485 | 282.1804 | 197.1280 |
| Pept-B- XLVb | 788.4616 | 770.3 | 637.3637 | 509.3106 | 381.2485 | 282.1804 | 197.1280 |
| Pept-B-XLVIa | 774.4469 | 756.4 | 623.3480 | 495.2911 | 367.2330 | 282.1803 | 197.1279 |
| Pept-B-XLVIb | 774.4469 | 756.4 | 623.3480 | 495.2911 | 367.2330 | 282.1803 | 197.1279 |
| Pept-B-XLVII | 789.4509 | 771.3 | 638.3518 | 510.2926 | 381.2485 | 282.1804 | 197.1280 |
| Pept-B- XLVIIIa | 788.4616 | 770.3 | 637.3637 | 509.3106 | 381.2485 | 282.1804 | 197.1280 |
| Pept-B- XLVIIIb | 788.4616 | 770.3 | 637.3637 | 509.3106 | 381.2485 | 282.1804 | 197.1280 |
| Pept-B-XLIX | 788.4616 | 770.3 | 637.3637 | 509.3106 | 381.2485 | 282.1804 | 197.1280 |
| Pept-B-L | 775.4322 | 757.4 | 624.3356 | 496.2768 | 367.2330 | 282.1803 | 197.1279 |
| Pept-B-LI | 774.4469 | 756.4 | 623.3480 | 495.2911 | 367.2330 | 282.1803 | 197.1279 |
| Pept-B-LII | 789.4509 | 771.3 | 638.3518 | 510.2926 | 381.2485 | 282.1804 | 197.1280 |
| Pept-B-LIII | 774.4469 | 756.4 | 623.3480 | 495.2911 | 367.2330 | 282.1803 | 197.1279 |
| Pept-B-LIV | 788.4616 | 770.3 | 637.3637 | 509.3106 | 381.2485 | 282.1804 | 197.1280 |
| Pept-B-LV | 788.4616 | 770.3 | 637.3637 | 509.3106 | 381.2485 | 282.1804 | 197.1280 |
| Pept-B-LVI | 788.4616 | 770.3 | 637.3637 | 509.3106 | 381.2485 | 282.1804 | 197.1280 |
| Pept-B-LVII | 789.4509 | 771.3 | 638.3518 | 510.2926 | 381.2485 | 282.1804 | 197.1280 |
| Pept-B-LVIII | 774.4469 | 756.4 | 623.3480 | 495.2911 | 367.2330 | 282.1803 | 197.1279 |
| Pept-B-LIX | 788.4616 | 770.3 | 637.3637 | 509.3106 | 381.2485 | 282.1804 | 197.1280 |
| Pept-B-LXa | 788.4616 | 770.3 | 637.3637 | 509.3106 | 381.2485 | 282.1804 | 197.1280 |
| Pept-B-LXb | 788.4616 | 770.3 | 637.3637 | 509.3106 | 381.2485 | 282.1804 | 197.1280 |
| Pept-B-LXI | 788.4616 | 770.3 | 637.3637 | 509.3106 | 381.2485 | 282.1804 | 197.1280 |

* determined on Varian-MS system

**Supplementary Table 6 ǀ** Diagnostic fragment ions of acylium ion (y_7_) detected with MS^2^ measurements of brevicelsins (group C)

| **Peptide** | **y_7_** | **y_7_ - H_2_O *** | **y_7_ - AA (19)** | **y_7_ - AA (19-18)** | **y_7_ - AA (19-17)** | **y_7_ - AA (19-16)** | **y_7_ - AA (19-15)** |
| --- | --- | --- | --- | --- | --- | --- | --- |
| Brevicelsin I | 774.4469 | 756.4 | 623.3480 | 495.2911 | 367.2330 | 282.1803 | 197.1279 |
| Brevicelsin II | 774.4469 | 756.4 | 623.3480 | 495.2911 | 367.2330 | 282.1803 | 197.1279 |
| Brevicelsin III | 775.4322 | 757.4 | 624.3356 | 496.2768 | 367.2330 | 282.1803 | 197.1279 |
| Brevicelsin IV | 788.4616 | 770.3 | 637.3637 | 509.3106 | 381.2485 | 282.1804 | 197.1280 |
| Brevicelsin V | 788.4616 | 770.3 | 637.3637 | 509.3106 | 381.2485 | 282.1804 | 197.1280 |
| Brevicelsin VI | 774.4469 | 756.4 | 623.3480 | 495.2911 | 367.2330 | 282.1803 | 197.1279 |
| Brevicelsin VII | 789.4509 | 771.3 | 638.3518 | 510.2926 | 381.2485 | 282.1804 | 197.1280 |
| Brevicelsin VIII | 788.4616 | 770.3 | 637.3637 | 509.3106 | 381.2485 | 282.1804 | 197.1280 |

* determined on Varian-MS system

**Supplementary Table 7 ǀ** Relative amounts of the identified peptaibol compounds in the peptaibol profiles of *T. aethipicum, T. pinnatum, T. capillare, T. pseudokoningii, T. citrinoviride, T. longibrachiatum, T. orientale* and *T. novae-zelandiae.* The colours represent the extent of the production (percentage) of each strain from the lowest (yellow) to the highest (red).

| **Peptaibol** | ***T. aethiopi-cum*** | ***T. pinnatum*** | ***T. ghanense*** | ***T. capillare*** | ***T. pseudo-koningii*** | ***T. citrino-viride*** | ***T. longibrachi-atum*** | ***T. longibrachi-atum*** | ***T. longibrachi-atum*** | ***T. longibrachi-atum*** | ***T. orientale*** | ***T. novae-zelandiae*** |
| --- | --- | --- | --- | --- | --- | --- | --- | --- | --- | --- | --- | --- |
|  | **22602** | **22603** | **22604** | **22605** | **22613** | **22618** | **1773** | **1775** | **1776** | **12546** | **12556** | **22612** |
| **Pept-A-Ia** |  |  |  |  |  |  |  |  |  | **2.01%** | **5.19%** |  |
| **Pept-A-Ib** |  |  |  |  |  |  |  |  |  |  |  | **3.19%** |
| **Pept-A-IIa** |  |  |  |  |  |  | **2.20%** | **1.58%** | **1.15%** |  |  |  |
| **Pept-A-IIb** |  |  |  |  |  |  |  |  |  | **0.40%** |  |  |
| **Pept-A-IIIa** |  |  |  |  | **1.24%** |  |  |  |  |  |  |  |
| **Pept-A-IIIb** |  |  |  |  |  |  |  |  |  |  | **2.06%** |  |
| **Pept-A-IIIc** |  |  |  |  |  |  |  |  |  |  |  | **1.99%** |
| **Pept-A-IVa** | **27.09%** | **31.98%** |  | **29.78%** | **16.71%** | **8.97%** | **25.40%** | **42.77%** | **14.52%** | **43.21%** | **47.08%** | **26.43%** |
| **Pept-A-IVb** |  |  | **19.67%** |  |  |  |  |  |  |  |  |  |
| **Pept-A-Va** |  |  |  |  | **5.30%** |  |  |  |  |  |  |  |
| **Pept-A-Vb** |  |  |  |  |  |  |  |  |  |  |  | **3.65%** |
| **Pept-A-VIa** |  |  |  | **9.94%** | **2.14%** | **0.78%** | **16.36%** | **14.57%** | **23.34%** | **5.81%** | **1.90%** |  |
| **Pept-A-VIb** | **6.89%** | **10.39%** | **28.06%** |  |  |  |  |  |  |  |  |  |
| **Pept-A-VIIa** |  |  |  |  |  | **0.73%** |  |  |  |  |  |  |
| **Pept-A-VIIb** | **3.77%** |  |  |  |  |  |  |  |  |  |  |  |
| **Pept-A-VIIIa** |  | **10.50%** |  |  |  |  |  |  |  |  |  |  |
| **Pept-A-VIIIb** |  |  |  |  |  |  |  | **3.04%** |  |  |  |  |
| **Pept-A-IXa** | **3.05%** |  |  | **21.94%** | **30.27%** | **24.51%** | **12.00%** | **1.98%** | **7.79%** | **16.72%** | **34.48%** |  |
| **Pept-A-IXb** |  | **1.60%** | **22.55%** |  |  |  |  |  |  |  |  | **47.01%** |
| **Pept-A-Xa** |  |  |  |  | **8.69%** |  |  |  |  |  |  |  |
| **Pept-A-XIa** | **2.05%** |  |  |  |  |  |  | **1.29%** | **0.35%** |  |  |  |
| **Pept-A-XIb** |  |  |  |  | **1.44%** |  | **0.69%** |  |  |  |  |  |
| **Pept-A-XIc** |  | **3.76%** |  |  |  |  |  |  |  |  |  |  |
| **Pept-A-XII** |  |  |  |  |  |  |  |  |  |  |  | **0.54%** |
| **Pept-A-XIIIa** |  |  |  | **6.78%** |  |  | **5.01%** | **1.42%** | **7.70%** | **0.79%** |  |  |
| **Pept-A-XIIIb** |  |  | **17.97%** |  |  |  |  |  |  |  |  |  |
| **Pept-A-XIVa** | **0.48%** | **0.36%** |  |  |  |  |  |  |  |  |  | **3.48%** |
| **Pept-A-XIVb** |  |  |  |  | **2.82%** | **1.99%** |  |  |  |  |  |  |
| **Pept-A-XVa** | **0.29%** |  |  | **2.88%** | **2.68%** | **2.55%** | **1.86%** | **0.31%** | **1.96%** | **1.68%** | **6.67%** |  |
| **Pept-A-XVb** |  | **0.56%** |  |  |  |  |  |  |  |  |  | **4.83%** |
| **Pept-A-XVIa** | **36.48%** | **24.23%** | **1.61%** | **12.08%** | **8.19%** | **14.79%** | **15.26%** | **23.21%** | **11.18%** | **19.80%** | **1.45%** |  |
| **Pept-A-XVIb** |  |  |  |  |  |  |  |  |  |  |  | **3.00%** |
| **Pept-A-XVIIa** |  | **0.16%** |  |  | **5.10%** |  | **0.15%** |  |  |  |  |  |
| **Pept-A-XVIIb** |  |  |  |  |  | **1.24%** |  |  | **0.27%** |  |  |  |
| **Pept-A-XVIII** | **0.32%** |  |  |  |  |  |  |  |  |  |  |  |
| **Pept-A-XIXa** | **6.75%** | **5.61%** | **1.97%** | **3.45%** | **0.61%** | **0.61%** | **8.44%** | **6.72%** | **13.19%** | **1.91%** |  |  |
| **Pept-A-XX** | **4.85%** | **6.31%** |  |  |  |  |  | **1.47%** | **0.40%** |  |  |  |
| **Pept-A-XXIa** | **3.51%** | **1.39%** | **2.54%** | **8.80%** | **14.80%** | **35.47%** | **7.28%** | **0.93%** | **6.06%** | **6.80%** | **1.16%** |  |
| **Pept-A-XXIb** |  |  |  |  |  |  |  |  |  |  |  | **5.87%** |
| **Pept-A-XXIIa** | **3.01%** | **2.23%** |  |  |  |  |  |  |  |  |  |  |
| **Pept-A-XXIIb** |  |  |  |  |  | **0.50%** |  |  |  |  |  |  |
| **Pept-A-XXIII** | **0.31%** | **0.26%** | **1.49%** | **2.54%** |  |  | **2.84%** | **0.49%** | **4.23%** | **0.23%** |  |  |
| **Pept-A-XXIV** | **0.75%** |  |  |  |  |  |  |  |  |  |  |  |
| **Pept-A-XXVa** |  | **0.64%** |  | **1.80%** |  |  | **1.76%** |  | **7.51%** | **0.65%** |  |  |
| **Pept-A-XXVb** |  |  |  |  |  | **4.24%** |  | **0.19%** |  |  |  |  |
| **Pept-A-XXVIa** | **0.40%** |  |  |  |  |  |  |  |  |  |  |  |
| **Pept-A-XXVIb** |  |  |  |  |  | **2.47%** |  |  |  |  |  |  |
| **Pept-A-XXVIIa** |  |  |  |  |  |  |  |  | **0.34%** |  |  |  |
| **Pept-A-XXVIIb** |  |  |  |  |  | **1.14%** |  |  |  |  |  |  |

**Supplementary Table 8 ǀ** Relative amounts of the identified peptaibol compounds in the peptaibol profiles of *T. reesei, T. saturnisporum, T. andinense, T. effusum, T. parareesei, T. flagellatum, T. sinense* and *T. konilangbra.* The colours represent the extent of the production (percentage) of each strain from the lowest (yellow) to the highest (red).

| **Peptaibol** | ***T. reesei*** | ***T. reesei*** | ***T. reesei Δ*** | ***T. saturnisporum*** | ***T. andinense*** | ***T. effusum*** | ***T. parareesei*** | ***T. flagellatum*** | ***T. sinense*** | ***T. konilangbra*** |
| --- | --- | --- | --- | --- | --- | --- | --- | --- | --- | --- |
|  | **22614** | **22616** | **22617** | **22606** | **22610** | **22611** | **22615** | **22608** | **22609** | **22607** |
| **Pept-B-I** | **0.62%** |  |  |  |  |  |  |  |  |  |
| **Pept-B-II** | **0.55%** |  |  |  |  |  |  |  |  |  |
| **Pept-B-III** | **0.37%** |  |  |  |  |  |  |  |  |  |
| **Pept-B-IV** | **0.78%** |  |  |  |  |  |  |  |  |  |
| **Pept-B-V** | **0.58%** |  |  |  |  |  |  |  |  |  |
| **Pept-B-VI** | **0.98%** |  |  |  |  |  |  |  |  |  |
| **Pept-B-VII** | **0.88%** |  |  |  |  |  |  |  |  |  |
| **Pept-B-VIII** |  | **1.47%** | **2.33%** |  |  |  |  |  |  |  |
| **Pept-B-IXa** | **3.61%** | **4.23%** | **3.59%** |  |  |  |  |  |  |  |
| **Pept-B-IXb** |  |  |  |  |  |  | **0.92%** |  |  |  |
| **Pept-B-X** | **0.44%** |  |  |  |  |  |  |  |  |  |
| **Pept-B-XI** | **0.65%** |  |  |  |  |  |  |  |  |  |
| **Pept-B-XII** | **10.85%** | **11.96%** | **16.31%** | **6.88%** | **4.23%** | **10.83%** | **5.00%** | **2.69%** |  |  |
| **Pept-B-XIII** | **0.54%** |  |  |  |  |  |  |  |  |  |
| **Pept-B-XIVa** |  |  |  | **0.44%** | **1.85%** | **0.87%** | **1.08%** | **0.41%** |  |  |
| **Pept-B-XIVb** | **0.52%** | **0.64%** | **1.09%** |  |  |  |  |  |  |  |
| **Pept-B-XVa** | **4.67%** | **5.80%** | **6.38%** |  |  |  |  |  |  |  |
| **Pept-B-XVb** |  |  |  |  |  |  | **0.95%** |  |  |  |
| **Pept-B-XVI** | **0.19%** |  |  |  |  |  |  |  |  |  |
| **Pept-B-XVII** | **13.80%** | **17.22%** | **21.61%** | **27.43%** | **9.41%** | **19.57%** | **7.10%** | **1.20%** |  |  |
| **Pept-B-XVIII** | **0.65%** |  |  |  |  |  |  |  |  |  |
| **Pept-B-XIX** | **1.02%** |  |  |  |  |  |  | **0.29%** |  |  |
| **Pept-B-XX** | **15.04%** | **15.88%** | **17.04%** | **9.33%** | **13.51%** | **16.03%** | **11.55%** | **9.54%** | **7.41%** | **4.75%** |
| **Pept-B-XXI** |  |  |  | **0.53%** | **1.25%** | **1.21%** | **0.69%** |  |  |  |
| **Pept-B-XXII** | **1.01%** |  |  |  |  |  |  |  |  |  |
| **Pept-B-XXIII** |  |  |  |  | **4.01%** |  | **3.26%** |  |  |  |
| **Pept-B-XXIV** | **0.50%** |  |  |  |  |  |  |  |  |  |
| **Pept-B-XXV** | **1.08%** | **1.11%** | **1.48%** | **0.60%** | **4.15%** | **0.67%** | **4.12%** | **1.76%** |  |  |
| **Pept-B-XXVI** |  |  |  |  |  |  |  | **1.38%** | **3.59%** |  |
| **Pept-B-XXVII** | **22.12%** | **24.75%** | **19.40%** | **40.50%** | **21.07%** | **36.54%** | **17.23%** | **5.60%** | **1.96%** | **3.70%** |
| **Pept-B-XXVIII** |  |  |  |  |  |  |  | **9.50%** | **14.33%** | **7.97%** |
| **Pept-B-XXIXa** |  |  |  |  | **8.69%** |  |  |  |  |  |
| **Pept-B-XXIXb** |  |  |  |  |  |  | **7.10%** |  |  |  |
| **Pept-B-XXX** | **1.49%** | **2.40%** | **0.70%** |  |  |  |  |  |  |  |
| **Pept-B-XXXI** |  |  |  |  | **2.61%** |  |  | **1.63%** |  |  |
| **Pept-B-XXXIIa** | **2.24%** | **2.08%** | **1.80%** |  |  |  |  |  |  |  |
| **Pept-B-XXXIIb** |  |  |  |  |  |  |  | **1.12%** | **2.10%** |  |
| **Pept-B-XXXIIIa** |  |  |  |  | **7.04%** |  | **8.55%** |  |  |  |
| **Pept-B-XXXIIIb** | **0.90%** |  |  |  |  |  |  |  |  |  |
| **Pept-B-XXXIIIc** |  | **0.56%** | **0.81%** |  |  |  |  |  |  |  |
| **Pept-B-XXXIIId** |  |  |  |  |  |  |  | **3.18%** |  |  |
| **Pept-B-XXXIVa** |  |  |  |  | **1.66%** |  |  |  |  |  |
| **Pept-B-XXXIVb** |  |  |  |  |  | **1.05%** | **1.89%** |  |  |  |
| **Pept-A-IVa** |  |  |  | **3.49%** |  |  |  |  |  |  |
| **Pept-A-IVb** |  |  |  |  |  |  |  |  |  | **22.06%** |
| **Pept-B-XXXVa** |  | **0.82%** | **1.47%** |  |  | **2.63%** |  |  |  |  |
| **Pept-B-XXXVb** | **0.93%** |  |  |  | **2.78%** |  |  |  |  |  |

**Supplementary Table 8 ǀ** continued

| **Peptaibol** | ***T. reesei*** | ***T. reesei*** | ***T. reesei Δ*** | ***T. saturnisporum*** | ***T. andinense*** | ***T. effusum*** | ***T. parareesei*** | ***T. flagellatum*** | ***T. sinense*** | ***T. konilangbra*** |
| --- | --- | --- | --- | --- | --- | --- | --- | --- | --- | --- |
|  | **22614** | **22616** | **22617** | **22606** | **22610** | **22611** | **22615** | **22608** | **22609** | **22607** |
| **Pept-B-XXXVI** |  |  |  |  |  |  |  | **2.08%** | **9.12%** |  |
| **Pept-B-XXXVII** |  |  |  |  | **1.17%** |  | **1.86%** |  |  |  |
| **Pept-B-XXXVIII** |  | **1.35%** | **0.84%** |  |  |  |  |  |  |  |
| **Pept-B-XXXIX** |  |  |  |  |  |  |  | **0.70%** |  |  |
| **Pept-B-XL** | **1.90%** | **1.39%** | **0.55%** | **3.39%** |  |  |  |  |  |  |
| **Pept-B-XLIa** | **3.09%** | **2.63%** | **0.71%** |  | **3.53%** |  | **3.75%** |  |  |  |
| **Pept-B-XLIb** |  |  |  |  |  |  |  | **9.36%** | **5.44%** | **7.47%** |
| **Pept-B-XLIIa** | **0.78%** |  |  |  |  |  | **0.44%** |  |  |  |
| **Pept-B-XLIIb** |  | **0.65%** | **0.82%** |  |  |  |  |  |  |  |
| **Pept-B-XLIII** |  |  |  |  |  |  |  | **6.53%** | **14.94%** |  |
| **Pept-A-VIa** |  |  |  | **1.26%** |  |  |  |  |  |  |
| **Pept-B-XLIV** |  |  |  |  |  |  |  |  |  | **10.56%** |
| **Pept-B-XLVa** | **2.11%** | **2.83%** | **0.59%** | **0.71%** | **8.42%** | **9.78%** | **11.62%** |  |  |  |
| **Pept-B-XLVb** |  |  |  |  |  |  |  |  |  | **6.35%** |
| **Pept-B-XLVIa** | **0.16%** | **0.34%** | **0.33%** |  |  |  |  |  |  |  |
| **Pept-B-XLVIb** |  |  |  |  |  |  | **0.25%** |  |  |  |
| **Pept-B-XLVII** |  |  |  |  |  |  |  | **1.92%** |  |  |
| **Pept-A-Xa** |  |  |  | **0.65%** |  |  |  |  |  |  |
| **Pept-A-Xb** | **0.92%** |  |  |  |  |  |  |  |  |  |
| **Pept-B-XLVIIIa** |  |  |  |  | **3.17%** |  | **1.24%** |  |  |  |
| **Pept-B-XLVIIIb** |  | **1.05%** | **1.78%** |  |  |  |  |  |  |  |
| **Pept-B-XLIX** |  |  |  |  |  |  |  | **2.70%** | **4.60%** |  |
| **Pept-B-L** |  |  |  |  |  |  |  | **1.43%** |  |  |
| **Pept-B-LI** |  |  |  |  |  |  |  | **1.86%** | **11.70%** | **4.69%** |
| **Pept-B-LII** |  |  |  |  | **1.45%** | **0.83%** | **1.28%** |  |  |  |
| **Pept-B-LIII** | **0.26%** |  |  |  |  |  | **1.03%** |  |  |  |
| **Pept-B-LIV** |  |  |  |  |  |  |  | **10.23%** | **7.65%** | **13.09%** |
| **Pept-B-LV** | **1.42%** | **0.85%** | **0.40%** |  |  |  | **0.90%** |  |  |  |
| **Pept-A-XVIa** |  |  |  | **3.15%** |  |  |  |  |  |  |
| **Pept-A-XVIb** | **0.44%** |  |  |  |  |  |  |  |  | **10.85%** |
| **Pept-B-LVI** | **0.49%** |  |  |  |  |  | **0.84%** |  |  |  |
| **Pept-B-LVII** |  |  |  |  |  |  |  | **2.28%** |  |  |
| **Pept-B-LVIII** | **0.31%** |  |  |  |  |  | **0.47%** |  |  |  |
| **Pept-B-LIX** |  |  |  |  |  |  |  | **4.13%** | **6.85%** | **6.20%** |
| **Pept-B-LXa** | **0.51%** |  |  |  |  |  |  |  |  |  |
| **Pept-B-LXb** |  |  |  |  |  |  | **2.41%** |  |  |  |
| **Pept-A-XIXa** | **0.12%** |  |  | **0.89%** |  |  |  |  |  |  |
| **Pept-A-XIXb** |  |  |  |  |  |  |  |  |  | **2.30%** |
| **Pept-B-LXI** | **0.47%** |  |  | **0.76%** |  |  | **0.99%** |  |  |  |
| **Brevicelsin I** |  |  |  |  |  |  | **0.81%** | **4.48%** | **2.09%** |  |
| **Brevicelsin II** |  |  |  |  |  |  |  |  | **1.81%** |  |
| **Brevicelsin III** |  |  |  |  |  |  |  | **0.95%** |  |  |
| **Brevicelsin IV** |  |  |  |  |  |  | **2.68%** | **3.20%** | **0.87%** |  |
| **Brevicelsin V** |  |  |  |  |  |  |  |  | **0.42%** |  |
| **Brevicelsin VI** |  |  |  |  |  |  |  | **4.99%** | **3.31%** |  |
| **Brevicelsin VII** |  |  |  |  |  |  |  | **0.42%** |  |  |
| **Brevicelsin VIII** |  |  |  |  |  |  |  | **4.41%** | **1.82%** |  |
