# Supplementary material for: Structural Diversity and Bioactivities of Peptaibol Compounds From the Longibrachiatum Clade of the Filamentous Fungal Genus Trichoderma
Source: Front Microbiol. 2019 Jun 26;10:1434. doi: 10.3389/fmicb.2019.01434 (PMC6606783; doi:10.3389/fmicb.2019.01434)
Supplement: Supplementary file 2 [file Data_Sheet_2.docx]

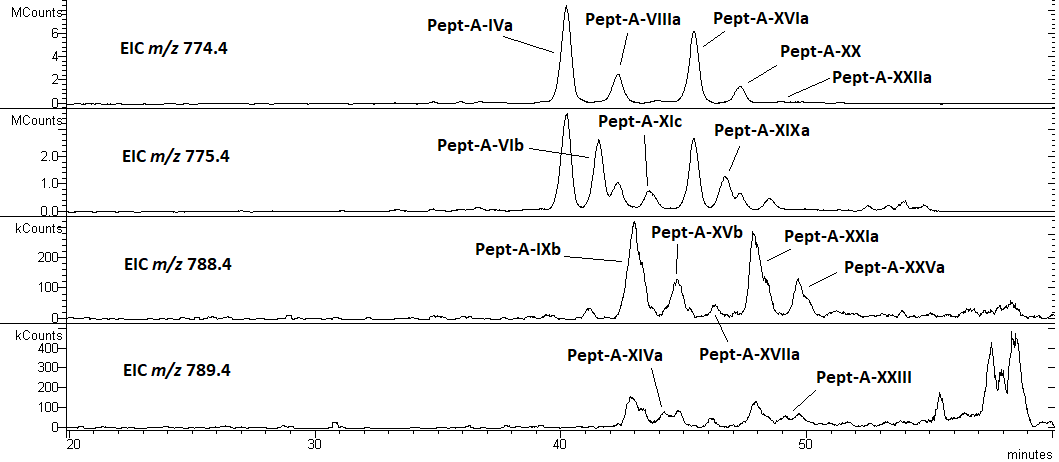


**Supplementary Figure 1 ǀ** Extracted ion chromatograms (EIC) resulting from full scan measurements of crude extracts from *T. pinnatum* SZMC 22603. The coeluting components within some peaks were identified based on MS^2^ experiments.


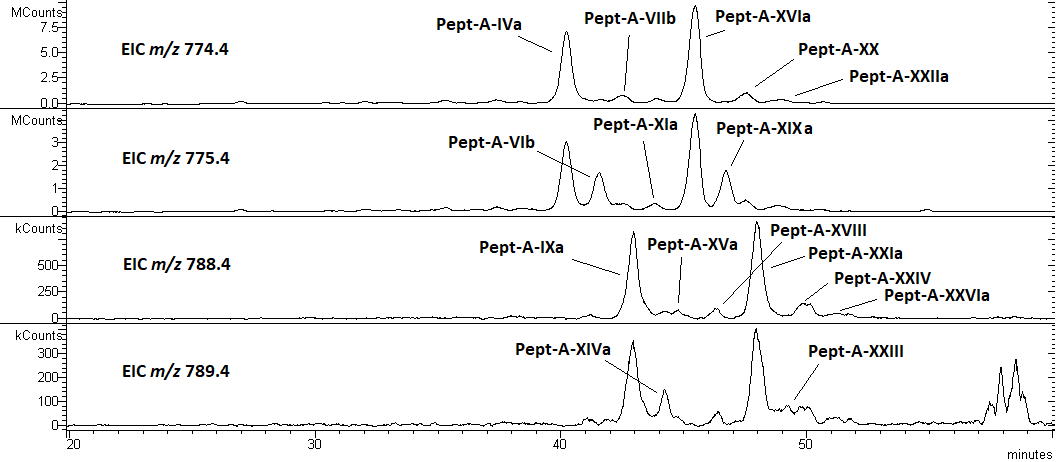


**Supplementary Figure 2 ǀ** Extracted ion chromatograms (EIC) resulting from full scan measurements of crude extracts from *T. aethiopicum* SZMC 22602. The coeluting components within some peaks were identified based on MS^2^ experiments.

**
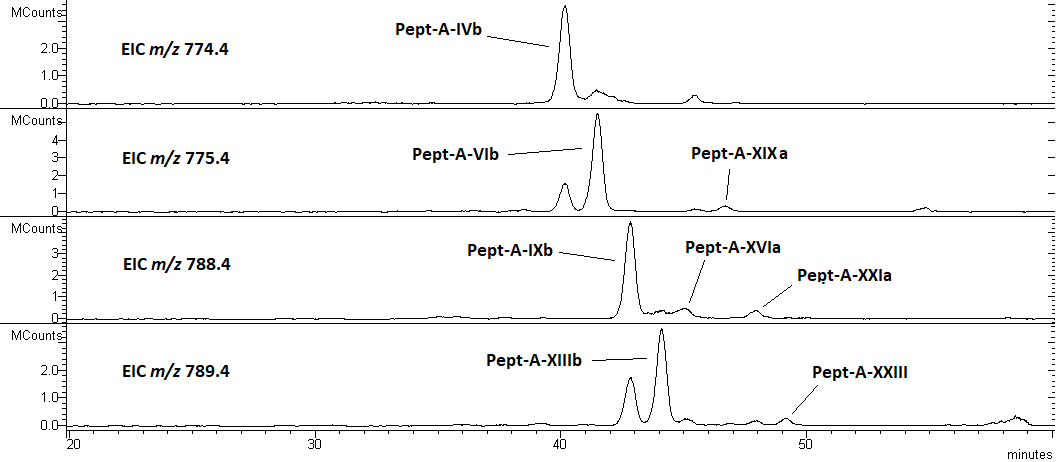
**

**Supplementary Figure 3 ǀ** Extracted ion chromatograms (EIC) resulting from full scan measurements of crude extracts from *T. ghanense* SZMC 22604. The coeluting components within some peaks were identified based on MS^2^ experiments.

**
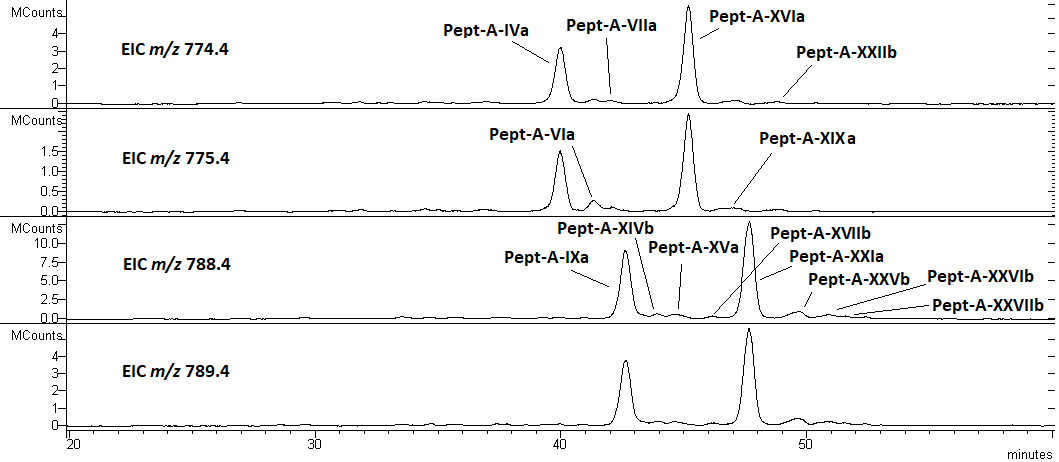
**

**Supplementary Figure 4 ǀ** Extracted ion chromatograms (EIC) resulting from full scan measurements of crude extracts from *T. citrinoviride* SZMC 22618. The coeluting components within some peaks were identified based on MS^2^ experiments.

**
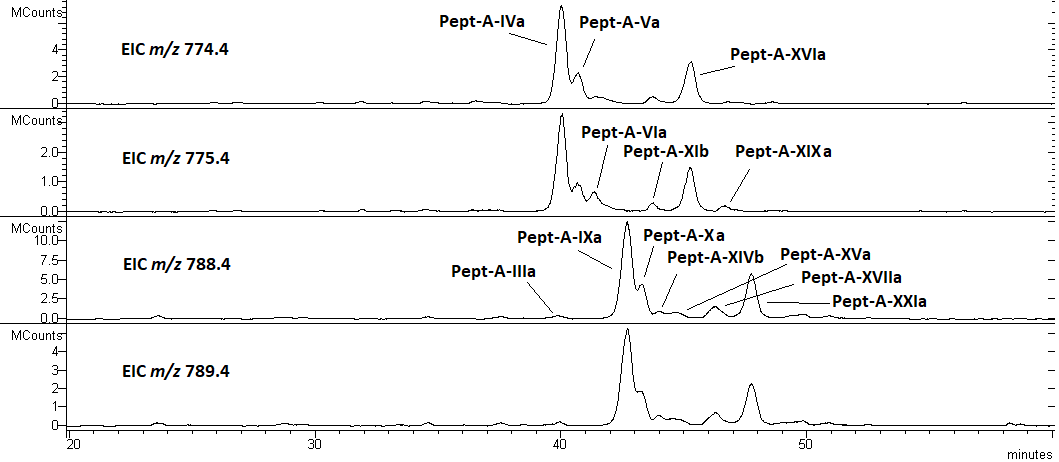
**

**Supplementary Figure 5 ǀ** Extracted ion chromatograms (EIC) resulting from full scan measurements of crude extracts from *T. pseudokoningii* SZMC 22613. The coeluting components within some peaks were identified based on MS^2^ experiments.

**
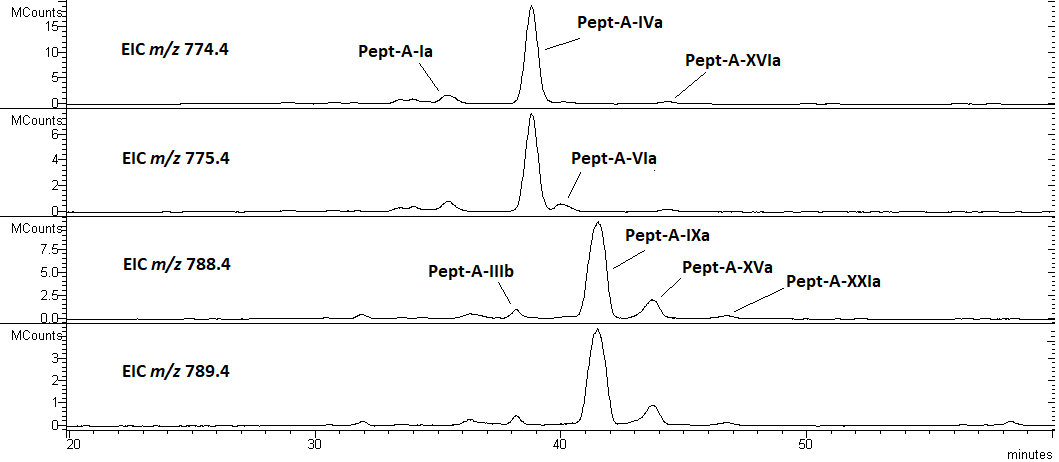
**

**Supplementary Figure 6 ǀ** Extracted ion chromatograms (EIC) resulting from full scan measurements of crude extracts from *T. orientale* SZMC 12556. The coeluting components within some peaks were identified based on MS^2^ experiments.

**
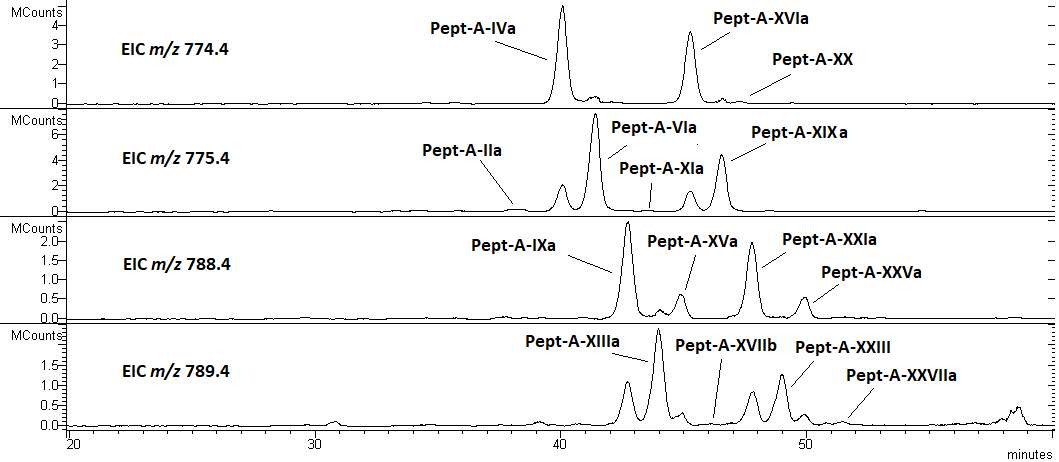
**

**Supplementary Figure 7 ǀ** Extracted ion chromatograms (EIC) resulting from full scan measurements of crude extracts from *T. longibrachiatum* SZMC 1776. The coeluting components within some peaks were identified based on MS^2^ experiments.

**
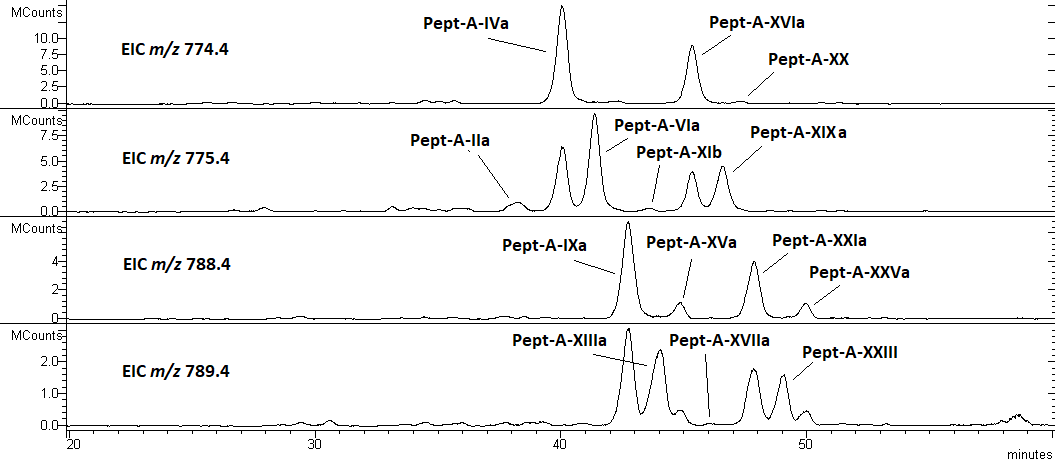
**

**Supplementary Figure 8 ǀ** Extracted ion chromatograms (EIC) resulting from full scan measurements of crude extracts from *T. longibrachiatum* SZMC 1773. The coeluting components within some peaks were identified based on MS^2^ experiments.

**
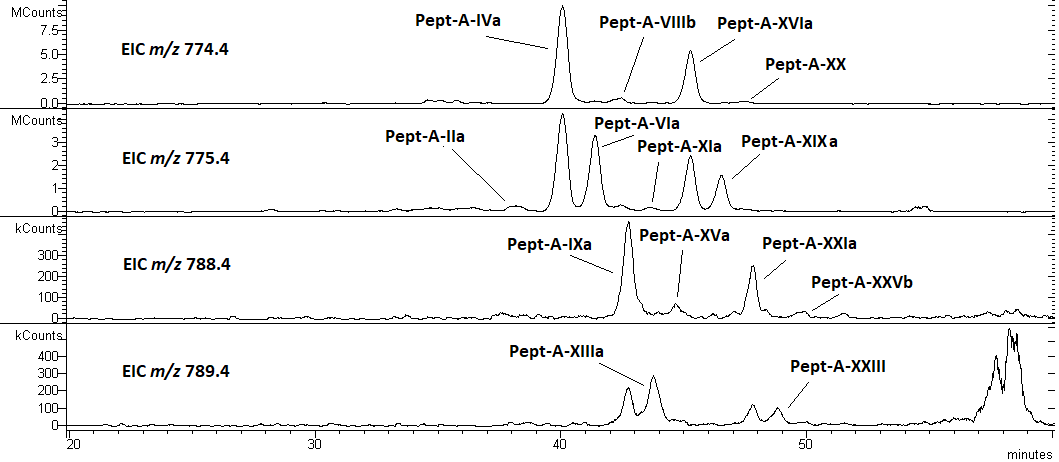
**

**Supplementary Figure 9 ǀ** Extracted ion chromatograms (EIC) resulting from full scan measurements of crude extracts from *T. longibrachiatum* SZMC 1775. The coeluting components within some peaks were identified based on MS^2^ experiments.

**
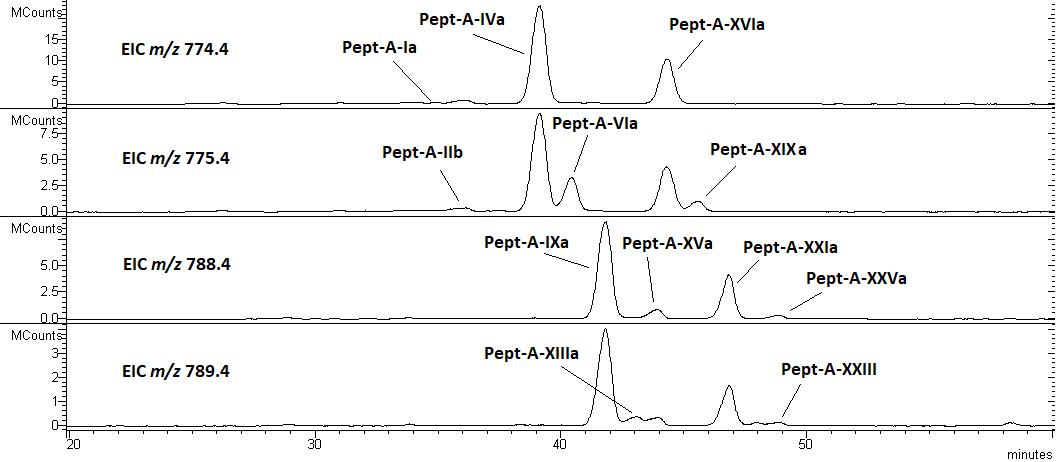
**

**Supplementary Figure 10 ǀ** Extracted ion chromatograms (EIC) resulting from full scan measurements of crude extracts from *T. longibrachiatum* SZMC 12546. The coeluting components within some peaks were identified based on MS^2^ experiments.

**
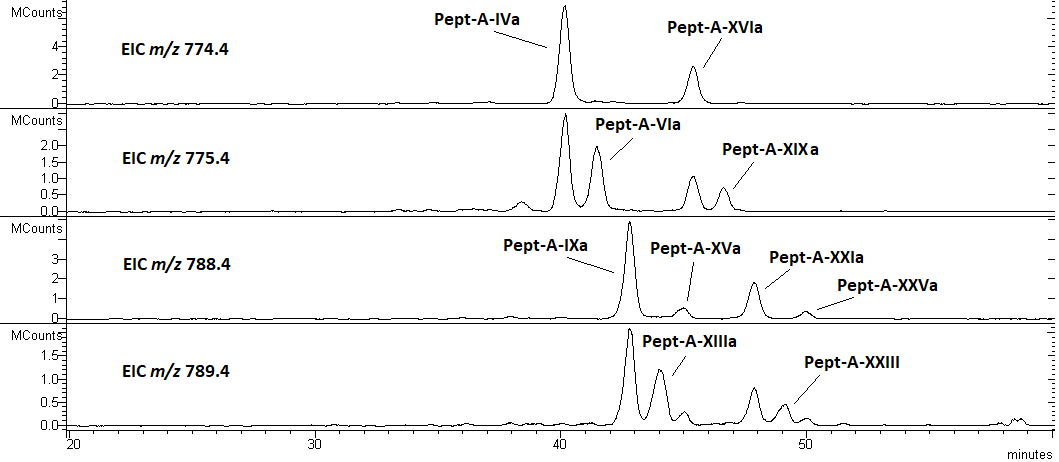
**

**Supplementary Figure 11 ǀ** Extracted ion chromatograms (EIC) resulting from full scan measurements of crude extracts from *T. capillare* SZMC 22605. The coeluting components within some peaks were identified based on MS^2^ experiments.

**
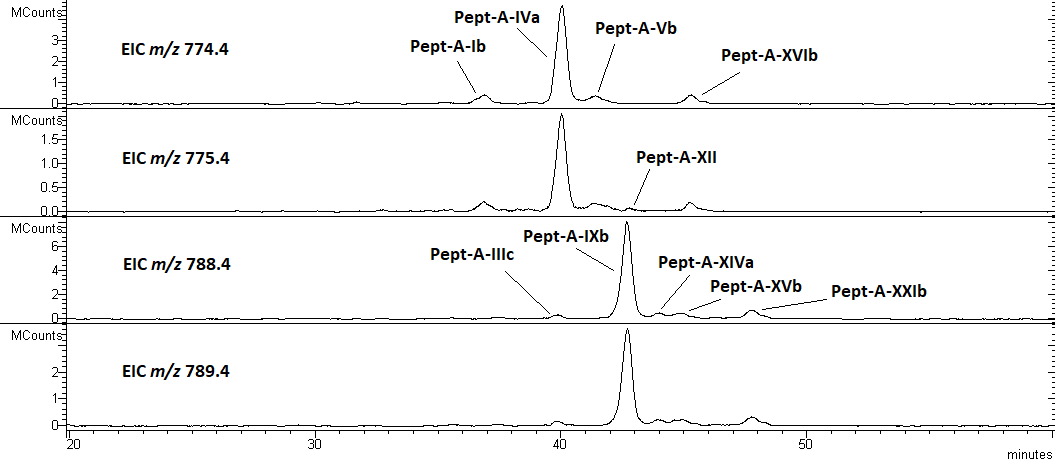
**

**Supplementary Figure 12 ǀ** Extracted ion chromatograms (EIC) resulting from full scan measurements of crude extracts from *T. novae-zelandiae* SZMC 22612. The coeluting components within some peaks were identified based on MS^2^ experiments.

**
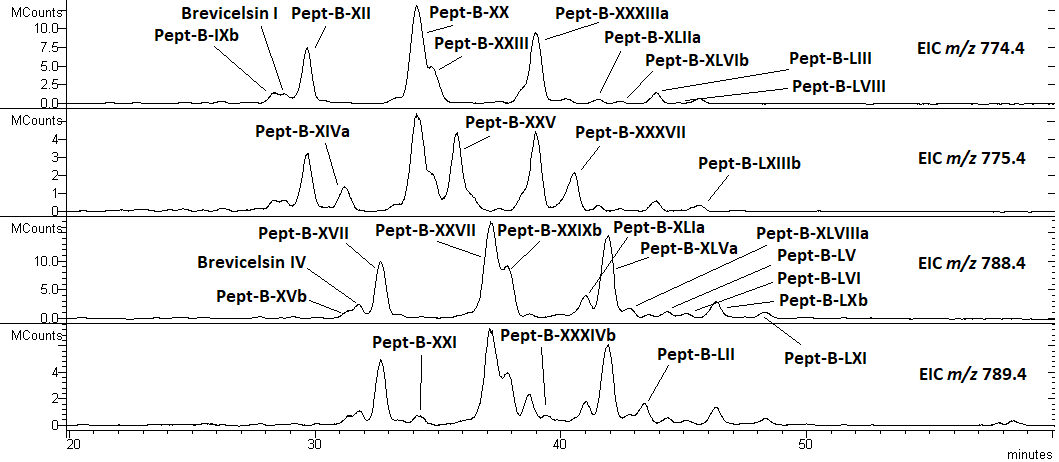
**

**Supplementary Figure 13 ǀ** Extracted ion chromatograms (EIC) resulting from full scan measurements of crude extracts from *T. parareesei* SZMC 22615. The coeluting components within some peaks were identified based on MS^2^ experiments.

**
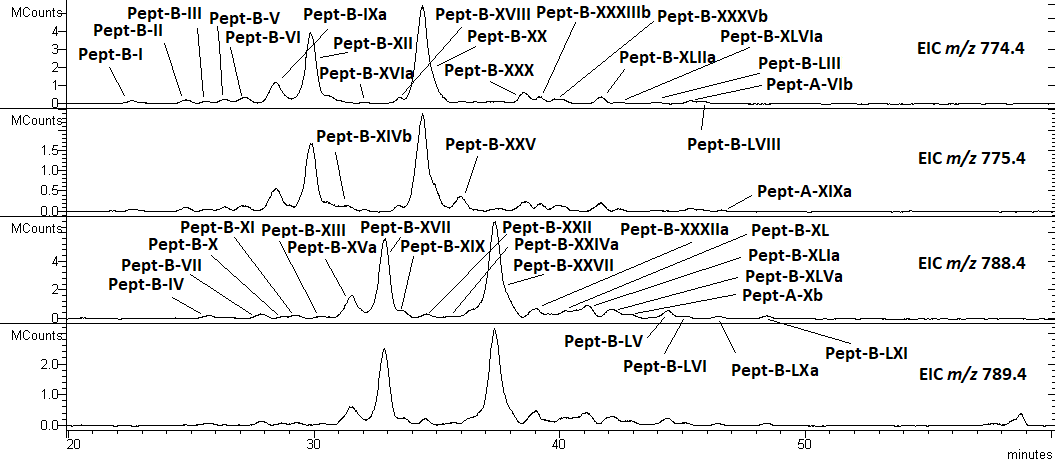
**

**Supplementary Figure 14 ǀ** Extracted ion chromatograms (EIC) resulting from full scan measurements of crude extracts from *T. reesei* SZMC 22614. The coeluting components within some peaks were identified based on MS^2^ experiments.

**
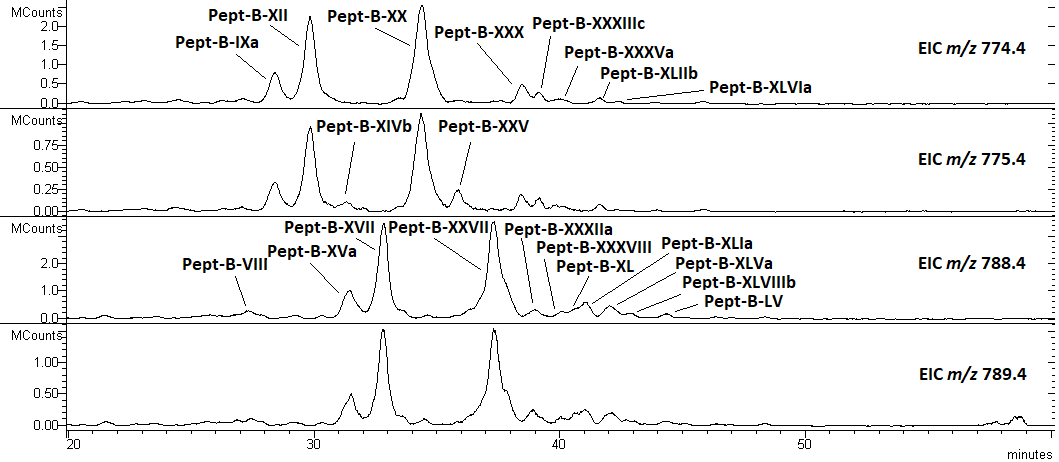
**

**Supplementary Figure 15 ǀ** Extracted ion chromatograms (EIC) resulting from full scan measurements of crude extracts from *T. reesei* SZMC 22616. The coeluting components within some peaks were identified based on MS^2^ experiments.

**
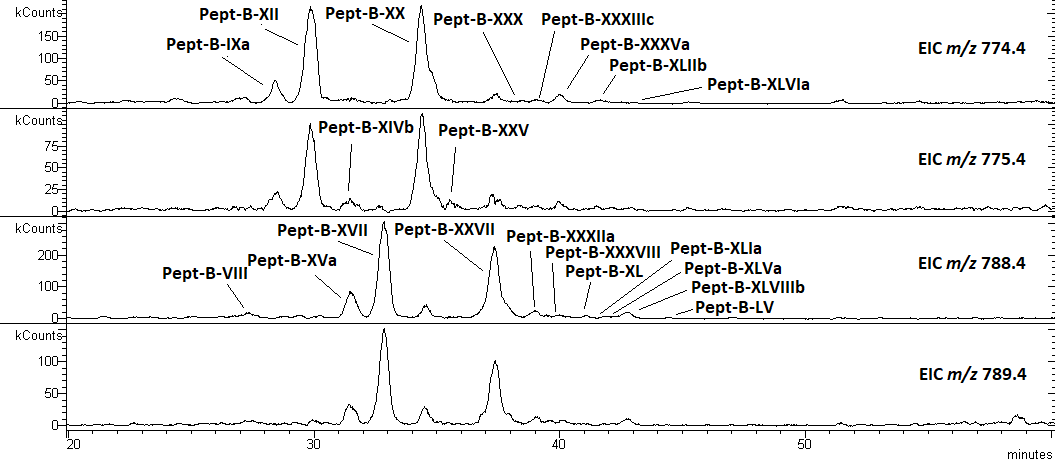
**

**Supplementary Figure 16 ǀ** Extracted ion chromatograms (EIC) resulting from full scan measurements of crude extracts from *T. reesei* SZMC 22617. The coeluting components within some peaks were identified based on MS^2^ experiments.

**
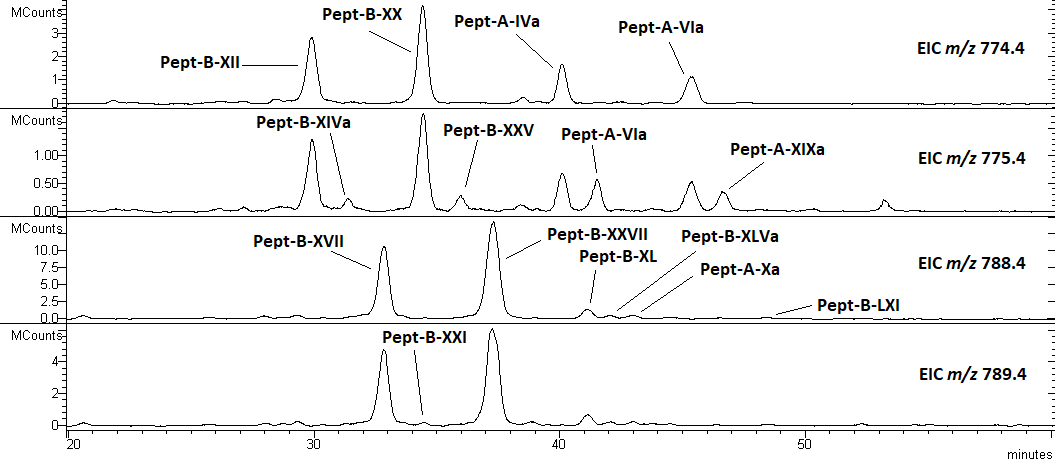
**

**Supplementary Figure 17 ǀ** Extracted ion chromatograms (EIC) resulting from full scan measurements of crude extracts from *T. saturnisporum* SZMC 22606. The coeluting components within some peaks were identified based on MS^2^ experiments.

**
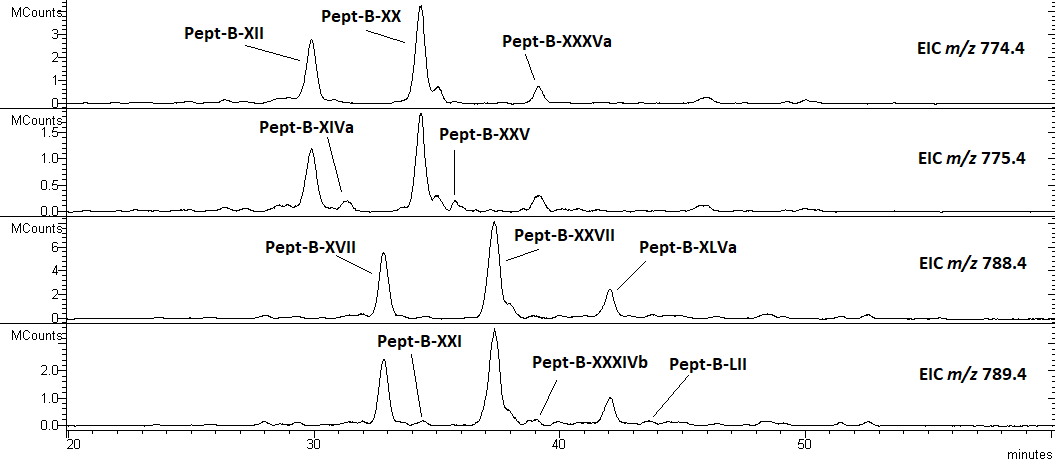
**

**Supplementary Figure 18 ǀ** Extracted ion chromatograms (EIC) resulting from full scan measurements of crude extracts from *T. effusum* SZMC 22611. The coeluting components within some peaks were identified based on MS^2^ experiments.

**
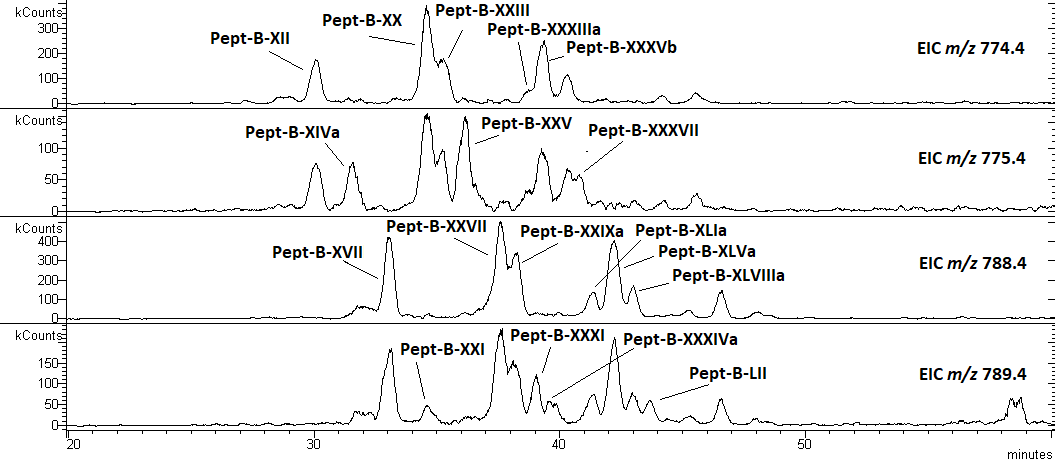
**

**Supplementary Figure 19 ǀ** Extracted ion chromatograms (EIC) resulting from full scan measurements of crude extracts from *T. andinense* SZMC 22610. The coeluting components within some peaks were identified based on MS^2^ experiments.

**
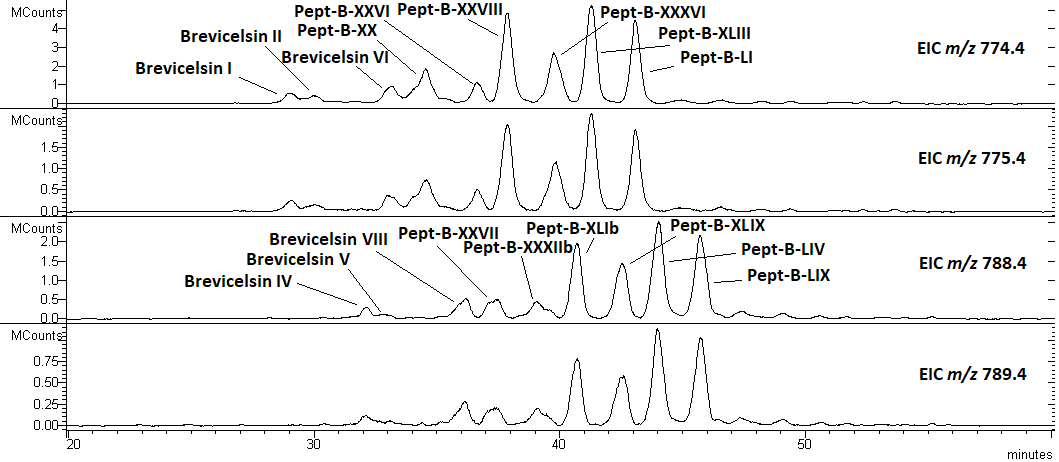
**

**Supplementary Figure 20 ǀ** Extracted ion chromatograms (EIC) resulting from full scan measurements of crude extracts from *T. sinense* SZMC 22609. The coeluting components within some peaks were identified based on MS^2^ experiments.

**
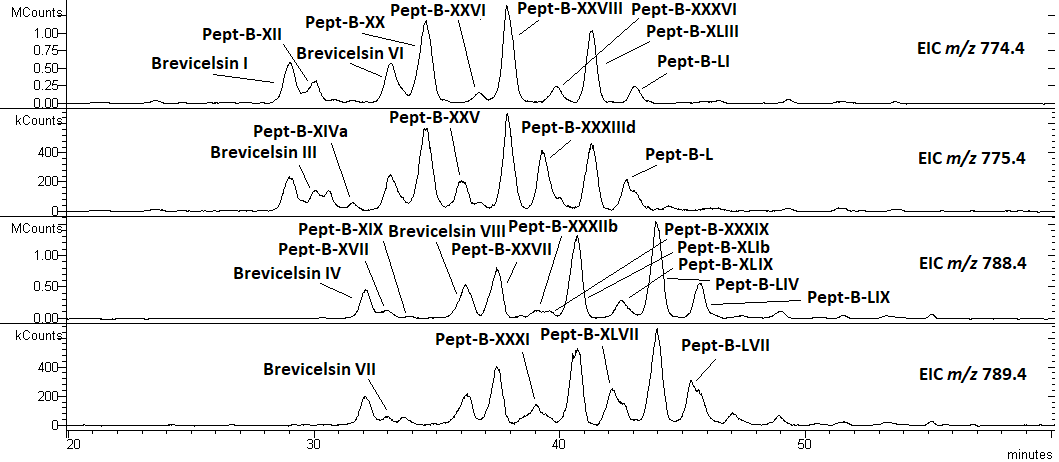
**

**Supplementary Figure 21 ǀ** Extracted ion chromatograms (EIC) resulting from full scan measurements of crude extracts from *T. flagellatum* SZMC 22608. The coeluting components within some peaks were identified based on MS^2^ experiments.

**
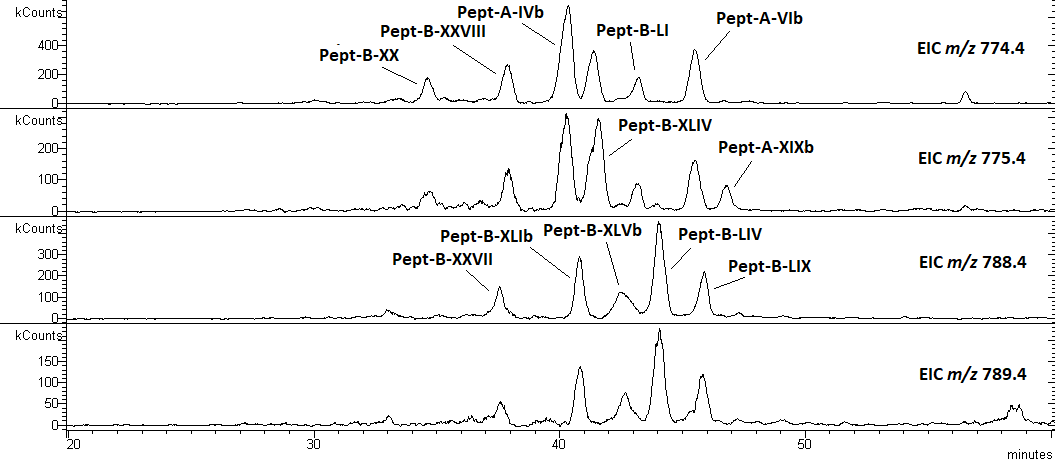
**

**Supplementary Figure 22 ǀ** Extracted ion chromatograms (EIC) resulting from full scan measurements of crude extracts from *T. konilangbra* SZMC 22607. The coeluting components within some peaks were identified based on MS^2^ experiments.

**
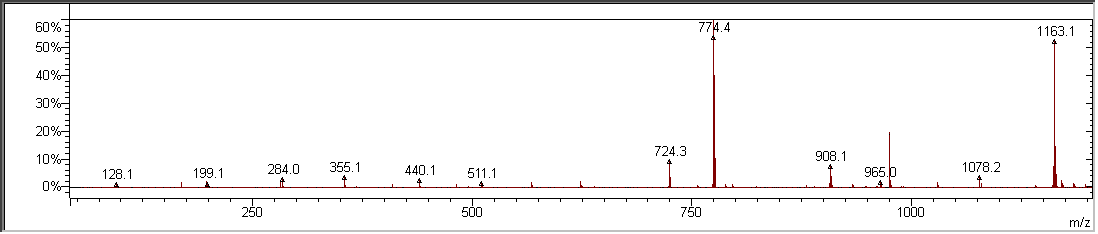
**

**A**


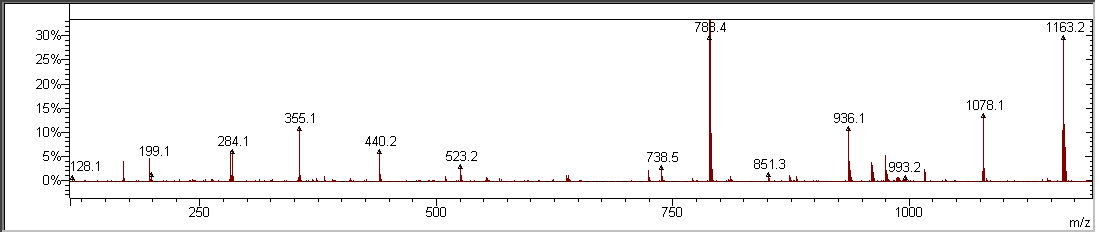


**B**


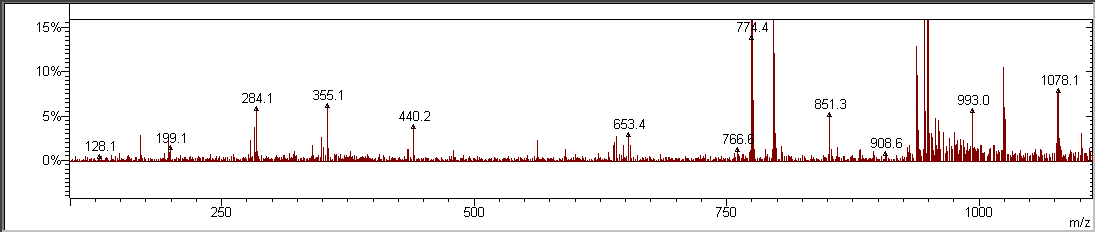


**C**

**Supplementary Figure 23 ǀ** Typical b-type ion series of three selected compounds: Pept-A-IVa (A), Pept-B-XXVII (B) from b_13_ at *m/z* 1163 and Brevicelsin I (C) ranged from b_13_ at *m/z* 1078 resulting from the full scan measurements of crude peptaibol extracts performed on Varian 500 MS system and prepared from *T. orientale* SzMC 12556, *T. saturnisporum* SzMC 22606 and *T. flagellatum* SzMC 22608, respectively.


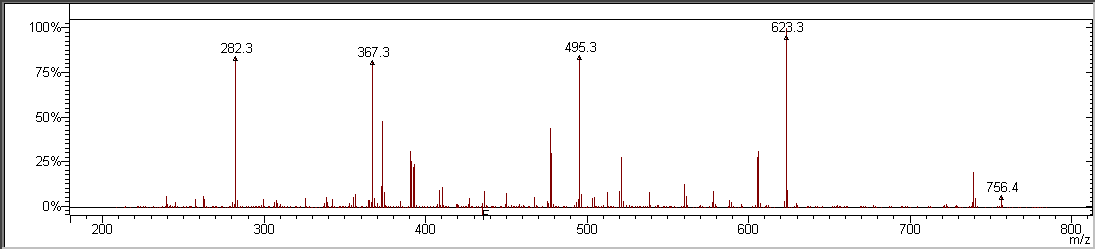


**A**


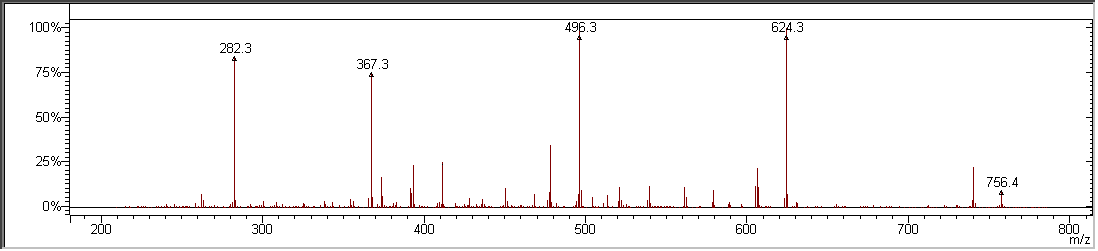


**B**


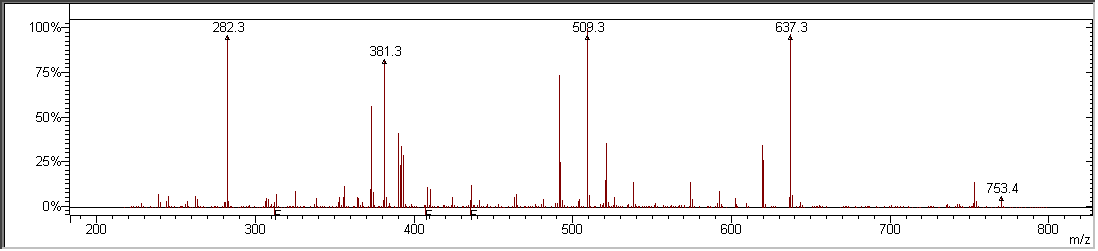


**C**


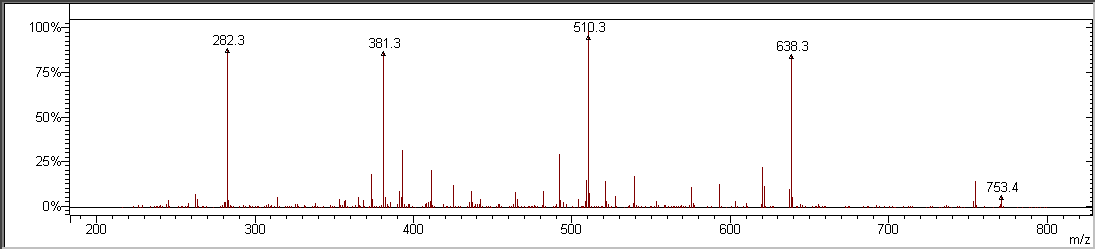


**D**

**Supplementary Figure 24 ǀ** Typical MS^2^ spectra of y-type ions *m/z* 774.4 (A), *m/z* 775.4 (B), *m/z* 788.4 (C) and *m/z* 789.4 (D) resulting from the full scan measurements of crude peptaibol extracts. Analyses were performed on Varian 500 MS system. MS^2^ spectra obtained from precursor ions of the same *m/z* ratio were identical.

**
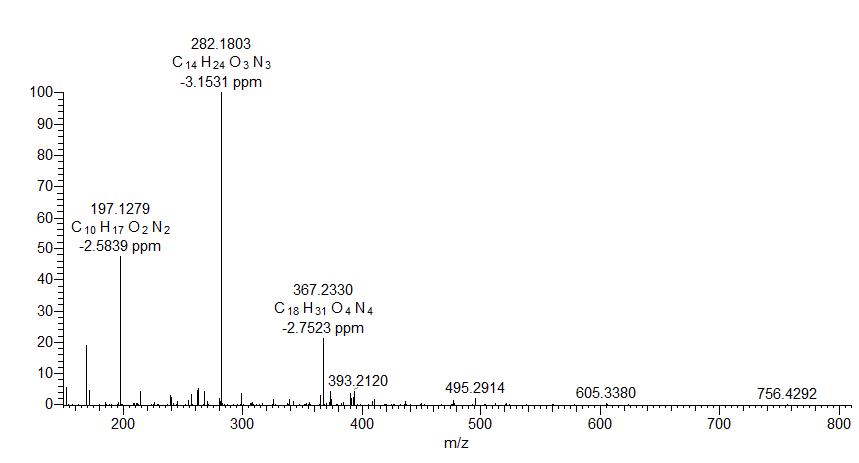

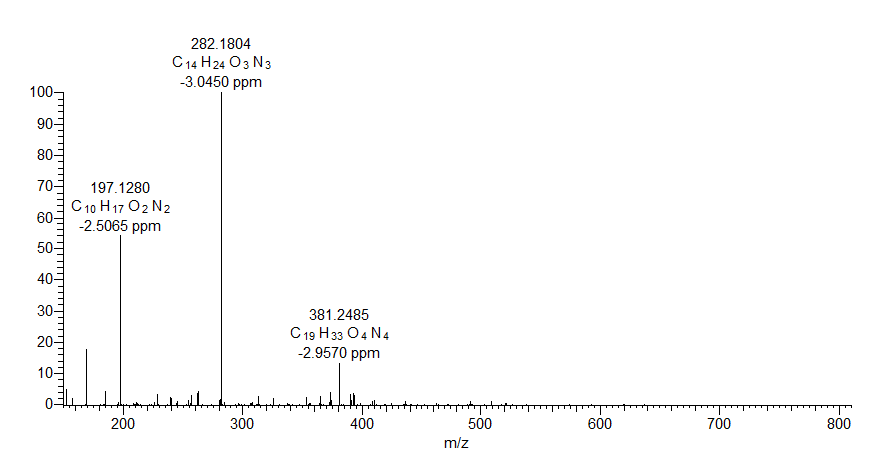
**

**B**

**A**

**Supplementary Figure 25 ǀ** Typical MS^2^ spectra of y-type ions *m/z* 774.4 (A) and *m/z* 788.4 (B) resulting from the full scan measurements of crude peptaibol extracts. Analyses were performed on Orbitrap-MS system. MS^2^ spectra obtained from precursor ions of the same *m/z* ratio were identical.
